# Paeoniflorin Upregulates Mitochondrial Thioredoxin of Schwann Cells to Improve Diabetic Peripheral Neuropathy Indicated by 4D Label-Free Quantitative Proteomics

**DOI:** 10.1155/2022/4775645

**Published:** 2022-03-18

**Authors:** Xinwei Yang, Xiao Li, Yanbo Zhu, YingYing Gao, Liping Xu

**Affiliations:** ^1^School of Traditional Chinese Medicine, Capital Medical University, Beijing 100069, China; ^2^Beijing Key Lab of TCM Collateral Disease Theory Research, Capital Medical University, Beijing 100069, China

## Abstract

Diabetic peripheral neuropathy (DPN) is a diabetic complication characterized by demyelination. The pathogenesis of DPN has not been fully elucidated, thus lacking therapies. In the current study, we aimed to confirm whether paeoniflorin (PF) could improve DPN by upregulating mitochondrial thioredoxin (Trx2) based on 4D Label-free proteomic experiments of Schwann cells (SCs) mitochondria. Firstly, PF increased the expression of mitochondrial processing peptidase *α* (Pmpca) and small ubiquitin-related modifier 1 (Sumo1) to increase mitochondrial protein processing of Trx2. Then, PF increased the protein expression of Trx reductase 2 (TrxR2) and peroxiredoxin 3 (Prx3), which belong to mitochondrial Trx systems. Accordingly, PF decreased mitochondrial reactive oxygen species (ROS) while increasing mtDNA and mitochondrial membrane potential to improve mitochondria function under high glucose environment. Furthermore, total glucosides of paeony capsules (TGP), containing more than 90% PF, increased the Trx2, TrxR2, and Prx3 levels in sciatic nerve of DPN rats, thus reducing demyelination as well as improving mechanical pain threshold, thermal pain threshold, motor nerve conduction velocity (MNCV), and sensor nerve conduction velocity (SNCV). Overall, these results suggest that PF could provide protection for DPN by upregulating Trx2.

## 1. Introduction

According to the International Diabetes Federation's prediction in 2021, diabetes is a significant global challenge to the health and well-being of individuals, families, and societies. More than 50% of diabetes will develop into DPN [1]. DPN can cause neuropathic pain and foot ulcers and is also the main cause of leg amputation. DPN seriously reduces the quality of life of patients and increases the mortality of diabetes. Despite decades of research, our understanding of the molecular mechanisms underlying is still in its infancy [2, 3]. Elucidating the complex pathogenesis and looking for effective treatment are still the focus of attention.

Hyperglycemia-induced excessive formation of mitochondrial ROS is considered a unified pathogenesis of diabetic complications including DPN [4]. Demyelination is one of the pathological features of DPN. ROS leads to mitochondrial dysfunction in SCs which has been proved to be one of the mechanisms of demyelination [5]. The previous studies of our research group confirmed that PF upregulated the endogenous antioxidant stress nuclear factor erythroid 2 related factor 2 (Nrf2) pathway of SCs in a high glucose environment to reduce mitochondrial apoptosis [6]. In addition, we have improved that PF could increase mitochondrial fusion and reduce mitochondrial fission to maintain the balance of mitochondrial dynamics (data was not shown).

PF can improve the mitochondrial function of SCs, suggesting that it may be an important substance to intervene in demyelination. However, the target of PF regulating mitochondrial function has not been fully understand. Whether PF can intervene DPN by improving mitochondrial function is an urgent scientific problem to be solved. Based on previous studies, we performed 4D Label-free proteomic experiments on the mitochondria of SCs. The mechanism and target of PF to improve mitochondrial function will be clarified, and the scientific basis for the development of new drugs for the intervention of DPN will be provided.

## 2. Materials and Methods

### 2.1. Cell Culture and Treatment

RSC96 cells (CRL-2765) were obtained from ATCC and cultured in DMEM (containing 25 mM glucose) supplemented with 10% FBS at 37°C in a humidified 5% CO_2_ atmosphere. The cells were treated with 150 mM glucose and 150 mM glucose pulsed 10 *μ*M paeoniflorin (PF) for 24 hours.

### 2.2. Mitochondrial Isolation

In RSC96 cells in T75 flask, mitochondrial fractions were prepared using the mitochondrial isolation kit for cultured cells (89874, Thermo Fisher). Briefly, 800 *μ*L reagent A and 10 *μ*L reagent B were added to the cells (2 × 10^7^) and then were incubated 5 minutes on ice. 800 *μ*L reagent C was added and centrifuged at 700 × *g* for 10 minutes at 4°C. The supernatant was collected and centrifuged at 12,000 × *g* for 15 minutes at 4°C. The supernatant was removed, and the pellet (mitochondria) was washed with 500 *μ*L of reagent C. Mitochondrial fraction was collected by centrifugation using a centrifuge at 12,000 × *g* for 5 minutes at 4°C.

### 2.3. 4D Label-Free Proteomic Analysis of Mitochondria

Mitochondrial sample was sonicated on ice using a high-intensity ultrasonic processor (Scientz) in lysis buffer (8 M urea and 1% Protease Inhibitor Cocktail) followed by centrifugation at 12,000 × *g* for 10 minutes at 4°C. Protein concentration was determined with BCA kit according to the manufacturer's instructions. For digestion, the protein solution was reduced with 5 mM dithiothreitol for 30 minutes at 56°C and alkylated with 11 mM iodoacetamide for 15 minutes at room temperature in darkness. The protein sample was then diluted by adding 100 mM TEAB to a urea concentration less than 2 M. Finally, trypsin was added at 1 : 50 trypsin-to-protein mass ratio for the first digestion overnight and 1 : 100 trypsin-to-protein mass ratio for the second 4-hour digestion. The tryptic peptides were analyzed on NanoElute UPLC coupled to timsTOF Pro (Bruker Daltonics, USA) mass spectrometer. Mass spectrometric measurements were carried out using the parallel accumulation serial fragmentation (PASEF) acquisition method. The obtained data were analyzed using MaxQuant version 1.6.5.0. Proteins with a fold change (FC) > 1.5 (or <0.6667) and *p* < 0.05 were regarded as differentially expressed proteins (DEPs). Detailed methods are described in Supplementary material 1.

### 2.4. Mitochondrial ROS

RSC96 cells (1^10^5^) in 30 mm confocal dish were treated with MitoSOX™ Red Mitochondrial Superoxide Indicator (600 nM, M36008, Thermo Fisher) and MitoTracker Green FM (200 nM, M7154, Thermo Fisher) for 30 minutes at 37°C in the dark. Fluorescent images were captured by Zeiss LSM 880 with Airyscan. Images were analyzed using Image-Pro Plus 6.0 software.

### 2.5. Mitochondrial Membrane Potential

RSC96 cells (1^10^5^) in 30 mm confocal dish were treated with Image-iT™ TMRM Reagent (200 nM, I34361, Thermo Fisher) and MitoTracker Green FM (200 nM, M7154, Thermo Fisher) for 30 minutes at 37°C in the dark. Fluorescent images were captured by Zeiss LSM 880 with Airyscan. Images were analyzed using Image-Pro Plus 6.0 software.

### 2.6. Mitochondrial DNA

RSC96 cells (1^10^5^) in a 30 mm confocal dish were incubated with MitoTracker TM Deep Red FM (200 nM, M22426, Thermo Fisher) for 30 min at 37°C in the dark. Then, the cells were fixed by 4% paraformaldehyde, permeabled membrane by 0.1% Triton X-100, and blocked by 3% BSA. The cells were incubated overnight with mtTFA antibody (1 : 500, ab176558, Abcam). In the next day, the cells incubated with the secondary antibody donkey anti-rabbit IgG-H&L (Alexa Fluor 488). DAPI was used to stain cell nuclei. Fluorescent images were captured by Zeiss LSM 880 with Airyscan. Images were analyzed using Image-Pro Plus 6.0 software.

### 2.7. Diabetic Rat Treatment

The protocol for using animals was approved by the Ethics Review Committee for Animal Experimentation of Capital Medical University (Ethical Inspection Number: AEEI-2018-144). Male Sprague-Dawley (SD) rats obtained from Experimental Animal Center at the Capital Medical University were randomly divided into three groups: normal, DPN, and total glucosides of paeony capsule (TGP, H20055058, Liwah Pharmaceutical) groups. The rats were fasted for 12 h and intraperitoneal injected of 60 mg/kg STZ (dissolved in 0.1 M citrate buffer, pH 4.5 at 4°C), while the rats in the normal group were injected only with citrate buffer. Fasting blood glucose (FBG) concentrations greater than or equal 16.7 mM after 72 h were treated as diabetes and were used for further experiments. This study uses the method of Yang et al. and the method description partly reproduces their wording [7–9]. The TGP group rats were intragastrically administered with TGP suspension (5.6 mg/kg/day), and the normal and DPN group rats were given equal volume distilled water everyday day for 12 weeks.

### 2.8. Thermal Sensitivity Measurements

Pain threshold to a thermal stimulus was assessed using a Tail-flick Analgesia Meter (YLS-12A, Yi Yan, Shandong, China). This study uses the method of Yang et al. and the method description partly reproduces their wording [7–9].

### 2.9. Mechanical Sensitivity Measurements

A series of Von Frey filaments (NC12773, Exacta) were employed to stimulate the plantar surface of the left hind paw with pressure. Paw withdrawal in response to each stimulus was recorded, and more than or equal 60% paw withdrawals were recorded as positive.

### 2.10. Electrophysiological Measurements

Motor nerve conduction velocity (MNCV) and sensory nerve conduction velocity (SNCV) in sciatic nerve were measured with Functional Experiment System (BL-420s, Taimeng, Sichuan, China). This study uses the method of Yang et al. and the method description partly reproduces their wording [7–9].

### 2.11. Ultrastructure Analysis

Ultrastructure myelination of sciatic nerve was analyzed by T transmission electron microscope (HT7700, Hitachi). This study uses the method of Yang et al. and the method description partly reproduces their wording [8].

### 2.12. Western Blot Analysis

Western blot analysis was performed according to the published protocols [7–9]. RSC96 cells or sciatic nerve tissues were harvested and the total proteins were extracted with the RIPA lysis buffer. The quantitative proteins were loaded onto 10% SDS-PAGE and wet-transferred to a 0.45 *μ*m PVDF membrane (Millipore, Temecula, CA, USA). At room temperature, the immunoblots were blocked by 5% nonfat milk for 1 h. Then, the immunoblots were probed with the target primary antibodies against Trx2 (1 : 2,000 dilution, ab185544, Abcam), Pmpca (1 : 1,000 dilution, sc-390471, Santa), Sumo1 (1 : 2,000 dilution, ab32058, Abcam), and TrxR2 (1 : 1,000 dilution, 16360-1-AP, Proteintech) at 4°C overnight. After incubation with the corresponding secondary antibodies for another 1 h at room temperature, the final results were quantified with ImageJ software.

### 2.13. Oxidative Phosphorylation (OXPHOS) Assay

Mitochondria of the cells were extracted using the mitochondrial isolation kit for cultured cells. Total OXPHOS rodent WB antibody cocktail (ab110413, Abcam) was used to analyze the relative levels of OXPHOS complex in mitochondria by Western blot.

### 2.14. Immunofluorescence Analysis

5 *μ*m thick transverse sections of sciatic nerve and cells were plated in a 30 mm confocal dish performed according to the published protocols [9]. Fluorescent images were captured by Leica TCS SP8 STED and Leica LAS image acquisition system. Image-Pro Plus 6.0 software was used for image analysis. The primary antibodies were TrxR2 (1 : 50 dilution, 16360-1-AP, Proteintech) and Prx3 (1 : 50 dilution, 10664-1-AP, Proteintech). Fluorescent images were captured by Zeiss LSM 880 with Airyscan. Images were analyzed using Image-Pro Plus 6.0 software.

### 2.15. Statistical Analysis

Data presentation and statistical analyses were carried out using Prism 7.04 software (GraphPad) and graphed as means ± standard error of the mean (S.E.M). Differences were analyzed by one-way ANOVA followed by Tukey's multiple comparisons test. *p* < 0.05 were considered statistically significant.

## 3. Results and Discussion

### 3.1. Proteomic Analyses Showed That Paeoniflorin Upregulated SC Mitochondrial Trx2 in a High Glucose Environment

**Figure 1:**
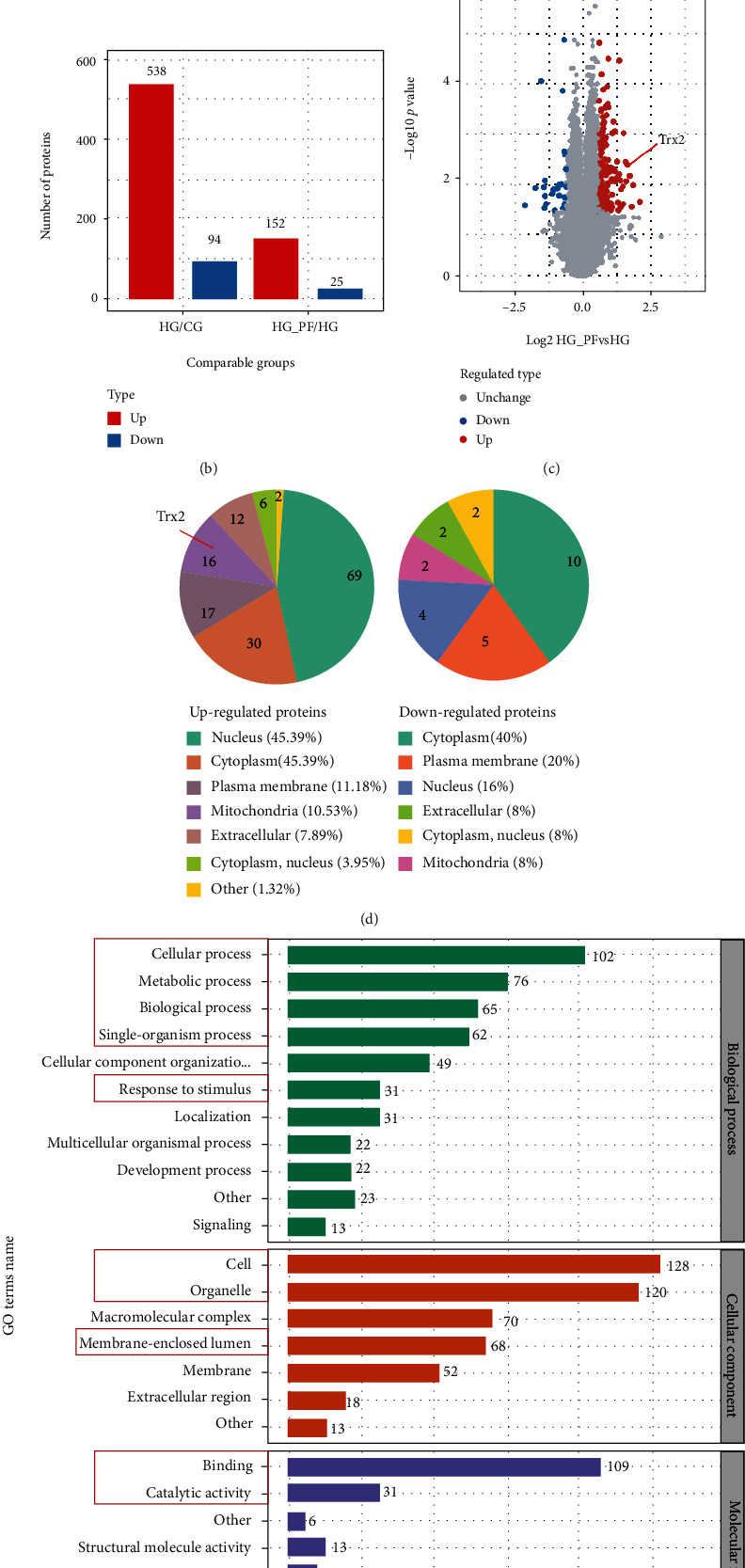
Identification of targeted protein Trx2 via 4D Label-free quantification. (a) The strategy for 4D Label-free quantification of the proteome of mitochondria in Schwann cells. (b) Histogram of the number distribution of differentially expressed proteins in different comparison groups. (c) Volcano plot of differentially expressed proteins. The red box indicates that it is related to Trx2. (d) Subcellular localization chart of differentially expressed proteins in mitochondria (HG_PF/HG). (e) Statistical distribution chart of upregulated proteins under each GO category (2nd level) (HG_PF/HG).

Protein extracts from mitochondria of RSC96 cells were collected for 4D Label-free analysis (Figure 1(a)). In total, we found that there were 177 DEPs (HG_PF/HG), including 152 upregulated proteins and 25 downregulated proteins (Figure 1(b)). Among them, there were 16 upregulated proteins and 2 downregulated protein subcellular located in mitochondria (Figure 1(d) and Table 1). Among these differentially expressed proteins, Trx2 was remarkably upregulated in mitochondria (FC = 3.112, *p* = 0.005) (Figure 1(c)) while downregulated in 25 mM glucose group compared to 150 mM glucose groups (FC = 0.664, *p* = 0.042). DEPs were classified by Gene Ontology annotation based on three categories: biological process, cellular component, and molecular function. Trx2 was related to cellular process, metabolic process, biological regulation, single-organism process, and response to stimulus. The molecular functions of Trx2 were known as binding and catalytic activity (Figure 1(e)).

In this experiment, only 18 mitochondrial proteins met the criteria for DEPs of this proteomic analysis. This subcellular localization information given by proteomics is based on the subcellular structure prediction software wolfpsort. In Gene Ontology (GO) assignments “mitochondrion” and MitoCarta3.0, we identified 1,072 mitochondrial proteins (Supplementary material 2). This is consistent with others showing that widely used data repositories taken together can identify more mitochondrial proteins [10]. We found that the extracted proteins also contained proteins from other organelle sources. The high sensitivity of mass spectrometry- (MS-) based approaches leads to the identification of even minor contaminations of organelle preparations, and thus, the numerous lists of identified proteins contain a mixture of authentic mitochondrial proteins, loosely attached proteins, and contaminants [11]. In addition to proteomic analysis based on purified mitochondrial fraction, subtractive proteomic experiments and integrative mitochondrial proteomic methods were reported to try to get a high-confidence mitochondrial proteome [12].

From the previous results, we observed that PF increased Nrf2 protein level and nuclear translocation to reduce mitochondrial oxidative stress and damage [6]. Recent research indicated that disrupting mitochondrial thiol redox homeostasis leads to Nrf2 activation [13]. The mitochondrial Trx system is one of the mitochondrial thiol redox systems. The above analysis indicates that PF may increase Nrf2 activation by upregulating Trx2.

Moreover, Trx2 plays important roles in mitochondrial functions in view of the proposed specific interactions [14]. In the analysis of the reported mitochondrial interactome of Trx2 and our Trx2-interacting proteins by proteomics of mitochondria in SCs, PF could upregulate 11 proteins and downregulate 6 proteins (Table 2). These proteins involve in TCA cycle, glycolysis, amino acid metabolism, mitochondrial membrane integrity, mitochondrial protein synthesis, protein mitochondrial import and folding, and oxidative stress [14–16].

Based on the results of our previous study and these reports, we believe that PF can improve mitochondrial function by upregulating Trx2 as well as regulating its mitochondrial interactome. To test our hypothesis, we performed experiments on measuring the expression of Trx2.

### 3.2. Paeoniflorin Enhances Mitochondrial Function in SCs by Upregulating Trx2

**Figure 2:**
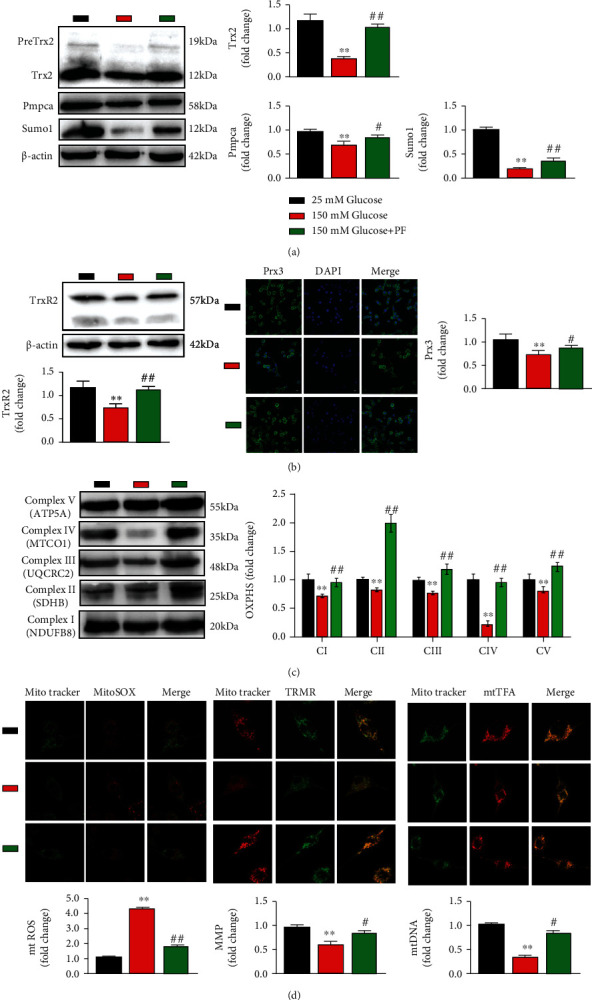
PF enhances mitochondrial function in SCs by upregulating Trx2. (a) Effect of PF on mitochondrial protein processing of Trx2. Trx2, Pmpca, and Sumo1 were measured by Western blot. *n* = 3. (b) Effect of PF on antioxidative stress of Trx2. TrxR2 was measured by Western blot and Prx3 was measured by immunofluorescence analysis. Histogram of the number distribution of differentially expressed proteins in different comparison groups. *n* = 3. (c) Effect of PF on OXPHS. (d) Effect of PF on mitochondrial function. mtROS, MMP, and mtDNA were examined by immunofluorescence staining of live cells. Scale bar represent 5 *μ*m. *n* = 3. ^∗∗^*p* < 0.01 vs. 25 mM glucose group; ^#^*p* < 0.05 and ^##^*p* < 0.01 vs. 150 mM glucose group.

Trx2 exerts antioxidant stress and maintains mitochondrial function, which depends on the small ubiquitin-related modifier (SUMO)ylated mitochondrial processing peptidase (MPP) including MPP*α*, also known as Pmpca, to regulate its protein processing in mitochondria [17, 18]. The unprocessed Trx2 (about 23 kDa) in the cytoplasm is transported to the mitochondrial matrix through the mitochondrial outer membrane transposase complex and the mitochondrial inner membrane transposase complex under the action of the N-terminal guiding sequence mitochondrial targeting signal peptide (MTS) [19]. However, Trx2 precursor proteinpre (PreTrx2, 18 kDa) does not work only when SUMOylated Pmpca combines with SUMO interaction motifs (SIMs) of Trx2 and cuts the N-terminal presequence in MTS to form mature Trx2 (mature trx2, 12 kDa) [15]. We first examined whether PF affected the mitochondrial protein processing of Trx2. Our results showed that PF increased the expression of mature Trx2 in SCs under high glucose environment, consistent with increasing the expression of Pmpca and Sumo1, thus PF increasing the protein processing of Trx2 in mitochondria (Figure 2(a)).

The mitochondrial Trx system consists of Trx2 and Trx reductase 2 (TrxR2), which maintains Trx2 in a reduced state by using mitochondrial NADPH as a substrate. Trx2 maintains the activities of the peroxidase Peroxiredoxin 3 (Prx3) [19]. To determine whether PF increases the mitochondrial Trx system, we further assessed the levels of TrxR2 and Prx3. The data showed that PF increased the protein expression of TrxR2 and Prx3 (Figure 2(b)).

Mitochondria are involved in ATP production through oxidative phosphorylation (OXPHOS) [20]. Trx2 was involved in the functional and structural integrity of the electron transport chain (ETC) related to OXPHOS. Mitochondrial genomes (mtDNA) encode proteins in ETC. Furthermore, the ETC makes mitochondria an important organelle involved in producing ROS [21]. Trx2 can directly act as a ROS scavenger and increases the mitochondrial membrane potential (MMP) [22]. Our results showed that PF increased OXPHOS under high glucose environment (Figure 2(c)). Meanwhile, PF increased mtDNA and MMP while decreased mtROS (Figure 2(d)). We conclude that PF enhanced mitochondrial function through upregulating Trx2 due to increased mitochondrial protein processing of Trx2, direct antioxidant stress, and mitochondrial interactome of Trx2.

### 3.3. TGP Improvement Nerve Function in DPN Rats Is Related to the Upregulation of Trx2

Trx2 plays an important role in antioxidative stress and maintenance of mitochondrial function. It has been confirmed that Trx2 can provide protection for diabetes and complications such as diabetic cardiomyopathy and diabetic retinopathy [23, 24]. Whether Trx2 can protect DPN has not been reported. Schwann cells (SCs) are the most abundant cells in the peripheral nervous system and belong to myelin forming cells. Mitochondrial dysfunction in SCs induces demyelination, which is one of the main pathogenesis of DPN [25]. This study has confirmed that PF upregulates Trx2 of SCs in a high glucose environment to improve mitochondrial function. We further studied whether PF can improve DPN and whether it is related to the upregulation of Trx2.

**Figure 3:**
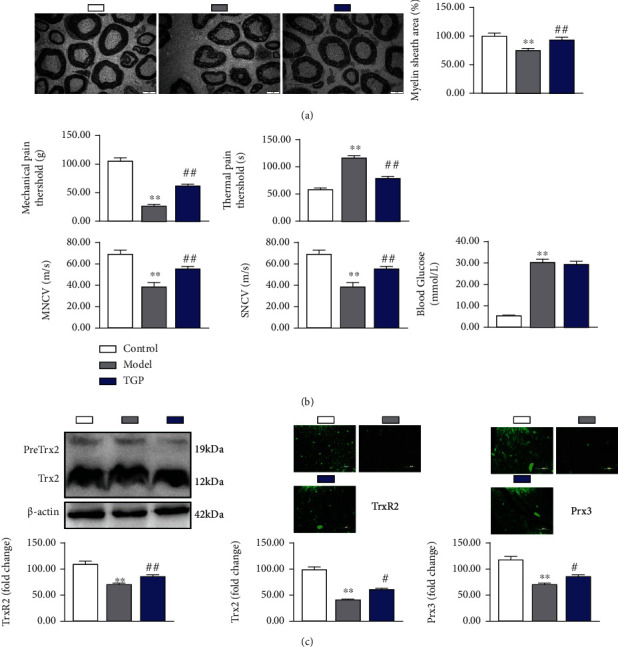
TGP improvement nerve function in DPN rats is related to the upregulation of Trx2. (a) Effect of TGP on myelin sheath area of sciatic nerve in DPN rats. Scale bar represent 2 *μ*m. *n* = 6. (b) Effect of TGP on nerve function including mechanical pain threshold, thermal pain threshold, MNCV, and SNCV. *n* = 8. (c) Effect of TGP on Trx2, TrxR2, and Prx3 levels. Scale bar represent 50 *μ*m. *n* = 4. ^∗∗^*p* < 0.01 vs. control group; ^#^*p* < 0.05 and ^##^*p* < 0.01 vs. model group.

In this part of the experiment, we used TGP to intervene DPN rats. TGP is an effective component extracted from the dried root of Paeonia lactiflora Pall. PF accounts for more than 90% of the total glycosides and is the main active component of TGP [26]. Our results showed that TGP increased the myelin sheath of DPN rats (Figure 3(a)). It is suggested that TGP can improve mitochondrial dysfunction of SCs to alleviate demyelination. We further examined nerve function represented by mechanical pain threshold, thermal pain threshold, motor nerve conduction velocity (MNCV), and sensor nerve conduction velocity (SNCV). The data showed that TGP increased the mechanical pain threshold, MNCV, and SNCV while decreased thermal pain threshold, thus improving peripheral nerve function in DPN rats (Figure 3(b)).

To investigate whether the protection of nerve function was associated with regulating Trx2, we analyzed Trx2, TrxR2, and Prx3 levels in the sciatic nerve of DPN rats. Trx2, TrxR2, and Prx3 were increased in the TGP group compared to the model group (Figure 3(c)). In summary, we have shown that TGP can provide protection for DPN by upregulating Trx2.

## 4. Conclusions

In conclusion, the current study revealed that PF upregulated the Trx2 through increasing its mitochondrial protein processing. Enhanced mitochondrial Trx2 improved mitochondrial function of SCs under high glucose environment. This may be one of the mechanisms of PF intervention in DPN. In addition, Trx2 possesses the potential to be a useful molecular target in the clinical treatment of DPN. The inadequacy of our experiments is lacking of research on mitochondrial interactome of Trx2 in DPN, which would be the goal of our subsequent studies.

## Figures and Tables

**Table 1:** Mitochondrial localization of differentially expressed proteins by PF.

Protein accession	Protein description	HG_PF/HG ratio	Regulated type	HG_PF/HG *p* value	Gene name
P97615	Thioredoxin, mitochondrial	3.112	Up	0.005	Txn2 (Trx2)
Q5BJT6	Large subunit GTPase 1 homolog	4.244	Up	0.031	Lsg1
Q5M818	39S ribosomal protein L16, mitochondrial	2.969	Up	0.005	Mrpl16
P61751	ADP-ribosylation factor 4	2.098	Up	0.022	Arf4
Q5RK08	Glioblastoma amplified sequence	2.031	Up	0.045	Nipsnap2
Q3B8R7	Mitochondrial ribosomal protein L47	1.938	Up	0.037	Mrpl47
D3ZIN7	Mitochondrial ribosomal protein	1.726	Up	0.050	Mrps23
Q5M7W7	Probable proline--tRNA ligase, mitochondrial	1.647	Up	0.020	Pars2
Q6P9Y4	ADP/ATP translocase 1	1.580	Up	0.0004	Slc25a4
B2RZD1	Protein transport protein Sec61 subunit beta	1.570	Up	0.030	Sec61b
D3ZU04	Uncharacterized protein	1.562	Up	0.023	—
P26772	10 kDa heat shock protein, mitochondrial	1.548	Up	0.006	Hspe1
G3V8Z3	G patch domain and KOW motifs	1.539	Up	0.033	Gpkow
D3ZF13	Acyl carrier protein	1.523	Up	0.002	Ndufab1
B0BN72	Mapk-regulated corepressor-interacting protein 1	1.513	Up	0.048	Mcrip1
D3ZYL4	Mitochondrial ribosomal protein L50	1.506	Up	0.020	Mrpl50
A0A0G2K9E5	Leucyl-tRNA synthetase 2, mitochondrial	0.603	Down	0.0002	Lars2
A0A0G2K1W9	Lactate dehydrogenase D	0.380	Down	0.041	Ldhd

**Table 2:** Mitochondrial interactome of Trx2 in rat Schwann cells regulated by PF.

Protein accession	Protein description	HG_PF/HG ratio	Regulated type	HG_PF/HG *p* value	Gene name
B2RZD6	NDUFA4, mitochondrial complex-associated	2.623	Up	0.0172	Ndufa4
A0A0H2UHT6	40S ribosomal protein S18	1.641	Up	0.0015	Rps18
P20788	Cytochrome b-c1 complex subunit Rieske, mitochondrial	1.398	Up	0.0091	Uqcrfs1
P15999	ATP synthase subunit alpha, mitochondrial	1.21	Up	0.0218	Atp5f1a
O88767	Protein/nucleic acid deglycase DJ-1	1.195	Up	0.0225	Park7
Q66HF1	NADH-ubiquinone oxidoreductase 75 kDa subunit, mitochondrial	1.189	Up	0.0269	Ndufs1
Q75Q41	Mitochondrial import receptor subunit TOM22 homolog	1.154	Up	0.0025	Tomm22
F1M953	Stress-70 protein, mitochondrial	1.154	Up	0.0062	Hspa9
Q5XIJ3	Isocitrate dehydrogenase [NAD] subunit, mitochondrial	1.133	Up	0.0145	Idh3g
A0A0G2JVH4	MICOS complex subunit MIC60	1.127	Up	0.0036	Immt
Q9ER34	Aconitate hydratase, mitochondrial	1.055	Up	0.0306	Aco2
P04785	Protein disulfide-isomerase	0.931	Down	0.0044	P4hb
Q9Z0V5	Peroxiredoxin-4	0.884	Down	0.0019	Prdx4
O35244	Peroxiredoxin-6	0.863	Down	0.0048	Prdx6
P07632	Superoxide dismutase [Cu-Zn]	0.777	Down	0.0388	Sod1
Q5XIT9	Methylcrotonoyl-CoA carboxylase beta chain, mitochondrial	0.733	Down	0.0222	Mccc2
A0A0G2K1W9	Lactate dehydrogenase D	0.38	Down	0.0414	Ldhd

## Data Availability

The data used to support the findings of this study are available from the corresponding author upon request.

## Supplementary Materials

Supplementary Materials Experimental procedures of 4D Label-free proteomic analysis of mitochondria were shown in Supplementary Materials 1. Proteins in mitochondria identified by Gene Ontology and MitoCarta 3.0 are shown in Supplementary Materials 2.
