# Supplementary material for: Paeoniflorin Upregulates Mitochondrial Thioredoxin of Schwann Cells to Improve Diabetic Peripheral Neuropathy Indicated by 4D Label-Free Quantitative Proteomics
Source: Oxid Med Cell Longev. 2022 Mar 18;2022:4775645. doi: 10.1155/2022/4775645 (PMC8956397; doi:10.1155/2022/4775645)
Supplement: Supplementary Materials — Experimental procedures of 4D Label-free proteomic analysis of mitochondria were shown in Supplementary Materials 1. Proteins in mitochondria identified by Gene Ontology and MitoCarta 3.0 are shown in Supplementary Materials 2. [file 4775645.f1.docx]

# Supplementary material 1. Experimental Procedures

- 1. **Protein Extraction**

Sample was sonicated three times on ice using a high intensity ultrasonic processor (Scientz) in lysis buffer (8 M urea, 1% Protease Inhibitor Cocktail). The remaining debris was removed by centrifugation at 12,000 g at 4 °C for 10 min. Finally, the supernatant was collected and the protein concentration was determined with BCA kit according to the manufacturer’s instructions.

**1.2 Trypsin Digestion**

For digestion, the protein solution was reduced with 5 mM dithiothreitol for 30 min at 56 °C and alkylated with 11 mM iodoacetamide for 15 min at room temperature in darkness. The protein sample was then diluted by adding 100 mM TEAB to urea concentration less than 2M. Finally, trypsin was added at 1:50 trypsin-to-protein mass ratio for the first digestion overnight and 1:100 trypsin-to-protein mass ratio for a second 4 h-digestion.

**1.3 LC-MS/MS Analysis**

Peptides were dissolved by liquid chromatography for flow phase A phase (0.1% (v / v) aqueous formic acid solution) and separated using a NanoElute ultra-high performance liquid phase system.The flow phase A is an aqueous solution containing 0.1% formic acid, and the flow phase B is an acetonitrile solution containing 0.1% formic acid.Set of liquid phase gradient: 0-70 min, 6%~22%B; 70-84 min, 22%~32%B; 84-87 min, 32%~80%B; 87-90 min, 80%B, and the flow rate was maintained at 300 nL / min.

Peptides were separated via an ultra-high performance liquid phase system and injected into Capillary ion sources for ionization and then into tims-TOF Pro mass spectrometry for analysis.The ion source voltage was set to 1.4 kV, and both the peptide parent ions and their secondary fragments were detected and analyzed using TOF.Secondary mass spectrometry scan range was set to 100-1700 m / z.The data acquisition mode uses the Parallel cumulative Serial Fragmentation (PASEF) mode.A secondary spectrogram with the number of parent ion charges obtained after 10 times of PASEF mode acquisition ranged from 0-5, and the dynamic exclusion time for tandem MS scans was set to 24s

**1.4 Database Search**

Secondary mass spectrometry data were retrieved using Maxquant (v1.6.5.0).Retrieval parameter settings: The database is UniPort Rattus norvegicus (29,955 sequences), The reverse library was added to calculate the false positive rate (FDR) caused by random matching, And common pollution libraries are added to the database, Use to eliminate the effects of contaminating proteins in the identification results; The digestion mode was set to Trypsin / P; The number of missed cut sites was set to 2; The primary mother ion mass error tolerance for First search and Main search was set to 70 ppm and 70 ppm, respectively, The mass error tolerance of the secondary fragment ions is 0.04 Da.Cysteine alkylation was set to a fixed modification, variable modification to the oxidation of methionine, and acetylation at the N terminus of the protein.The FDR for protein identification and PSM identification was all set to 1%.

1. **Bioinformatics Methods**

**2.1 Annotation Methods**

**GO Annotation**

Gene Ontology (GO) annotation proteome was derived from the UniProt-GOA database ([www. http://www.ebi.ac.uk/GOA/](file:///C:\\Users\\BioPTM\\Desktop\\PTM_0078_iTRAQ_report\\PTM_0078_iTRAQ_report\\www.%20http:\\www.ebi.ac.uk\\GOA\\)). Firstly, Converting identified protein ID to UniProt ID and then mapping to GO IDs by protein ID. If some identified proteins were not annotated by UniProt-GOA database, the [InterProScan](http://www.ebi.ac.uk/interpro/) soft would be used to annotated protein’s GO functional based on protein sequence alignment method. Then proteins were classified by [Gene Ontology annotation](http://www.geneontology.org/) based on three categories: biological process, cellular component and molecular function.

**Domain Annotation**

Identified proteins domain functional description were annotated by [InterProScan](http://www.ebi.ac.uk/interpro/) (a sequence analysis application) based on protein sequence alignment method, and the InterPro domain database was used. InterPro (http://www.ebi.ac.uk/interpro/) is a database that integrates diverse information about protein families, domains and functional sites, and makes it freely available to the public via Web-based interfaces and services. Central to the database are diagnostic models, known as signatures, against which protein sequences can be searched to determine their potential function. InterPro has utility in the large-scale analysis of whole genomes and meta-genomes, as well as in characterizing individual protein sequences.

**KEGG Pathway Annotation**

[Kyoto Encyclopedia of Genes and Genomes (KEGG)](http://www.genome.jp/kegg/) database was used to annotate protein pathway. Firstly, using KEGG online service tools KAAS to annotated protein’s KEGG database description. Then mapping the annotation result on the KEGG pathway database using KEGG online service tools KEGG mapper.

**Subcellular Localization**

There, we used [wolfpsort](http://wolfpsort.seq.cbrc.jp/) a subcellular localization predication soft to predict subcellular localization. [Wolfpsort](http://wolfpsort.seq.cbrc.jp/) is an updated version of PSORT/PSORT II for the prediction of eukaryotic sequences. Special for protokaryon species, Subcellular localization prediction soft CELLO was used.

**2.2 Functional Enrichment**

**Enrichment of Gene Ontology analysis**

Proteins were classified by GO annotation into three categories: biological process, cellular compartment and molecular function. For each category, a two-tailed Fisher’s exact test was employed to test the enrichment of the differentially expressed protein against all identified proteins. The GO with a corrected p-value < 0.05 is considered significant.

**Enrichment of pathway analysis**

Encyclopedia of Genes and Genomes (KEGG) database was used to identify enriched pathways by a two-tailed Fisher’s exact test to test the enrichment of the differentially expressed protein against all identified proteins. The pathway with a corrected p-value < 0.05 was considered significant. These pathways were classified into hierarchical categories according to the KEGG website.

**Enrichment of protein domain analysis**

For each category proteins, InterPro (a resource that provides functional analysis of protein sequences by classifying them into families and predicting the presence of domains and important sites) database was researched and a two-tailed Fisher’s exact test was employed to test the enrichment of the differentially expressed protein against all identified proteins. Protein domains with a p-value < 0.05 were considered significant.

**2.3 Enrichment-based Clustering**

For further hierarchical clustering based on different protein functional classification (such as: GO, Domain, Pathway, Complex). We first collated all the categories obtained after enrichment along with their P values, and then filtered for those categories which were at least enriched in one of the clusters with P value <0.05. This filtered P value matrix was transformed by the function x = −log10 (P value). Finally these x values were z-transformed for each functional category. These z scores were then clustered by one-way hierarchical clustering (Euclidean distance, average linkage clustering) in Genesis. Cluster membership were visualized by a heat map using the “heatmap.2” function from the “gplots” R-package.

**2.4 Protein-protein Interaction Network**

All differentially expressed protein database accession or sequence were searched against the STRING database version 10.5 for protein-protein interactions. Only interactions between the proteins belonging to the searched data set were selected, thereby excluding external candidates. STRING defines a metric called “confidence score” to define interaction confidence; we fetched all interactions that had a confidence score >0.7 (high confidence). Interaction network form STRING was visualized in R package “networkD3”.

# Supplementary Materials 2.Proteins in mitochondria identified by Gene ontology and MitoCarta 3.0.

| **Protein accession** | **Protein description** | **Gene name** | **Sequence coverage [%]** | **Mol. weight [kDa]** | **Score** |
| --- | --- | --- | --- | --- | --- |
| B2RYW8 | MICOS complex subunit Mic10 OS=Rattus norvegicus OX=10116 GN=Minos1 PE=3 SV=1 | Minos1 | 42.1 | 8.5498 | 99.279 |
| A0A1B0GWV4 | Translocase of outer mitochondrial membrane 5 OS=Rattus norvegicus OX=10116 GN=Tomm5 PE=1 SV=1 | Tomm5 | 42.6 | 5.4384 | 3.4828 |
| P27867 | Sorbitol dehydrogenase OS=Rattus norvegicus OX=10116 GN=Sord PE=1 SV=4 | Sord | 24.6 | 38.234 | 27.975 |
| P04762 | Catalase OS=Rattus norvegicus OX=10116 GN=Cat PE=1 SV=3 | Cat | 56.5 | 59.756 | 323.31 |
| A0A0G2K059 | Mitochondrial calcium uniporter OS=Rattus norvegicus OX=10116 GN=Mcu PE=1 SV=1 | Mcu | 35.8 | 36.057 | 128.09 |
| Q4KM87 | Actin-like 6A OS=Rattus norvegicus OX=10116 GN=Actl6a PE=1 SV=1 | Actl6a | 38.7 | 47.42 | 128.51 |
| D4A0T0 | NADH:ubiquinone oxidoreductase subunit B10 OS=Rattus norvegicus OX=10116 GN=Ndufb10 PE=1 SV=1 | Ndufb10 | 72.7 | 20.858 | 38.901 |
| P05708 | Hexokinase-1 OS=Rattus norvegicus OX=10116 GN=Hk1 PE=1 SV=4 | Hk1 | 40.5 | 102.41 | 292.68 |
| A0A0G2JZA2 | GrpE protein homolog OS=Rattus norvegicus OX=10116 GN=Grpel1 PE=1 SV=1 | Grpel1 | 49.6 | 25.811 | 84.858 |
| Q6IMZ3 | Annexin OS=Rattus norvegicus OX=10116 GN=Anxa6 PE=1 SV=1 | Anxa6 | 50.8 | 75.755 | 194.55 |
| Q9Z2L0 | Voltage-dependent anion-selective channel protein 1 OS=Rattus norvegicus OX=10116 GN=Vdac1 PE=1 SV=4 | Vdac1 | 73.9 | 30.755 | 323.31 |
| G3V7R8 | rRNA adenine N(6)-methyltransferase OS=Rattus norvegicus OX=10116 GN=Dimt1 PE=1 SV=1 | Dimt1 | 25.9 | 35.202 | 16.123 |
| Q09073 | ADP/ATP translocase 2 OS=Rattus norvegicus OX=10116 GN=Slc25a5 PE=1 SV=3 | Slc25a5 | 59.4 | 32.901 | 150.88 |
| Q66HG9 | Mitochondrial antiviral-signaling protein OS=Rattus norvegicus OX=10116 GN=Mavs PE=1 SV=1 | Mavs | 32.9 | 53.804 | 18.597 |
| P81155 | Voltage-dependent anion-selective channel protein 2 OS=Rattus norvegicus OX=10116 GN=Vdac2 PE=1 SV=2 | Vdac2 | 62 | 31.745 | 323.31 |
| Q04462 | Valine--tRNA ligase OS=Rattus norvegicus OX=10116 GN=Vars PE=2 SV=2 | Vars | 49.1 | 140.37 | 310.15 |
| P27881 | Hexokinase-2 OS=Rattus norvegicus OX=10116 GN=Hk2 PE=1 SV=1 | Hk2 | 47.5 | 102.54 | 160.9 |
| A0A0G2JVP1 | Vaccinia-related kinase 2 OS=Rattus norvegicus OX=10116 GN=Vrk2 PE=4 SV=1 | Vrk2 | 41.9 | 57.752 | 50.504 |
| G3V6H5 | Mitochondrial 2-oxoglutarate/malate carrier protein OS=Rattus norvegicus OX=10116 GN=Slc25a11 PE=1 SV=1 | Slc25a11 | 39.2 | 34.136 | 299.54 |
| P70550 | Ras-related protein Rab-8B OS=Rattus norvegicus OX=10116 GN=Rab8b PE=1 SV=1 | Rab8b | 25.1 | 23.603 | 69.508 |
| Q5FWY5 | AH receptor-interacting protein OS=Rattus norvegicus OX=10116 GN=Aip PE=1 SV=1 | Aip | 37 | 37.598 | 12.719 |
| F1M6D0 | Mitochondrial ribosomal protein S6 OS=Rattus norvegicus OX=10116 GN=Mrps6 PE=1 SV=2 | Mrps6 | 19.2 | 14.341 | 2.8708 |
| Q5EGY4 | Synaptobrevin homolog YKT6 OS=Rattus norvegicus OX=10116 GN=Ykt6 PE=1 SV=1 | Ykt6 | 29.8 | 22.369 | 6.34 |
| P18163 | Long-chain-fatty-acid--CoA ligase 1 OS=Rattus norvegicus OX=10116 GN=Acsl1 PE=1 SV=1 | Acsl1 | 25 | 78.178 | 34.595 |
| P63170 | "Dynein light chain 1, cytoplasmic OS=Rattus norvegicus OX=10116 GN=Dynll1 PE=1 SV=1" | Dynll1 | 65.2 | 10.366 | 146.93 |
| P62909 | 40S ribosomal protein S3 OS=Rattus norvegicus OX=10116 GN=Rps3 PE=1 SV=1 | Rps3 | 72 | 26.674 | 241.83 |
| A0A0G2JSR0 | Voltage-dependent anion-selective channel protein 3 OS=Rattus norvegicus OX=10116 GN=Vdac3 PE=1 SV=1 | Vdac3 | 47.7 | 30.783 | 304.59 |
| P63088 | Serine/threonine-protein phosphatase PP1-gamma catalytic subunit OS=Rattus norvegicus OX=10116 GN=Ppp1cc PE=1 SV=1 | Ppp1cc | 62.2 | 36.983 | 110.92 |
| D4A4P3 | NADH:ubiquinone oxidoreductase subunit B3 OS=Rattus norvegicus OX=10116 GN=Ndufb3 PE=1 SV=1 | Ndufb3 | 28.3 | 11.267 | 2.148 |
| A0A0G2JZ68 | rRNA adenine N(6)-methyltransferase OS=Rattus norvegicus OX=10116 GN=Tfb1m PE=1 SV=1 | Tfb1m | 23.5 | 39.097 | 6.0886 |
| O88767 | Protein/nucleic acid deglycase DJ-1 OS=Rattus norvegicus OX=10116 GN=Park7 PE=1 SV=1 | Park7 | 66.7 | 19.974 | 322.83 |
| Q9Z1X1 | Extended synaptotagmin-1 OS=Rattus norvegicus OX=10116 GN=Esyt1 PE=1 SV=1 | Esyt1 | 36 | 121.16 | 96.1 |
| Q6DGG0 | Peptidyl-prolyl cis-trans isomerase D OS=Rattus norvegicus OX=10116 GN=Ppid PE=1 SV=3 | Ppid | 29.5 | 40.765 | 16.215 |
| Q5XII9 | Mitochondrial fission regulator 1-like OS=Rattus norvegicus OX=10116 GN=Mtfr1l PE=1 SV=1 | Mtfr1l | 31.8 | 31.73 | 82.049 |
| Q5U1W6 | MICOS complex subunit OS=Rattus norvegicus OX=10116 GN=Apool PE=1 SV=1 | Apool | 52.4 | 28.237 | 29.523 |
| Q63151 | Long-chain-fatty-acid--CoA ligase 3 OS=Rattus norvegicus OX=10116 GN=Acsl3 PE=1 SV=1 | Acsl3 | 46.4 | 80.457 | 118.37 |
| Q5XI04 | "RCG45489, isoform CRA_a OS=Rattus norvegicus OX=10116 GN=Stom PE=1 SV=1" | Stom | 50.4 | 31.378 | 302.43 |
| D3ZD80 | General transcription factor IIIC subunit 4 OS=Rattus norvegicus OX=10116 GN=Gtf3c4 PE=1 SV=1 | Gtf3c4 | 23.8 | 91.713 | 24.474 |
| Q3KRE0 | ATPase family AAA domain-containing protein 3 OS=Rattus norvegicus OX=10116 GN=Atad3 PE=1 SV=1 | Atad3 | 37.4 | 66.758 | 64.137 |
| P07632 | Superoxide dismutase [Cu-Zn] OS=Rattus norvegicus OX=10116 GN=Sod1 PE=1 SV=2 | Sod1 | 45.5 | 15.911 | 144.88 |
| Q9ESH6 | Glutaredoxin-1 OS=Rattus norvegicus OX=10116 GN=Glrx PE=3 SV=3 | Glrx | 50.5 | 11.879 | 15.854 |
| P31399 | "ATP synthase subunit d, mitochondrial OS=Rattus norvegicus OX=10116 GN=Atp5pd PE=1 SV=3" | Atp5pd | 77 | 18.763 | 302.82 |
| Q6IN39 | Hsd17b4 protein OS=Rattus norvegicus OX=10116 GN=Hsd17b4 PE=1 SV=1 | Hsd17b4 | 42.9 | 81.088 | 254.98 |
| B1WBY5 | "DnaJ (Hsp40) homolog, subfamily C, member 11 OS=Rattus norvegicus OX=10116 GN=Dnajc11 PE=1 SV=1" | Dnajc11 | 27 | 63.204 | 26.106 |
| Q9JKW1 | Mitochondrial import inner membrane translocase subunit Tim22 OS=Rattus norvegicus OX=10116 GN=Timm22 PE=2 SV=2 | Timm22 | 23.4 | 19.918 | 14.269 |
| D3ZXF9 | Mitochondrial ribosomal protein L12 OS=Rattus norvegicus OX=10116 GN=Mrpl12 PE=1 SV=1 | Mrpl12 | 33 | 29.441 | 69.811 |
| D3ZD23 | ATP-binding cassette subfamily E member 1 OS=Rattus norvegicus OX=10116 GN=Abce1 PE=1 SV=1 | Abce1 | 53.9 | 67.3 | 323.31 |
| Q9WUD9 | Proto-oncogene tyrosine-protein kinase Src OS=Rattus norvegicus OX=10116 GN=Src PE=1 SV=4 | Src | 38.6 | 59.972 | 153.02 |
| P21913 | "Succinate dehydrogenase [ubiquinone] iron-sulfur subunit, mitochondrial OS=Rattus norvegicus OX=10116 GN=Sdhb PE=2 SV=2" | Sdhb | 40.8 | 31.83 | 144.02 |
| Q99M64 | Phosphatidylinositol 4-kinase type 2-alpha OS=Rattus norvegicus OX=10116 GN=Pi4k2a PE=1 SV=1 | Pi4k2a | 46.9 | 54.304 | 68.126 |
| A0A0G2JUZ5 | Glycine cleavage system P protein OS=Rattus norvegicus OX=10116 GN=Gldc PE=1 SV=1 | Gldc | 28.1 | 105.18 | 64.012 |
| P32089 | "Tricarboxylate transport protein, mitochondrial OS=Rattus norvegicus OX=10116 GN=Slc25a1 PE=1 SV=1" | Slc25a1 | 20.9 | 33.835 | 37.388 |
| F1LNF7 | "Isocitrate dehydrogenase [NAD] subunit, mitochondrial OS=Rattus norvegicus OX=10116 GN=Idh3a PE=1 SV=2" | Idh3a | 45.5 | 41.176 | 323.31 |
| Q05982 | Nucleoside diphosphate kinase A OS=Rattus norvegicus OX=10116 GN=Nme1 PE=1 SV=1 | Nme1 | 74.3 | 17.193 | 18.458 |
| Q6AXT0 | "39S ribosomal protein L37, mitochondrial OS=Rattus norvegicus OX=10116 GN=Mrpl37 PE=2 SV=1" | Mrpl37 | 44.9 | 48.365 | 40.148 |
| Q6AYQ8 | "Acylpyruvase FAHD1, mitochondrial OS=Rattus norvegicus OX=10116 GN=Fahd1 PE=1 SV=1" | Fahd1 | 14.9 | 24.48 | 4.9805 |
| D3ZJ32 | Extended synaptotagmin 2 OS=Rattus norvegicus OX=10116 GN=Esyt2 PE=1 SV=3 | Esyt2 | 37.9 | 93.95 | 74.6 |
| F1LZW6 | Solute carrier family 25 member 13 OS=Rattus norvegicus OX=10116 GN=Slc25a13 PE=1 SV=2 | Slc25a13 | 54.1 | 54.099 | 323.31 |
| Q9JJW3 | Up-regulated during skeletal muscle growth protein 5 OS=Rattus norvegicus OX=10116 GN=Atp5md PE=1 SV=1 | Atp5md | 44.8 | 6.4075 | 229.42 |
| M0RB91 | Queuine tRNA-ribosyltransferase accessory subunit 2 OS=Rattus norvegicus OX=10116 GN=Qtrt2 PE=1 SV=1 | Qtrt2 | 8 | 46.365 | 3.8636 |
| Q63965 | Sideroflexin-1 OS=Rattus norvegicus OX=10116 GN=Sfxn1 PE=2 SV=4 | Sfxn1 | 34.5 | 35.546 | 19.402 |
| A6N6J5 | WD repeat-containing protein 35 OS=Rattus norvegicus OX=10116 GN=Wdr35 PE=1 SV=1 | Wdr35 | 2.7 | 132.78 | 2.1113 |
| Q9R1R4 | Tudor domain-containing protein 7 OS=Rattus norvegicus OX=10116 GN=Tdrd7 PE=1 SV=1 | Tdrd7 | 17.9 | 125.31 | 24.455 |
| P63102 | 14-3-3 protein zeta/delta OS=Rattus norvegicus OX=10116 GN=Ywhaz PE=1 SV=1 | Ywhaz | 66.9 | 27.771 | 323.31 |
| D3ZAF6 | "ATP synthase subunit f, mitochondrial OS=Rattus norvegicus OX=10116 GN=Atp5mf PE=1 SV=1" | Atp5mf | 34.1 | 10.452 | 4.8032 |
| P24155 | Thimet oligopeptidase OS=Rattus norvegicus OX=10116 GN=Thop1 PE=1 SV=4 | Thop1 | 25.9 | 78.385 | 27.967 |
| Q9JLT6 | BH3-interacting domain death agonist OS=Rattus norvegicus OX=10116 GN=Bid PE=1 SV=2 | Bid | 26 | 22.249 | 23.539 |
| Q641Y5 | Ubiquitin-like modifier-activating enzyme ATG7 OS=Rattus norvegicus OX=10116 GN=Atg7 PE=2 SV=1 | Atg7 | 11 | 77.435 | 8.881 |
| A0A0G2K038 | "Oligoribonuclease, mitochondrial OS=Rattus norvegicus OX=10116 GN=Rexo2 PE=1 SV=1" | Rexo2 | 11.8 | 28.721 | 8.0373 |
| B0BN68 | Mitochondrial ribosomal protein S9 OS=Rattus norvegicus OX=10116 GN=Mrps9 PE=1 SV=1 | Mrps9 | 28.2 | 45.01 | 23.779 |
| Q4QR80 | "28S ribosomal protein S25, mitochondrial OS=Rattus norvegicus OX=10116 GN=Mrps25 PE=2 SV=1" | Mrps25 | 48.5 | 19.775 | 11.16 |
| P82995 | Heat shock protein HSP 90-alpha OS=Rattus norvegicus OX=10116 GN=Hsp90aa1 PE=1 SV=3 | Hsp90aa1 | 51.6 | 84.814 | 323.31 |
| Q9JHY2 | Sideroflexin-3 OS=Rattus norvegicus OX=10116 GN=Sfxn3 PE=2 SV=1 | Sfxn3 | 43 | 35.433 | 38.022 |
| D4A830 | Pyrophosphatase (inorganic) 2 OS=Rattus norvegicus OX=10116 GN=Ppa2 PE=1 SV=2 | Ppa2 | 35.5 | 35.046 | 31.19 |
| D3ZKT0 | "Phosphatidate cytidylyltransferase, mitochondrial OS=Rattus norvegicus OX=10116 GN=Tamm41 PE=1 SV=1" | Tamm41 | 36.8 | 37.785 | 54.854 |
| A0A0G2JSG6 | "Adenylate kinase 2, mitochondrial OS=Rattus norvegicus OX=10116 GN=Ak2 PE=1 SV=1" | Ak2 | 58.2 | 25.529 | 72.943 |
| Q5HZA9 | Transmembrane protein 126A OS=Rattus norvegicus OX=10116 GN=Tmem126a PE=2 SV=1 | Tmem126a | 28.1 | 21.657 | 16.395 |
| D3ZUJ5 | Deoxythymidylate kinase OS=Rattus norvegicus OX=10116 GN=Dtymk PE=1 SV=1 | Dtymk | 17.9 | 23.973 | 5.8507 |
| Q68FU3 | Electron transfer flavoprotein subunit beta OS=Rattus norvegicus OX=10116 GN=Etfb PE=1 SV=3 | Etfb | 30.6 | 27.687 | 121.19 |
| A0A0H2UHV7 | "Alanine--tRNA ligase, mitochondrial OS=Rattus norvegicus OX=10116 GN=Aars2 PE=1 SV=1" | Aars2 | 19.3 | 99.264 | 32.242 |
| Q56A33 | Mitogen-activated protein kinase OS=Rattus norvegicus OX=10116 GN=Mapk14 PE=1 SV=1 | Mapk14 | 17.2 | 41.473 | 5.0317 |
| Q66HI5 | Ferritin OS=Rattus norvegicus OX=10116 GN=Fth1 PE=1 SV=2 | Fth1 | 54.4 | 21.099 | 23.886 |
| Q4QQV4 | Dead end homolog 1 (Zebrafish) OS=Rattus norvegicus OX=10116 GN=Hars PE=1 SV=1 | Hars | 27.2 | 57.36 | 55.338 |
| P84817 | Mitochondrial fission 1 protein OS=Rattus norvegicus OX=10116 GN=Fis1 PE=1 SV=1 | Fis1 | 33.6 | 16.994 | 96.465 |
| Q7TS56 | Carbonyl reductase family member 4 OS=Rattus norvegicus OX=10116 GN=Cbr4 PE=2 SV=1 | Cbr4 | 35.6 | 25.286 | 11.426 |
| Q8VHI8 | Vesicle transport protein SEC20 OS=Rattus norvegicus OX=10116 GN=Bnip1 PE=2 SV=1 | Bnip1 | 33.3 | 26.17 | 19.145 |
| F1LR53 | Kidney mitochondrial carrier protein 1 OS=Rattus norvegicus OX=10116 GN=Slc25a30 PE=3 SV=3 | Slc25a30 | 9.7 | 32.305 | 4.0644 |
| P32198 | "Carnitine O-palmitoyltransferase 1, liver isoform OS=Rattus norvegicus OX=10116 GN=Cpt1a PE=1 SV=2" | Cpt1a | 15.7 | 88.124 | 9.9936 |
| Q63362 | NADH dehydrogenase [ubiquinone] 1 alpha subcomplex subunit 5 OS=Rattus norvegicus OX=10116 GN=Ndufa5 PE=1 SV=3 | Ndufa5 | 62.1 | 13.412 | 87.359 |
| D3ZBM3 | Ferrochelatase OS=Rattus norvegicus OX=10116 GN=Fech PE=1 SV=1 | Fech | 28 | 47.551 | 13.554 |
| D4AEG2 | "RAB32, member RAS oncogene family OS=Rattus norvegicus OX=10116 GN=Rab32 PE=1 SV=1" | Rab32 | 40.4 | 25.26 | 12.063 |
| G3V8F5 | Mitochondrial import receptor subunit TOM40 homolog OS=Rattus norvegicus OX=10116 GN=Tomm40 PE=1 SV=1 | Tomm40 | 56.2 | 37.92 | 323.31 |
| Q9WVJ4 | Synaptojanin-2-binding protein OS=Rattus norvegicus OX=10116 GN=Synj2bp PE=1 SV=2 | Synj2bp | 17.9 | 15.825 | 3.1724 |
| D3ZYU4 | COX20 cytochrome C oxidase assembly factor OS=Rattus norvegicus OX=10116 GN=Cox20 PE=1 SV=1 | Cox20 | 11.1 | 13.078 | 1.9892 |
| A0A0G2JYU2 | Mitochondrial ribosomal protein L11 OS=Rattus norvegicus OX=10116 GN=mrpl11 PE=1 SV=1 | mrpl11 | 25 | 20.75 | 5.3397 |
| P45352 | Thymidylate synthase OS=Rattus norvegicus OX=10116 GN=Tyms PE=1 SV=1 | Tyms | 26.1 | 35.017 | 5.0796 |
| Q9QVC8 | Peptidyl-prolyl cis-trans isomerase FKBP4 OS=Rattus norvegicus OX=10116 GN=Fkbp4 PE=1 SV=3 | Fkbp4 | 45.2 | 51.45 | 82.236 |
| Q5XIW0 | Mitochondrial import inner membrane translocase subunit TIM23 OS=Rattus norvegicus OX=10116 GN=LOC100362432 PE=1 SV=1 | LOC100362432 | 53.1 | 21.873 | 28.191 |
| P63031 | Mitochondrial pyruvate carrier 1 OS=Rattus norvegicus OX=10116 GN=Mpc1 PE=3 SV=1 | Mpc1 | 43.1 | 12.454 | 24.256 |
| Q5M7W1 | Thioredoxin-interacting protein OS=Rattus norvegicus OX=10116 GN=Txnip PE=2 SV=1 | Txnip | 24.1 | 44.017 | 19.157 |
| Q920D2 | Dihydrofolate reductase OS=Rattus norvegicus OX=10116 GN=Dhfr PE=1 SV=3 | Dhfr | 31 | 21.638 | 6.5725 |
| Q6AXV4 | Sorting and assembly machinery component 50 homolog OS=Rattus norvegicus OX=10116 GN=Samm50 PE=1 SV=1 | Samm50 | 43.1 | 51.96 | 235.27 |
| P70473 | Alpha-methylacyl-CoA racemase OS=Rattus norvegicus OX=10116 GN=Amacr PE=1 SV=3 | Amacr | 16.5 | 41.828 | 5.4446 |
| A0A0A0MXW1 | "2-oxoisovalerate dehydrogenase subunit beta, mitochondrial OS=Rattus norvegicus OX=10116 GN=Bckdhb PE=1 SV=1" | Bckdhb | 23 | 42.96 | 22.582 |
| P67779 | Prohibitin OS=Rattus norvegicus OX=10116 GN=Phb PE=1 SV=1 |  | 72.8 | 29.82 | 323.31 |
| A0A0G2K350 | NSF attachment protein gamma OS=Rattus norvegicus OX=10116 GN=Napg PE=1 SV=1 | Napg | 27.2 | 34.659 | 11.057 |
| P24473 | Glutathione S-transferase kappa 1 OS=Rattus norvegicus OX=10116 GN=Gstk1 PE=1 SV=3 | Gstk1 | 19.9 | 25.493 | 36.432 |
| F1LN46 | "Carnitine O-palmitoyltransferase 1, brain isoform OS=Rattus norvegicus OX=10116 GN=Cpt1c PE=1 SV=1" | Cpt1c | 5.9 | 90.169 | 2.5005 |
| Q6AZ50 | Ubiquitin-like-conjugating enzyme ATG3 OS=Rattus norvegicus OX=10116 GN=Atg3 PE=2 SV=1 | Atg3 | 28 | 35.822 | 4.1278 |
| P29419 | "ATP synthase subunit e, mitochondrial OS=Rattus norvegicus OX=10116 GN=Atp5me PE=1 SV=3" | Atp5me | 39.4 | 8.2545 | 6.6849 |
| P47196 | RAC-alpha serine/threonine-protein kinase OS=Rattus norvegicus OX=10116 GN=Akt1 PE=1 SV=1 | Akt1 | 9.2 | 55.735 | 2.8797 |
| Q6MG60 | "N(G),N(G)-dimethylarginine dimethylaminohydrolase 2 OS=Rattus norvegicus OX=10116 GN=Ddah2 PE=1 SV=1" | Ddah2 | 55.4 | 29.688 | 17.68 |
| F1LP30 | "Methylcrotonoyl-CoA carboxylase subunit alpha, mitochondrial OS=Rattus norvegicus OX=10116 GN=Mccc1 PE=1 SV=1" | Mccc1 | 13.7 | 79.295 | 6.9146 |
| A0A096MKB0 | Max dimerization protein 3 OS=Rattus norvegicus OX=10116 GN=Rab24 PE=1 SV=1 | Rab24 | 34 | 23.144 | 10.452 |
| D3ZY44 | Mitochondrial ribosomal protein S2 OS=Rattus norvegicus OX=10116 GN=Mrps2 PE=1 SV=1 | Mrps2 | 38.1 | 32.175 | 66.32 |
| P19804 | Nucleoside diphosphate kinase B OS=Rattus norvegicus OX=10116 GN=Nme2 PE=1 SV=1 | Nme2 | 69.1 | 17.283 | 323.31 |
| O08697 | ADP-ribosylation factor-like protein 2 OS=Rattus norvegicus OX=10116 GN=Arl2 PE=1 SV=1 | Arl2 | 16.3 | 20.836 | 2.8706 |
| D3ZXI0 | Pyrroline-5-carboxylate reductase OS=Rattus norvegicus OX=10116 GN=Pycr1 PE=3 SV=2 | Pycr1 | 47.6 | 37.316 | 52.391 |
| G3V8A5 | Vacuolar protein sorting-associated protein 35 OS=Rattus norvegicus OX=10116 GN=Vps35 PE=1 SV=1 | Vps35 | 45.2 | 91.726 | 323.31 |
| Q6MG61 | Chloride intracellular channel protein 1 OS=Rattus norvegicus OX=10116 GN=Clic1 PE=1 SV=1 | Clic1 | 71.4 | 26.98 | 81.715 |
| Q5U3Z3 | Isochorismatase domain-containing protein 2 OS=Rattus norvegicus OX=10116 GN=Isoc2 PE=2 SV=1 | Isoc2 | 27.6 | 23.157 | 2.9662 |
| D3ZUX5 | MICOS complex subunit OS=Rattus norvegicus OX=10116 GN=Chchd3 PE=1 SV=1 | Chchd3 | 35.7 | 26.434 | 72.733 |
| P07150 | Annexin A1 OS=Rattus norvegicus OX=10116 GN=Anxa1 PE=1 SV=2 | Anxa1 | 61.3 | 38.829 | 323.31 |
| B5DEL8 | NADH dehydrogenase (Ubiquinone) Fe-S protein 5 OS=Rattus norvegicus OX=10116 GN=Ndufs5 PE=1 SV=1 | Ndufs5 | 37.7 | 12.7 | 7.4361 |
| G3V879 | "5-demethoxyubiquinone hydroxylase, mitochondrial OS=Rattus norvegicus OX=10116 GN=Coq7 PE=3 SV=4" | Coq7 | 53.1 | 20.126 | 14.331 |
| Q00981 | Ubiquitin carboxyl-terminal hydrolase isozyme L1 OS=Rattus norvegicus OX=10116 GN=Uchl1 PE=1 SV=2 | Uchl1 | 48.9 | 24.838 | 35.138 |
| Q6P2A5 | "GTP:AMP phosphotransferase AK3, mitochondrial OS=Rattus norvegicus OX=10116 GN=Ak3 PE=1 SV=1" | Ak3 | 37.4 | 25.494 | 15.736 |
| Q6AXQ0 | SUMO-activating enzyme subunit 1 OS=Rattus norvegicus OX=10116 GN=Sae1 PE=2 SV=1 | Sae1 | 33 | 38.512 | 10.93 |
| A0A0G2K777 | Ras homolog family member T1 OS=Rattus norvegicus OX=10116 GN=Rhot1 PE=1 SV=1 | Rhot1 | 19.1 | 86.063 | 25.241 |
| P10688 | "1-phosphatidylinositol 4,5-bisphosphate phosphodiesterase delta-1 OS=Rattus norvegicus OX=10116 GN=Plcd1 PE=1 SV=1" | Plcd1 | 18.7 | 85.961 | 18.7 |
| Q5XIM4 | "ATP synthase subunit s, mitochondrial OS=Rattus norvegicus OX=10116 GN=Atp5s PE=2 SV=1" | Atp5s | 19 | 23.324 | 3.2258 |
| Q3MIE4 | Synaptic vesicle membrane protein VAT-1 homolog OS=Rattus norvegicus OX=10116 GN=Vat1 PE=1 SV=1 | Vat1 | 67.1 | 43.118 | 223.62 |
| O35547 | Long-chain-fatty-acid--CoA ligase 4 OS=Rattus norvegicus OX=10116 GN=Acsl4 PE=1 SV=1 | Acsl4 | 59 | 74.326 | 258.15 |
| D4A9T5 | "EF-hand domain family, member D1 OS=Rattus norvegicus OX=10116 GN=Efhd1 PE=4 SV=1" | Efhd1 | 59.6 | 27.055 | 101.07 |
| Q9QZ81 | Protein argonaute-2 OS=Rattus norvegicus OX=10116 GN=Ago2 PE=2 SV=2 | Ago2 | 39.9 | 97.317 | 60.275 |
| Q3KRD5 | Mitochondrial import receptor subunit TOM34 OS=Rattus norvegicus OX=10116 GN=Tomm34 PE=1 SV=1 | Tomm34 | 53.4 | 34.461 | 113.95 |
| D3ZZ21 | "NADH dehydrogenase (Ubiquinone) 1 beta subcomplex, 6 (Predicted) OS=Rattus norvegicus OX=10116 GN=Ndufb6 PE=1 SV=1" | Ndufb6 | 34.4 | 15.638 | 7.9336 |
| Q3B8P4 | "Decapping enzyme, scavenger OS=Rattus norvegicus OX=10116 GN=Dcps PE=1 SV=1" | Dcps | 24.2 | 38.593 | 5.5732 |
| A0A0H2UI42 | "39S ribosomal protein L30, mitochondrial OS=Rattus norvegicus OX=10116 GN=Mrpl30 PE=1 SV=1" | Mrpl30 | 10.6 | 20.714 | 6.1507 |
| B2GV53 | Slc25a32 protein OS=Rattus norvegicus OX=10116 GN=Slc25a32 PE=1 SV=1 | Slc25a32 | 12.3 | 35.045 | 4.2164 |
| B2RZD2 | "Complex I assembly factor TMEM126B, mitochondrial OS=Rattus norvegicus OX=10116 GN=Tmem126b PE=1 SV=2" | Tmem126b | 17.5 | 25.213 | 2.5315 |
| D3ZAQ0 | FUN14 domain-containing 2 OS=Rattus norvegicus OX=10116 GN=Fundc2 PE=1 SV=1 | Fundc2 | 20.5 | 16.344 | 3.8603 |
| D3ZCM4 | "DnaJ (Hsp40) homolog, subfamily C, member 15 (Predicted), isoform CRA_a OS=Rattus norvegicus OX=10116 GN=Dnajc15 PE=1 SV=1" | Dnajc15 | 12.1 | 15.991 | 2.6907 |
| D3ZEJ2 | Mitochondrial calcium uniporter regulator 1 OS=Rattus norvegicus OX=10116 GN=Mcur1 PE=1 SV=1 | Mcur1 | 3.6 | 37.551 | 1.5165 |
| D3ZY50 | ATP synthase mitochondrial F1 complex assembly factor 1 OS=Rattus norvegicus OX=10116 GN=Atpaf1 PE=1 SV=1 | Atpaf1 | 5.7 | 38.649 | 2.2831 |
| M0RAK2 | RCG22622 OS=Rattus norvegicus OX=10116 GN=LOC684270 PE=1 SV=2 | LOC684270 | 12.9 | 22.929 | 16.055 |
| M9VYP0 | BCL2-interacting protein 3 OS=Rattus norvegicus OX=10116 GN=Bnip3 PE=1 SV=1 | Bnip3 | 14.7 | 8.236 | 1.7189 |
| P21575 | Dynamin-1 OS=Rattus norvegicus OX=10116 GN=Dnm1 PE=1 SV=2 | Dnm1 | 6.9 | 97.294 | 1.7765 |
| P55063 | Heat shock 70 kDa protein 1-like OS=Rattus norvegicus OX=10116 GN=Hspa1l PE=2 SV=2 | Hspa1l | 11.9 | 70.548 | 2.3444 |
| P62716 | Serine/threonine-protein phosphatase 2A catalytic subunit beta isoform OS=Rattus norvegicus OX=10116 GN=Ppp2cb PE=2 SV=1 | Ppp2cb | 60.8 | 35.575 | -2 |
| P97564 | "Glycerol-3-phosphate acyltransferase 1, mitochondrial OS=Rattus norvegicus OX=10116 GN=Gpam PE=1 SV=3" | Gpam | 4.8 | 93.713 | 2.763 |
| Q08877 | Dynamin-3 OS=Rattus norvegicus OX=10116 GN=Dnm3 PE=1 SV=2 | Dnm3 | 9.6 | 97.913 | 7.2957 |
| Q32WR5 | Biogenesis of lysosome-related organelles complex-1 subunit 2 OS=Rattus norvegicus OX=10116 GN=Bloc1s2 PE=2 SV=1 | Bloc1s2 | 12 | 16.067 | 3.1976 |
| Q4V8B7 | Inactive hydroxysteroid dehydrogenase-like protein 1 OS=Rattus norvegicus OX=10116 GN=Hsdl1 PE=2 SV=1 | Hsdl1 | 10 | 36.907 | 4.561 |
| Q52KK3 | Solute carrier family 25 member 51 OS=Rattus norvegicus OX=10116 GN=Slc25a51 PE=2 SV=1 | Slc25a51 | 6.4 | 33.774 | 10.004 |
| Q5BJS4 | FUN14 domain-containing protein 1 OS=Rattus norvegicus OX=10116 GN=Fundc1 PE=1 SV=1 | Fundc1 | 11 | 17.158 | 2.0479 |
| Q5BJX1 | "39S ribosomal protein L41, mitochondrial OS=Rattus norvegicus OX=10116 GN=Mrpl41 PE=1 SV=1" | Mrpl41 | 45.5 | 15.192 | 3.8709 |
| Q5RJP0 | Aldose reductase-related protein 1 OS=Rattus norvegicus OX=10116 GN=Akr1b7 PE=1 SV=1 | Akr1b7 | 16.1 | 36.121 | 1.9819 |
| Q63538 | Mitogen-activated protein kinase 12 OS=Rattus norvegicus OX=10116 GN=Mapk12 PE=1 SV=1 | Mapk12 | 6.3 | 41.985 | 1.5309 |
| Q6AYS2 | Sideroflexin OS=Rattus norvegicus OX=10116 GN=Sfxn1 PE=1 SV=1 | Sfxn1 | 32.3 | 35.588 | 1.9686 |
| Q8K3P6 | Calcium-binding mitochondrial carrier protein SCaMC-2 OS=Rattus norvegicus OX=10116 GN=Slc25a25 PE=1 SV=1 | Slc25a25 | 11.5 | 52.694 | 3.9101 |
| Q8VH49 | "HIG1 domain family member 1A, mitochondrial OS=Rattus norvegicus OX=10116 GN=Higd1a PE=1 SV=2" | Higd1a | 43 | 10.301 | 49.724 |
| Q91XJ1 | Beclin-1 OS=Rattus norvegicus OX=10116 GN=Becn1 PE=1 SV=1 | Becn1 | 9.6 | 51.556 | 2.5811 |
| Q920G4 | Caspase 9 OS=Rattus norvegicus OX=10116 GN=Casp9 PE=1 SV=1 | Casp9 | 20.9 | 19.7 | 2.2636 |
| Q6AYL0 | "RCG35015, isoform CRA_a OS=Rattus norvegicus OX=10116 GN=Slc25a19 PE=2 SV=1" | Slc25a19 | 5 | 35.638 | 3.7107 |
| F1LQ55 | Non-specific lipid-transfer protein OS=Rattus norvegicus OX=10116 GN=Scp2 PE=1 SV=3 | Scp2 | 10.8 | 58.787 | 12.633 |
| Q9ER24 | Ataxin-10 OS=Rattus norvegicus OX=10116 GN=Atxn10 PE=1 SV=1 | Atxn10 | 58.9 | 53.726 | 200.83 |
| A0A096MJM1 | Ras homolog family member G (Fragment) OS=Rattus norvegicus OX=10116 GN=Rhog PE=1 SV=6 | Rhog | 70.8 | 20.622 | 180.1 |
| D4A4V1 | Mitochondrial ribosomal protein S18C OS=Rattus norvegicus OX=10116 GN=Mrps18c PE=4 SV=1 | Mrps18c | 13.3 | 16.371 | 7.8539 |
| P12785 | Fatty acid synthase OS=Rattus norvegicus OX=10116 GN=Fasn PE=1 SV=3 | Fasn | 50.6 | 272.65 | 323.31 |
| P63245 | Receptor of activated protein C kinase 1 OS=Rattus norvegicus OX=10116 GN=Rack1 PE=1 SV=3 | Rack1 | 88 | 35.076 | 323.31 |
| A1A5Q1 | Poly [ADP-ribose] polymerase OS=Rattus norvegicus OX=10116 GN=Parp9 PE=1 SV=1 | Parp9 | 29.9 | 92.214 | 43.785 |
| B0BNJ1 | LOC683667 protein OS=Rattus norvegicus OX=10116 GN=Sri PE=1 SV=1 | Sri | 36.4 | 21.624 | 24.628 |
| P47942 | Dihydropyrimidinase-related protein 2 OS=Rattus norvegicus OX=10116 GN=Dpysl2 PE=1 SV=1 | Dpysl2 | 57.9 | 62.277 | 323.31 |
| F1LPG5 | NADH:ubiquinone oxidoreductase subunit B4 OS=Rattus norvegicus OX=10116 GN=Ndufb4 PE=1 SV=1 | Ndufb4 | 46.5 | 15.064 | 16.389 |
| Q91VC0 | dCTP pyrophosphatase 1 OS=Rattus norvegicus OX=10116 GN=Dctpp1 PE=2 SV=1 | Dctpp1 | 24.7 | 18.473 | 4.4055 |
| A0A0G2KAM3 | "Pyruvate dehydrogenase E1 component subunit beta, mitochondrial OS=Rattus norvegicus OX=10116 GN=Pdhb PE=1 SV=1" | Pdhb | 55.1 | 46.192 | 323.31 |
| P61078 | Ubiquitin-conjugating enzyme E2 D3 OS=Rattus norvegicus OX=10116 GN=Ube2d3 PE=2 SV=1 | Ube2d3 | 23.1 | 16.687 | 2.949 |
| D3ZCA0 | Pyridoxal phosphate homeostasis protein OS=Rattus norvegicus OX=10116 GN=Plpbp PE=1 SV=1 | Plpbp | 16.4 | 30.104 | 5.2041 |
| Q7TQ16 | Cytochrome b-c1 complex subunit 8 OS=Rattus norvegicus OX=10116 GN=Uqcrq PE=3 SV=1 | Uqcrq | 26.8 | 9.8492 | 10.22 |
| P52631 | Signal transducer and activator of transcription 3 OS=Rattus norvegicus OX=10116 GN=Stat3 PE=1 SV=1 | Stat3 | 56.1 | 88.039 | 323.31 |
| Q5XI26 | Signal transducer and activator of transcription OS=Rattus norvegicus OX=10116 GN=Stat2 PE=1 SV=1 | Stat2 | 11.9 | 96.856 | 8.1782 |
| P46462 | Transitional endoplasmic reticulum ATPase OS=Rattus norvegicus OX=10116 GN=Vcp PE=1 SV=3 | Vcp | 70.8 | 89.348 | 323.31 |
| E9PT22 | "Inverted formin, FH2 and WH2 domain containing OS=Rattus norvegicus OX=10116 GN=Inf2 PE=1 SV=3" | Inf2 | 24.1 | 136.46 | 86.927 |
| P12369 | cAMP-dependent protein kinase type II-beta regulatory subunit OS=Rattus norvegicus OX=10116 GN=Prkar2b PE=1 SV=3 | Prkar2b | 36.3 | 46.122 | 20.731 |
| M0RDR1 | "Enoyl-CoA hydratase domain-containing protein 3, mitochondrial (Fragment) OS=Rattus norvegicus OX=10116 GN=Echdc3 PE=1 SV=3" | Echdc3 | 16.2 | 21.966 | 1.6526 |
| O35509 | Ras-related protein Rab-11B OS=Rattus norvegicus OX=10116 GN=Rab11b PE=1 SV=4 | Rab11b | 43.1 | 24.488 | 323.31 |
| Q6TXG7 | Serine hydroxymethyltransferase OS=Rattus norvegicus OX=10116 GN=Shmt1 PE=1 SV=1 | Shmt1 | 16.4 | 75.373 | 8.216 |
| P63012 | Ras-related protein Rab-3A OS=Rattus norvegicus OX=10116 GN=Rab3a PE=1 SV=1 | Rab3a | 12.7 | 24.97 | 2.0777 |
| Q63942 | GTP-binding protein Rab-3D OS=Rattus norvegicus OX=10116 GN=Rab3d PE=1 SV=2 | Rab3d | 6.8 | 24.29 | 1.8165 |
| G3V9Z3 | Amine oxidase OS=Rattus norvegicus OX=10116 GN=Maoa PE=1 SV=1 | Maoa | 34.4 | 59.52 | 323.31 |
| P06761 | Endoplasmic reticulum chaperone BiP OS=Rattus norvegicus OX=10116 GN=Hspa5 PE=1 SV=1 | Hspa5 | 52.3 | 72.346 | 323.31 |
| O88813 | Long-chain-fatty-acid--CoA ligase 5 OS=Rattus norvegicus OX=10116 GN=Acsl5 PE=1 SV=1 | Acsl5 | 34.3 | 76.404 | 83.228 |
| P35565 | Calnexin OS=Rattus norvegicus OX=10116 GN=Canx PE=1 SV=1 | Canx | 52.8 | 67.254 | 323.31 |
| Q32Q06 | AP-1 complex subunit mu-1 OS=Rattus norvegicus OX=10116 GN=Ap1m1 PE=1 SV=3 | Ap1m1 | 33.1 | 48.556 | 22.055 |
| F1LXF5 | Golgi to ER traffic protein 4 OS=Rattus norvegicus OX=10116 GN=Get4 PE=1 SV=2 | Get4 | 21.2 | 36.752 | 12.361 |
| A0A0G2K3K1 | Ubiquitin carboxyl-terminal hydrolase 30 OS=Rattus norvegicus OX=10116 GN=Usp30 PE=1 SV=1 | Usp30 | 9.5 | 54.386 | 1.7764 |
| Q6AY94 | "Complex I assembly factor TIMMDC1, mitochondrial OS=Rattus norvegicus OX=10116 GN=Timmdc1 PE=2 SV=1" | Timmdc1 | 9.8 | 32.05 | 12.897 |
| Q6AXY8 | Dehydrogenase/reductase (SDR family) member 1 OS=Rattus norvegicus OX=10116 GN=Dhrs1 PE=1 SV=1 | Dhrs1 | 38.7 | 34.015 | 25.017 |
| B2RYT0 | Mitochondrial ribosomal protein S21 OS=Rattus norvegicus OX=10116 GN=Mrps21 PE=2 SV=1 | Mrps21 | 29.9 | 10.6 | 2.6349 |
| F1LP21 | Translocase of inner mitochondrial membrane 8A1 OS=Rattus norvegicus OX=10116 GN=Timm8a1 PE=1 SV=3 | Timm8a1 | 69.5 | 10.888 | 120.83 |
| D3ZD09 | Cytochrome c oxidase subunit OS=Rattus norvegicus OX=10116 GN=Cox6b1 PE=1 SV=1 | Cox6b1 | 41.9 | 10.071 | 9.2793 |
| Q3B7U9 | Peptidyl-prolyl cis-trans isomerase FKBP8 OS=Rattus norvegicus OX=10116 GN=Fkbp8 PE=2 SV=1 | Fkbp8 | 25.1 | 43.555 | 194.85 |
| D4A305 | "Coiled-coil domain containing 58 (Predicted), isoform CRA_c OS=Rattus norvegicus OX=10116 GN=Ccdc58 PE=1 SV=1" | Ccdc58 | 52.8 | 16.69 | 24.836 |
| G3V7Z3 | Nucleolar protein 3 OS=Rattus norvegicus OX=10116 GN=Nol3 PE=1 SV=1 | Nol3 | 48 | 24.491 | 24.184 |
| D3ZAE6 | RCG49849 OS=Rattus norvegicus OX=10116 GN=Vasn PE=1 SV=1 | Vasn | 12.3 | 72.32 | 12.004 |
| G3V9N1 | RCG21137 OS=Rattus norvegicus OX=10116 GN=Pgam5 PE=1 SV=1 | Pgam5 | 51.4 | 32 | 323.31 |
| D3ZZV1 | Presequence translocase-associated motor 16 OS=Rattus norvegicus OX=10116 GN=Pam16 PE=1 SV=1 | Pam16 | 46.4 | 13.771 | 139.19 |
| Q5U1Z9 | Metaxin 2 OS=Rattus norvegicus OX=10116 GN=Mtx2 PE=1 SV=1 | Mtx2 | 51 | 29.718 | 224.08 |
| D3ZM21 | "Catechol-O-methyltransferase domain containing 1 (Predicted), isoform CRA_a OS=Rattus norvegicus OX=10116 GN=Comtd1 PE=1 SV=1" | Comtd1 | 12.6 | 28.987 | 8.2371 |
| P62074 | Mitochondrial import inner membrane translocase subunit Tim10 OS=Rattus norvegicus OX=10116 GN=Timm10 PE=3 SV=1 | Timm10 | 40 | 10.333 | 40.389 |
| Q6QBQ4 | Phospholipid scramblase 3 OS=Rattus norvegicus OX=10116 GN=Plscr3 PE=2 SV=1 | Plscr3 | 28 | 31.646 | 194.11 |
| Q6IN22 | Cathepsin B OS=Rattus norvegicus OX=10116 GN=Ctsb PE=1 SV=1 | Ctsb | 48.1 | 37.544 | 323.31 |
| Q9WV97 | Mitochondrial import inner membrane translocase subunit Tim9 OS=Rattus norvegicus OX=10116 GN=Timm9 PE=1 SV=3 | Timm9 | 47.2 | 10.376 | 41.193 |
| Q6AXX6 | Redox-regulatory protein FAM213A OS=Rattus norvegicus OX=10116 GN=Fam213a PE=1 SV=1 | Fam213a | 10.5 | 25.763 | 2.3032 |
| B2RZD6 | "NDUFA4, mitochondrial complex-associated OS=Rattus norvegicus OX=10116 GN=Ndufa4 PE=1 SV=1" | Ndufa4 | 37.8 | 9.3267 | 9.467 |
| P62076 | Mitochondrial import inner membrane translocase subunit Tim13 OS=Rattus norvegicus OX=10116 GN=Timm13 PE=3 SV=1 | Timm13 | 78.9 | 10.458 | 120.92 |
| D4ACN9 | Solute carrier family 25 member 36 OS=Rattus norvegicus OX=10116 GN=Slc25a36 PE=3 SV=1 | Slc25a36 | 15.1 | 34.266 | 2.312 |
| P22734 | Catechol O-methyltransferase OS=Rattus norvegicus OX=10116 GN=Comt PE=1 SV=2 | Comt | 49.6 | 29.597 | 21.981 |
| P28494 | Alpha-mannosidase 2 OS=Rattus norvegicus OX=10116 GN=Man2a1 PE=1 SV=2 | Man2a1 | 35.8 | 131.24 | 107.62 |
| P54001 | Prolyl 4-hydroxylase subunit alpha-1 OS=Rattus norvegicus OX=10116 GN=P4ha1 PE=2 SV=2 | P4ha1 | 60.5 | 60.897 | 323.31 |
| Q6AY23 | Pyrroline-5-carboxylate reductase 2 OS=Rattus norvegicus OX=10116 GN=Pycr2 PE=2 SV=1 | Pycr2 | 54.4 | 33.673 | 161.54 |
| P62078 | Mitochondrial import inner membrane translocase subunit Tim8 B OS=Rattus norvegicus OX=10116 GN=Timm8b PE=3 SV=1 | Timm8b | 49.4 | 9.2864 | 7.9636 |
| D3ZUZ4 | "FAM20B, glycosaminoglycan xylosylkinase OS=Rattus norvegicus OX=10116 GN=Fam20b PE=1 SV=1" | Fam20b | 25.4 | 46.568 | 7.4778 |
| Q499R4 | "YrdC domain-containing protein, mitochondrial OS=Rattus norvegicus OX=10116 GN=Yrdc PE=2 SV=1" | Yrdc | 16.4 | 29.23 | 2.5122 |
| A0A0G2JVL6 | NADH dehydrogenase [ubiquinone] 1 alpha subcomplex subunit 8 OS=Rattus norvegicus OX=10116 GN=Ndufa8 PE=1 SV=1 | Ndufa8 | 54.7 | 19.965 | 83.709 |
| Q642E6 | Tripeptidyl peptidase I OS=Rattus norvegicus OX=10116 GN=Tpp1 PE=1 SV=1 | Tpp1 | 12.3 | 61.312 | 60.377 |
| P05371 | Clusterin OS=Rattus norvegicus OX=10116 GN=Clu PE=1 SV=2 | Clu | 13 | 51.375 | 5.0085 |
| D3ZTW9 | "Endonuclease G-like 1 (Predicted), isoform CRA_d OS=Rattus norvegicus OX=10116 GN=Exog PE=1 SV=1" | Exog | 26.4 | 41.326 | 8.1961 |
| Q62760 | Mitochondrial import receptor subunit TOM20 homolog OS=Rattus norvegicus OX=10116 GN=Tomm20 PE=1 SV=2 | Tomm20 | 47.6 | 16.284 | 31.818 |
| E9PT90 | Spartin OS=Rattus norvegicus OX=10116 GN=Spart PE=1 SV=3 | Spart | 13.8 | 85.865 | 14.213 |
| D4A471 | COX assembly mitochondrial protein OS=Rattus norvegicus OX=10116 GN=Cmc2 PE=1 SV=1 | Cmc2 | 40.5 | 9.4086 | 4.4992 |
| Q7M0E7 | "39S ribosomal protein L14, mitochondrial OS=Rattus norvegicus OX=10116 GN=Mrpl14 PE=1 SV=2" | Mrpl14 | 31.7 | 15.912 | 4.2061 |
| B0K017 | ADP-ribosylhydrolase like 2 (Predicted) OS=Rattus norvegicus OX=10116 GN=Adprhl2 PE=1 SV=1 | Adprhl2 | 11.1 | 39.375 | 2.4597 |
| O88884 | "A-kinase anchor protein 1, mitochondrial OS=Rattus norvegicus OX=10116 GN=Akap1 PE=1 SV=1" | Akap1 | 18 | 91.746 | 12.261 |
| O89035 | Mitochondrial dicarboxylate carrier OS=Rattus norvegicus OX=10116 GN=Slc25a10 PE=1 SV=1 | Slc25a10 | 38.8 | 31.454 | 146.22 |
| D3ZFR9 | Retinol dehydrogenase 13 OS=Rattus norvegicus OX=10116 GN=Rdh13 PE=1 SV=1 | Rdh13 | 21.3 | 36.407 | 6.4386 |
| Q5XID1 | Anamorsin OS=Rattus norvegicus OX=10116 GN=Ciapin1 PE=1 SV=1 | Ciapin1 | 17.5 | 33.041 | 176.91 |
| D4A5W8 | CDP-diacylglycerol--glycerol-3-phosphate 3-phosphatidyltransferase OS=Rattus norvegicus OX=10116 GN=Pgs1 PE=3 SV=2 | Pgs1 | 13.7 | 57.755 | 7.9062 |
| P11951 | Cytochrome c oxidase subunit 6C-2 OS=Rattus norvegicus OX=10116 GN=Cox6c2 PE=1 SV=3 | Cox6c2 | 31.6 | 8.4548 | 196.92 |
| P26453 | Basigin OS=Rattus norvegicus OX=10116 GN=Bsg PE=1 SV=2 | Bsg | 23.5 | 42.435 | 197.43 |
| Q5FVC5 | Nectin cell adhesion molecule 2 OS=Rattus norvegicus OX=10116 GN=Nectin2 PE=1 SV=1 | Nectin2 | 31.1 | 57.323 | 22.422 |
| Q6IN37 | GM2 ganglioside activator OS=Rattus norvegicus OX=10116 GN=Gm2a PE=1 SV=1 | Gm2a | 40.7 | 21.493 | 73.264 |
| P50430 | Arylsulfatase B OS=Rattus norvegicus OX=10116 GN=Arsb PE=2 SV=2 | Arsb | 12.7 | 58.958 | 7.1065 |
| A0A0G2K7P7 | Mitochondrial carrier 2 OS=Rattus norvegicus OX=10116 GN=Mtch2 PE=1 SV=1 | Mtch2 | 55.1 | 26.998 | 152.13 |
| D4AEG6 | Mitochondrial ribosomal protein L32 OS=Rattus norvegicus OX=10116 GN=Mrpl32 PE=1 SV=1 | Mrpl32 | 29.4 | 21.601 | 8.6488 |
| D3ZLT1 | "NADH dehydrogenase (Ubiquinone) 1 beta subcomplex, 7 (Predicted) OS=Rattus norvegicus OX=10116 GN=Ndufb7 PE=1 SV=1" | Ndufb7 | 18.2 | 16.568 | 3.2498 |
| Q5XI64 | Monoacylglycerol lipase ABHD6 OS=Rattus norvegicus OX=10116 GN=Abhd6 PE=1 SV=1 | Abhd6 | 27.6 | 38.311 | 9.4968 |
| Q675A5 | Group XV phospholipase A2 OS=Rattus norvegicus OX=10116 GN=Pla2g15 PE=1 SV=1 | Pla2g15 | 16.2 | 47.391 | 7.64 |
| Q6AXM8 | Serum paraoxonase/arylesterase 2 OS=Rattus norvegicus OX=10116 GN=Pon2 PE=2 SV=1 | Pon2 | 32.2 | 39.617 | 78.34 |
| Q6AY58 | B-cell receptor-associated protein 31 OS=Rattus norvegicus OX=10116 GN=Bcap31 PE=1 SV=1 | Bcap31 | 26.1 | 27.911 | 43.537 |
| Q5M9I5 | "Cytochrome b-c1 complex subunit 6, mitochondrial OS=Rattus norvegicus OX=10116 GN=Uqcrh PE=3 SV=1" | Uqcrh | 29.2 | 10.424 | 106.07 |
| Q5XI86 | Peptidyl-tRNA hydrolase 2 OS=Rattus norvegicus OX=10116 GN=Ptrh2 PE=1 SV=1 | Ptrh2 | 56.4 | 19.529 | 70.527 |
| Q5U3Z5 | BRI3-binding protein OS=Rattus norvegicus OX=10116 GN=Bri3bp PE=1 SV=1 | Bri3bp | 16.6 | 28.316 | 18.092 |
| B1WBW4 | Armadillo repeat-containing protein 10 OS=Rattus norvegicus OX=10116 GN=Armc10 PE=1 SV=1 | Armc10 | 36.6 | 33.421 | 94.481 |
| Q80W89 | NADH dehydrogenase [ubiquinone] 1 alpha subcomplex subunit 11 OS=Rattus norvegicus OX=10116 GN=Ndufa11 PE=2 SV=1 | Ndufa11 | 60.3 | 14.854 | 19.564 |
| B0BMY5 | Chchd5 protein OS=Rattus norvegicus OX=10116 GN=Chchd5 PE=1 SV=1 | Chchd5 | 42.7 | 12.323 | 6.8629 |
| A0A0G2JSS8 | "Peroxiredoxin 5, isoform CRA_c OS=Rattus norvegicus OX=10116 GN=Prdx5 PE=1 SV=1" | Prdx5 | 55.9 | 22.206 | 323.31 |
| Q9R1B1 | Mitochondrial import inner membrane translocase subunit Tim10 B OS=Rattus norvegicus OX=10116 GN=Timm10b PE=3 SV=1 | Timm10b | 44 | 11.351 | 13.473 |
| B0K020 | CDGSH iron-sulfur domain-containing protein 1 OS=Rattus norvegicus OX=10116 GN=Cisd1 PE=3 SV=1 | Cisd1 | 50 | 12.097 | 296.59 |
| Q5FVL2 | ER membrane protein complex subunit 8 OS=Rattus norvegicus OX=10116 GN=Emc8 PE=2 SV=1 | Emc8 | 43 | 23.405 | 175.43 |
| D3ZUY0 | Retinol dehydrogenase 14 OS=Rattus norvegicus OX=10116 GN=Rdh14 PE=1 SV=1 | Rdh14 | 27.8 | 36.195 | 13.708 |
| A0A0G2JVG4 | Peroxisomal trans-2-enoyl-CoA reductase OS=Rattus norvegicus OX=10116 GN=Pecr PE=1 SV=1 | Pecr | 21 | 32.737 | 5.3181 |
| D3ZTN2 | COX assembly mitochondrial protein OS=Rattus norvegicus OX=10116 GN=Cmc1 PE=1 SV=2 | Cmc1 | 39.6 | 12.572 | 3.8944 |
| D3ZSM9 | BRCA1-associated ATM activator 1 OS=Rattus norvegicus OX=10116 GN=Brat1 PE=1 SV=3 | Brat1 | 8.2 | 88.475 | 7.528 |
| Q5BJN5 | Mitochondrial intermembrane space import and assembly protein 40 OS=Rattus norvegicus OX=10116 GN=Chchd4 PE=2 SV=1 | Chchd4 | 36.7 | 15.467 | 48.799 |
| D4AC65 | Cytochrome c oxidase assembly factor 7 OS=Rattus norvegicus OX=10116 GN=Coa7 PE=1 SV=1 | Coa7 | 13.4 | 25.711 | 3.0715 |
| A0A0G2JSH5 | Serum albumin OS=Rattus norvegicus OX=10116 GN=Alb PE=1 SV=1 | Alb | 4.6 | 68.758 | 1.6247 |
| A0A0G2KAE4 | Sulfhydryl oxidase OS=Rattus norvegicus OX=10116 GN=LOC100912596 PE=4 SV=1 | LOC100912596 | 23.6 | 16.828 | 1.7816 |
| A0A0H2UHF4 | RCG62278 OS=Rattus norvegicus OX=10116 GN=Rsad2 PE=4 SV=1 | Rsad2 | 7.2 | 41.443 | 1.5825 |
| B0K013 | Chchd8 protein OS=Rattus norvegicus OX=10116 GN=Coa4 PE=2 SV=1 | Coa4 | 37.9 | 10.208 | 2.1246 |
| D3Z9Z2 | "Putative lipoyltransferase 2, mitochondrial OS=Rattus norvegicus OX=10116 GN=Lipt2 PE=3 SV=1" | Lipt2 | 24.2 | 25.197 | 4.3794 |
| D3ZLK9 | ATP-dependent (S)-NAD(P)H-hydrate dehydratase OS=Rattus norvegicus OX=10116 GN=Naxd PE=1 SV=2 | Naxd | 11.8 | 38.73 | 3.7321 |
| D4A650 | Peptide-methionine (R)-S-oxide reductase OS=Rattus norvegicus OX=10116 GN=Msrb3 PE=1 SV=3 | Msrb3 | 16.6 | 29.033 | 5.5236 |
| F7EPE0 | Prosaposin OS=Rattus norvegicus OX=10116 GN=Psap PE=1 SV=2 | Psap | 35.4 | 61.453 | 4.8959 |
| M0R7T2 | AlkB homolog 7 OS=Rattus norvegicus OX=10116 GN=Alkbh7 PE=1 SV=1 | Alkbh7 | 18.6 | 25.016 | 3.1355 |
| P08011 | Microsomal glutathione S-transferase 1 OS=Rattus norvegicus OX=10116 GN=Mgst1 PE=1 SV=3 | Mgst1 | 16.1 | 17.471 | 2.0928 |
| Q9EQL9 | Sharpin OS=Rattus norvegicus OX=10116 GN=Sharpin PE=1 SV=1 | Sharpin | 4.7 | 40.286 | 1.8409 |
| Q66H15 | Regulator of microtubule dynamics protein 3 OS=Rattus norvegicus OX=10116 GN=Rmdn3 PE=1 SV=1 | Rmdn3 | 28 | 52.311 | 42.736 |
| P35571 | "Glycerol-3-phosphate dehydrogenase, mitochondrial OS=Rattus norvegicus OX=10116 GN=Gpd2 PE=1 SV=1" | Gpd2 | 63.1 | 80.972 | 323.31 |
| F1LQT9 | DNA (cytosine-5)-methyltransferase OS=Rattus norvegicus OX=10116 GN=Dnmt1 PE=1 SV=3 | Dnmt1 | 32.1 | 183.04 | 151.3 |
| A0A0G2K9E5 | "Leucyl-tRNA synthetase 2, mitochondrial OS=Rattus norvegicus OX=10116 GN=Lars2 PE=1 SV=1" | Lars2 | 14.1 | 101.58 | 9.6571 |
| D4ADB4 | CGG triplet repeat binding protein 1 (Predicted) OS=Rattus norvegicus OX=10116 GN=Cggbp1 PE=1 SV=1 | Cggbp1 | 38.9 | 18.82 | 53.949 |
| Q5PPH0 | Enolase-phosphatase E1 OS=Rattus norvegicus OX=10116 GN=Enoph1 PE=2 SV=1 | Enoph1 | 29.1 | 28.874 | 10.785 |
| Q9Z272 | ARF GTPase-activating protein GIT1 OS=Rattus norvegicus OX=10116 GN=Git1 PE=1 SV=1 | Git1 | 29 | 85.23 | 35.296 |
| Q6P9Y4 | ADP/ATP translocase 1 OS=Rattus norvegicus OX=10116 GN=Slc25a4 PE=1 SV=1 | Slc25a4 | 43.6 | 32.904 | 27.101 |
| A0A140TAB4 | Core histone macro-H2A OS=Rattus norvegicus OX=10116 GN=H2afy PE=1 SV=1 | H2afy | 41.3 | 39.07 | 323.31 |
| A0A0G2JUN8 | Proteasome subunit beta type OS=Rattus norvegicus OX=10116 GN=Psmb7 PE=1 SV=1 | Psmb7 | 49.1 | 31.807 | 139 |
| P69736 | Endothelial differentiation-related factor 1 OS=Rattus norvegicus OX=10116 GN=Edf1 PE=1 SV=1 | Edf1 | 31.1 | 16.369 | 26.556 |
| P29266 | "3-hydroxyisobutyrate dehydrogenase, mitochondrial OS=Rattus norvegicus OX=10116 GN=Hibadh PE=1 SV=3" | Hibadh | 45.1 | 35.302 | 79.731 |
| P13233 | "2',3'-cyclic-nucleotide 3'-phosphodiesterase OS=Rattus norvegicus OX=10116 GN=Cnp PE=1 SV=2" | Cnp | 49.8 | 47.268 | 202.76 |
| F1M9V7 | Aminopeptidase OS=Rattus norvegicus OX=10116 GN=Npepps PE=1 SV=1 | Npepps | 37.8 | 103.34 | 239.39 |
| Q5XIN6 | Mitochondrial proton/calcium exchanger protein OS=Rattus norvegicus OX=10116 GN=Letm1 PE=1 SV=1 | Letm1 | 45.1 | 83.059 | 180.37 |
| Q66H80 | Coatomer subunit delta OS=Rattus norvegicus OX=10116 GN=Arcn1 PE=2 SV=1 | Arcn1 | 46.8 | 57.199 | 212 |
| G3V7B7 | DNA-directed RNA polymerase subunit OS=Rattus norvegicus OX=10116 GN=Polr1a PE=1 SV=1 | Polr1a | 19.8 | 194.09 | 38.885 |
| M0RDD7 | Chromatin target of PRMT1-like 1 OS=Rattus norvegicus OX=10116 GN=Chtopl1 PE=4 SV=1 | Chtopl1 | 36 | 22.136 | 11.816 |
| B2GUZ6 | Reticulon 4-interacting protein 1 OS=Rattus norvegicus OX=10116 GN=Rtn4ip1 PE=1 SV=1 | Rtn4ip1 | 22.7 | 43.605 | 8.6449 |
| B2RYS8 | "NADH dehydrogenase [ubiquinone] 1 beta subcomplex subunit 8, mitochondrial OS=Rattus norvegicus OX=10116 GN=Ndufb8 PE=1 SV=1" | Ndufb8 | 26.3 | 21.959 | 9.1257 |
| D3ZF13 | Acyl carrier protein OS=Rattus norvegicus OX=10116 GN=Ndufab1 PE=1 SV=1 | Ndufab1 | 23.7 | 17.514 | 10.57 |
| B2RYS9 | "Similar to RIKEN cDNA 0610038D11 (Predicted), isoform CRA_b OS=Rattus norvegicus OX=10116 GN=Trmt112 PE=1 SV=1" | Trmt112 | 47.2 | 14.127 | 14.957 |
| Q91XS8 | Serine/threonine-protein kinase 17B OS=Rattus norvegicus OX=10116 GN=Stk17b PE=1 SV=1 | Stk17b | 7.8 | 42.132 | 3.8584 |
| E9PT51 | DNA polymerase delta-interacting protein 2 OS=Rattus norvegicus OX=10116 GN=Poldip2 PE=1 SV=3 | Poldip2 | 50.3 | 41.797 | 117.13 |
| P13803 | "Electron transfer flavoprotein subunit alpha, mitochondrial OS=Rattus norvegicus OX=10116 GN=Etfa PE=1 SV=4" | Etfa | 64 | 34.951 | 323.31 |
| F7EY92 | "Methyl-CpG binding domain protein 3 (Predicted), isoform CRA_c OS=Rattus norvegicus OX=10116 GN=Mbd3 PE=1 SV=2" | Mbd3 | 53.1 | 28.467 | 20.995 |
| P10860 | "Glutamate dehydrogenase 1, mitochondrial OS=Rattus norvegicus OX=10116 GN=Glud1 PE=1 SV=2" | Glud1 | 58.2 | 61.415 | 323.31 |
| Q75Q41 | Mitochondrial import receptor subunit TOM22 homolog OS=Rattus norvegicus OX=10116 GN=Tomm22 PE=1 SV=1 | Tomm22 | 66.2 | 15.49 | 323.31 |
| Q569C9 | Golgi phosphoprotein 3 OS=Rattus norvegicus OX=10116 GN=Golph3 PE=1 SV=1 | Golph3 | 36.2 | 33.752 | 10.548 |
| G3V640 | Mitochondrial import inner membrane translocase subunit TIM44 OS=Rattus norvegicus OX=10116 GN=Timm44 PE=1 SV=1 | Timm44 | 34 | 51.06 | 114.93 |
| P85845 | Fascin OS=Rattus norvegicus OX=10116 GN=Fscn1 PE=1 SV=2 | Fscn1 | 65.1 | 54.49 | 323.31 |
| A0A0G2JVH4 | MICOS complex subunit MIC60 OS=Rattus norvegicus OX=10116 GN=Immt PE=1 SV=1 | Immt | 48.7 | 86.229 | 323.31 |
| Q4V897 | "Coiled-coil domain-containing protein 90B, mitochondrial OS=Rattus norvegicus OX=10116 GN=Ccdc90b PE=2 SV=1" | Ccdc90b | 32.4 | 29.8 | 17.639 |
| D3Z8B2 | Nucleoporin 133 OS=Rattus norvegicus OX=10116 GN=Nup133 PE=1 SV=3 | Nup133 | 35.3 | 128.36 | 85.666 |
| A1L1M0 | "Protein kinase, cAMP-dependent, catalytic, alpha OS=Rattus norvegicus OX=10116 GN=Prkaca PE=1 SV=1" | Prkaca | 45 | 40.605 | 56.959 |
| D4A2P1 | Cell division cycle and apoptosis regulator 1 OS=Rattus norvegicus OX=10116 GN=Ccar1 PE=1 SV=2 | Ccar1 | 25.4 | 132.15 | 88.461 |
| F1LSW7 | 60S ribosomal protein L14 OS=Rattus norvegicus OX=10116 GN=Rpl14 PE=1 SV=2 | Rpl14 | 22.9 | 23.322 | 24.212 |
| Q5M818 | "39S ribosomal protein L16, mitochondrial OS=Rattus norvegicus OX=10116 GN=Mrpl16 PE=2 SV=1" | Mrpl16 | 11.2 | 28.895 | 3.8251 |
| A0A0G2JV31 | "X-prolyl aminopeptidase (Aminopeptidase P) 1, soluble, isoform CRA_a OS=Rattus norvegicus OX=10116 GN=Xpnpep1 PE=1 SV=1" | Xpnpep1 | 35.3 | 74.667 | 31.045 |
| G3V936 | Citrate synthase OS=Rattus norvegicus OX=10116 GN=Cs PE=1 SV=1 | Cs | 40.8 | 51.83 | 323.31 |
| P97615 | "Thioredoxin, mitochondrial OS=Rattus norvegicus OX=10116 GN=Txn2 PE=2 SV=1" | Txn2 | 37.3 | 18.232 | 16.409 |
| G3V9W6 | Aldehyde dehydrogenase OS=Rattus norvegicus OX=10116 GN=Aldh3a2 PE=1 SV=1 | Aldh3a2 | 42.8 | 54.107 | 44.081 |
| P00507 | "Aspartate aminotransferase, mitochondrial OS=Rattus norvegicus OX=10116 GN=Got2 PE=1 SV=2" | Got2 | 70.5 | 47.314 | 323.31 |
| P04636 | "Malate dehydrogenase, mitochondrial OS=Rattus norvegicus OX=10116 GN=Mdh2 PE=1 SV=2" | Mdh2 | 52.4 | 35.683 | 323.31 |
| D3Z941 | Methionyl-tRNA synthetase OS=Rattus norvegicus OX=10116 GN=Mars PE=1 SV=1 | Mars | 52.4 | 101.58 | 163.03 |
| F1M953 | "Stress-70 protein, mitochondrial OS=Rattus norvegicus OX=10116 GN=Hspa9 PE=1 SV=1" | Hspa9 | 58.3 | 73.744 | 323.31 |
| P26772 | "10 kDa heat shock protein, mitochondrial OS=Rattus norvegicus OX=10116 GN=Hspe1 PE=1 SV=3" | Hspe1 | 52.9 | 10.902 | 100.1 |
| E9PTV0 | Guanylate kinase 1 OS=Rattus norvegicus OX=10116 GN=Guk1 PE=1 SV=2 | Guk1 | 26.5 | 24.052 | 7.1771 |
| P38718 | Mitochondrial pyruvate carrier 2 OS=Rattus norvegicus OX=10116 GN=Mpc2 PE=2 SV=1 | Mpc2 | 54.3 | 14.258 | 12.994 |
| P54319 | Phospholipase A-2-activating protein OS=Rattus norvegicus OX=10116 GN=Plaa PE=1 SV=3 | Plaa | 38.1 | 87.083 | 129.72 |
| Q5PQX0 | UDP-glucuronic acid decarboxylase 1 OS=Rattus norvegicus OX=10116 GN=Uxs1 PE=1 SV=1 | Uxs1 | 33.6 | 47.538 | 30.6 |
| Q6IMX3 | "Acetyl-Coenzyme A dehydrogenase, short chain, isoform CRA_a OS=Rattus norvegicus OX=10116 GN=Acads PE=1 SV=1" | Acads | 43.7 | 44.967 | 68.131 |
| P20788 | "Cytochrome b-c1 complex subunit Rieske, mitochondrial OS=Rattus norvegicus OX=10116 GN=Uqcrfs1 PE=1 SV=2" | Uqcrfs1 | 40.9 | 29.445 | 136.36 |
| Q91ZW1 | "Transcription factor A, mitochondrial OS=Rattus norvegicus OX=10116 GN=Tfam PE=2 SV=1" | Tfam | 19.7 | 28.186 | 29.837 |
| F1LVX1 | DnaJ heat shock protein family (Hsp40) member C1 OS=Rattus norvegicus OX=10116 GN=Dnajc1 PE=1 SV=3 | Dnajc1 | 15.4 | 65.36 | 11.093 |
| F1LQI1 | Hydroxyacyl glutathione hydrolase OS=Rattus norvegicus OX=10116 GN=Hagh PE=1 SV=2 | Hagh | 27.8 | 34.158 | 10.852 |
| P97532 | 3-mercaptopyruvate sulfurtransferase OS=Rattus norvegicus OX=10116 GN=Mpst PE=1 SV=3 | Mpst | 33 | 32.94 | 43.992 |
| P23965 | "Enoyl-CoA delta isomerase 1, mitochondrial OS=Rattus norvegicus OX=10116 GN=Eci1 PE=1 SV=1" | Eci1 | 48.1 | 32.254 | 196.58 |
| Q68FX0 | "Isocitrate dehydrogenase [NAD] subunit beta, mitochondrial OS=Rattus norvegicus OX=10116 GN=Idh3B PE=2 SV=1" | Idh3B | 54.8 | 42.353 | 323.31 |
| D3ZJH9 | Malic enzyme OS=Rattus norvegicus OX=10116 GN=Me2 PE=1 SV=1 | Me2 | 53.8 | 65.351 | 70.131 |
| Q5XIG8 | Serine-threonine kinase receptor-associated protein OS=Rattus norvegicus OX=10116 GN=Strap PE=1 SV=1 | Strap | 57.4 | 38.456 | 99.04 |
| P56571 | "ES1 protein homolog, mitochondrial OS=Rattus norvegicus OX=10116 PE=1 SV=2" | --- | 53.4 | 28.172 | 177.93 |
| D3ZM09 | Seryl-tRNA synthetase 2 (Predicted) OS=Rattus norvegicus OX=10116 GN=Sars2 PE=1 SV=1 | Sars2 | 19.1 | 58.286 | 11.287 |
| Q5XIJ3 | "Isocitrate dehydrogenase [NAD] subunit, mitochondrial OS=Rattus norvegicus OX=10116 GN=Idh3g PE=1 SV=1" | Idh3g | 46.3 | 42.85 | 316.43 |
| P19234 | "NADH dehydrogenase [ubiquinone] flavoprotein 2, mitochondrial OS=Rattus norvegicus OX=10116 GN=Ndufv2 PE=1 SV=2" | Ndufv2 | 35.1 | 27.378 | 42.802 |
| P14408 | "Fumarate hydratase, mitochondrial OS=Rattus norvegicus OX=10116 GN=Fh PE=1 SV=1" | Fh | 50.3 | 54.463 | 323.31 |
| B2RYV8 | Mitochondrial ribosomal protein L52 OS=Rattus norvegicus OX=10116 GN=Mrpl52 PE=1 SV=1 | Mrpl52 | 40.2 | 13.728 | 12.286 |
| A0A0G2K7K2 | "Apoptosis-inducing factor 1, mitochondrial OS=Rattus norvegicus OX=10116 GN=Aifm1 PE=1 SV=1" | Aifm1 | 43.9 | 66.133 | 132.27 |
| P08461 | "Dihydrolipoyllysine-residue acetyltransferase component of pyruvate dehydrogenase complex, mitochondrial OS=Rattus norvegicus OX=10116 GN=Dlat PE=1 SV=3" | Dlat | 33.1 | 67.165 | 92.816 |
| Q66HF3 | "Electron transfer flavoprotein-ubiquinone oxidoreductase, mitochondrial OS=Rattus norvegicus OX=10116 GN=Etfdh PE=1 SV=1" | Etfdh | 23.4 | 68.163 | 61.79 |
| Q5M7W7 | "Probable proline--tRNA ligase, mitochondrial OS=Rattus norvegicus OX=10116 GN=Pars2 PE=2 SV=1" | Pars2 | 26.3 | 53.332 | 14.461 |
| F1LM33 | "Leucine-rich PPR motif-containing protein, mitochondrial OS=Rattus norvegicus OX=10116 GN=Lrpprc PE=1 SV=2" | Lrpprc | 52.9 | 156.68 | 323.31 |
| D3ZYL4 | Mitochondrial ribosomal protein L50 OS=Rattus norvegicus OX=10116 GN=Mrpl50 PE=1 SV=1 | Mrpl50 | 24.5 | 18.319 | 6.7674 |
| G3V7G8 | Glycine--tRNA ligase OS=Rattus norvegicus OX=10116 GN=Gars PE=1 SV=1 | Gars | 37.4 | 81.792 | 28.882 |
| D4AA14 | "Apoptosis-inducing factor, mitochondria-associated 2 OS=Rattus norvegicus OX=10116 GN=Aifm2 PE=1 SV=1" | Aifm2 | 13.9 | 40.69 | 15.345 |
| Q9R1V3 | Mothers against decapentaplegic homolog 5 OS=Rattus norvegicus OX=10116 GN=Smad5 PE=1 SV=1 | Smad5 | 23.7 | 52.215 | 14.631 |
| D3ZHG3 | Protein tyrosine kinase 7 OS=Rattus norvegicus OX=10116 GN=Ptk7 PE=3 SV=3 | Ptk7 | 34.7 | 86.263 | 95.031 |
| P56522 | "NADPH:adrenodoxin oxidoreductase, mitochondrial OS=Rattus norvegicus OX=10116 GN=Fdxr PE=1 SV=1" | Fdxr | 32 | 54.362 | 26.776 |
| M0RAD5 | ATP-dependent Clp protease proteolytic subunit OS=Rattus norvegicus OX=10116 GN=Clpp PE=1 SV=1 | Clpp | 27.6 | 29.722 | 18.703 |
| G3V7Z1 | RCG47403 OS=Rattus norvegicus OX=10116 GN=Rcl1 PE=1 SV=3 | Rcl1 | 37 | 40.81 | 20.518 |
| P15999 | "ATP synthase subunit alpha, mitochondrial OS=Rattus norvegicus OX=10116 GN=Atp5f1a PE=1 SV=2" | Atp5f1a | 63.3 | 59.753 | 323.31 |
| F1LWG4 | "NADH dehydrogenase (Ubiquinone) 1 alpha subcomplex, assembly factor 1 (Predicted), isoform CRA_a OS=Rattus norvegicus OX=10116 GN=Ndufaf1 PE=1 SV=2" | Ndufaf1 | 25 | 37.781 | 5.7967 |
| Q5XIT9 | "Methylcrotonoyl-CoA carboxylase beta chain, mitochondrial OS=Rattus norvegicus OX=10116 GN=Mccc2 PE=2 SV=1" | Mccc2 | 38.9 | 61.516 | 12.97 |
| P61751 | ADP-ribosylation factor 4 OS=Rattus norvegicus OX=10116 GN=Arf4 PE=2 SV=2 | Arf4 | 71.1 | 20.396 | 172.76 |
| D3ZU04 | Uncharacterized protein OS=Rattus norvegicus OX=10116 PE=4 SV=1 | --- | 25.8 | 14.312 | 101.86 |
| Q2KN99 | Cytospin-A OS=Rattus norvegicus OX=10116 GN=Specc1l PE=1 SV=1 | Specc1l | 21.3 | 124.34 | 43.838 |
| A0A0G2K5E4 | DnaJ heat shock protein family (Hsp40) member A3 OS=Rattus norvegicus OX=10116 GN=Dnaja3 PE=1 SV=1 | Dnaja3 | 37.7 | 52.403 | 85.886 |
| F7EZZ0 | Death-associated protein 3 OS=Rattus norvegicus OX=10116 GN=Dap3 PE=1 SV=1 | Dap3 | 45.7 | 45.111 | 21.867 |
| D3ZV54 | "PWP2, small subunit processome component OS=Rattus norvegicus OX=10116 GN=Pwp2 PE=1 SV=1" | Pwp2 | 23.9 | 102.98 | 35.669 |
| P30277 | G2/mitotic-specific cyclin-B1 OS=Rattus norvegicus OX=10116 GN=Ccnb1 PE=2 SV=1 | Ccnb1 | 13.7 | 47.391 | 6.3009 |
| Q9WUS0 | "Adenylate kinase 4, mitochondrial OS=Rattus norvegicus OX=10116 GN=Ak4 PE=2 SV=1" | Ak4 | 54.3 | 25.203 | 20.905 |
| Q66HF1 | "NADH-ubiquinone oxidoreductase 75 kDa subunit, mitochondrial OS=Rattus norvegicus OX=10116 GN=Ndufs1 PE=1 SV=1" | Ndufs1 | 59.8 | 79.411 | 305.74 |
| Q5XIA5 | Coenzyme A synthase OS=Rattus norvegicus OX=10116 GN=Coasy PE=1 SV=1 | Coasy | 9.4 | 62.187 | 4.7192 |
| D3ZJX5 | "RCG54610, isoform CRA_a OS=Rattus norvegicus OX=10116 GN=Timm50 PE=1 SV=2" | Timm50 | 26.3 | 39.854 | 104.47 |
| P13264 | "Glutaminase kidney isoform, mitochondrial OS=Rattus norvegicus OX=10116 GN=Gls PE=1 SV=2" | Gls | 58.3 | 74.023 | 192.96 |
| Q00972 | "[3-methyl-2-oxobutanoate dehydrogenase [lipoamide]] kinase, mitochondrial OS=Rattus norvegicus OX=10116 GN=Bckdk PE=1 SV=2" | Bckdk | 29.4 | 46.474 | 97.693 |
| Q5U313 | Ankyrin repeat domain 13a OS=Rattus norvegicus OX=10116 GN=Ankrd13a PE=1 SV=1 | Ankrd13a | 19.2 | 67.319 | 12.083 |
| B2RZD1 | Protein transport protein Sec61 subunit beta OS=Rattus norvegicus OX=10116 GN=Sec61b PE=1 SV=1 | Sec61b | 26 | 9.9884 | 45.097 |
| F1LM55 | Cell cycle and apoptosis regulator 2 OS=Rattus norvegicus OX=10116 GN=Ccar2 PE=1 SV=1 | Ccar2 | 37.1 | 102.79 | 156.33 |
| Q9ER34 | "Aconitate hydratase, mitochondrial OS=Rattus norvegicus OX=10116 GN=Aco2 PE=1 SV=2" | Aco2 | 47.9 | 85.432 | 323.31 |
| P12075 | "Cytochrome c oxidase subunit 5B, mitochondrial OS=Rattus norvegicus OX=10116 GN=Cox5b PE=1 SV=2" | Cox5b | 34.1 | 13.915 | 40.699 |
| Q5BJT6 | Large subunit GTPase 1 homolog OS=Rattus norvegicus OX=10116 GN=Lsg1 PE=2 SV=1 | Lsg1 | 7.3 | 74.402 | 3.9345 |
| G3V8Z3 | G patch domain and KOW motifs OS=Rattus norvegicus OX=10116 GN=Gpkow PE=1 SV=1 | Gpkow | 12.5 | 53.259 | 5.1777 |
| D4AE56 | Prostaglandin E synthase 2 OS=Rattus norvegicus OX=10116 GN=Ptges2 PE=1 SV=1 | Ptges2 | 29.4 | 43.451 | 24.426 |
| A0A0G2JU15 | Pentatricopeptide repeat domain 3 OS=Rattus norvegicus OX=10116 GN=Ptcd3 PE=1 SV=1 | Ptcd3 | 29 | 84.556 | 38.82 |
| P07895 | "Superoxide dismutase [Mn], mitochondrial OS=Rattus norvegicus OX=10116 GN=Sod2 PE=1 SV=2" | Sod2 | 75.7 | 24.674 | 153.57 |
| P35435 | "ATP synthase subunit gamma, mitochondrial OS=Rattus norvegicus OX=10116 GN=Atp5f1c PE=1 SV=2" | Atp5f1c | 42.5 | 30.19 | 104.17 |
| P21670 | Proteasome subunit alpha type-4 OS=Rattus norvegicus OX=10116 GN=Psma4 PE=1 SV=1 | Psma4 | 43.3 | 29.497 | 14.376 |
| B2RZ72 | Actin-related protein 2/3 complex subunit 4 OS=Rattus norvegicus OX=10116 GN=Arpc4 PE=1 SV=1 | Arpc4 | 67.3 | 19.667 | 289.99 |
| G3V6G7 | Polyribonucleotide nucleotidyltransferase 1 OS=Rattus norvegicus OX=10116 GN=Pnpt1 PE=1 SV=1 | Pnpt1 | 25.6 | 85.897 | 33.529 |
| D4A197 | Methylmalonyl CoA epimerase OS=Rattus norvegicus OX=10116 GN=Mcee PE=1 SV=1 | Mcee | 34.8 | 18.816 | 31.199 |
| Q4V8F3 | Rhomboid-related protein 4 OS=Rattus norvegicus OX=10116 GN=Rhbdd1 PE=2 SV=1 | Rhbdd1 | 9.8 | 35.879 | 11.246 |
| Q3B8R7 | Mitochondrial ribosomal protein L47 OS=Rattus norvegicus OX=10116 GN=Mrpl47 PE=1 SV=1 | Mrpl47 | 33.7 | 29.616 | 6.8588 |
| D4A131 | Mitochondrial ribosomal protein L4 OS=Rattus norvegicus OX=10116 GN=Mrpl4 PE=1 SV=1 | Mrpl4 | 41.5 | 33.082 | 18.823 |
| F1LN92 | AFG3-like matrix AAA peptidase subunit 2 OS=Rattus norvegicus OX=10116 GN=Afg3l2 PE=1 SV=1 | Afg3l2 | 20.1 | 89.35 | 29.024 |
| D4A3S8 | "NOL1/NOP2/Sun domain family, member 2 (Predicted) OS=Rattus norvegicus OX=10116 GN=Nsun2 PE=1 SV=1" | Nsun2 | 37.5 | 88.09 | 31.965 |
| D4A9D8 | Oxysterol-binding protein OS=Rattus norvegicus OX=10116 GN=Osbp PE=1 SV=2 | Osbp | 27.4 | 77.806 | 26.794 |
| A0A0G2K1W9 | Lactate dehydrogenase D OS=Rattus norvegicus OX=10116 GN=Ldhd PE=1 SV=1 | Ldhd | 31.5 | 51.842 | 3.9073 |
| A0A0G2K849 | TATA-box binding protein associated factor 4 OS=Rattus norvegicus OX=10116 GN=Taf4 PE=1 SV=1 | Taf4 | 10.5 | 68.602 | 4.3942 |
| Q5U3Z7 | Serine hydroxymethyltransferase OS=Rattus norvegicus OX=10116 GN=Shmt2 PE=1 SV=1 | Shmt2 | 54 | 55.764 | 323.31 |
| D4A5F7 | VPS8 CORVET complex subunit OS=Rattus norvegicus OX=10116 GN=Vps8 PE=1 SV=3 | Vps8 | 5.3 | 146.52 | 4.9088 |
| D3ZEM0 | DNA-3-methyladenine glycosylase OS=Rattus norvegicus OX=10116 GN=Mpg PE=3 SV=1 | Mpg | 10 | 36.147 | 3.4339 |
| Q5RK08 | Glioblastoma amplified sequence OS=Rattus norvegicus OX=10116 GN=Nipsnap2 PE=1 SV=1 | Nipsnap2 | 34.9 | 32.941 | 10.524 |
| A0A0G2JUU7 | Gamma-tubulin complex component OS=Rattus norvegicus OX=10116 GN=Tubgcp3 PE=1 SV=1 | Tubgcp3 | 17.2 | 103.09 | 19.998 |
| Q2TA68 | "Dynamin-like 120 kDa protein, mitochondrial OS=Rattus norvegicus OX=10116 GN=Opa1 PE=1 SV=1" | Opa1 | 42.1 | 111.31 | 207.19 |
| D4A3E8 | Mitochondrial ribosomal protein S27 OS=Rattus norvegicus OX=10116 GN=Mrps27 PE=1 SV=1 | Mrps27 | 40.5 | 47.648 | 35.606 |
| Q68FX8 | Mitochondrial-processing peptidase subunit alpha OS=Rattus norvegicus OX=10116 GN=Pmpca PE=1 SV=1 | Pmpca | 36.5 | 58.154 | 301.91 |
| B0BN72 | Mapk-regulated corepressor-interacting protein 1 OS=Rattus norvegicus OX=10116 GN=Mcrip1 PE=1 SV=1 | Mcrip1 | 29.9 | 11.101 | 6.2638 |
| A0A0G2K8L9 | Mitochondrial ribosomal protein S28 OS=Rattus norvegicus OX=10116 GN=Mrps28 PE=1 SV=1 | Mrps28 | 32.1 | 20.578 | 30.34 |
| P48508 | Glutamate--cysteine ligase regulatory subunit OS=Rattus norvegicus OX=10116 GN=Gclm PE=1 SV=1 | Gclm | 21.9 | 30.548 | 7.0902 |
| D3ZIN7 | Mitochondrial ribosomal protein S23 OS=Rattus norvegicus OX=10116 GN=Mrps23 PE=1 SV=1 | Mrps23 | 35 | 20.321 | 15.469 |
| M0R7G4 | MICOS complex subunit OS=Rattus norvegicus OX=10116 GN=Apoo PE=1 SV=2 | Apoo | 35.2 | 22.369 | 8.2153 |
| B5DFI9 | Pdk3 protein OS=Rattus norvegicus OX=10116 GN=Pdk3 PE=2 SV=1 | Pdk3 | 54.2 | 47.943 | 61.283 |
| A0A0G2K199 | "Leprecan 1, isoform CRA_c OS=Rattus norvegicus OX=10116 GN=P3h1 PE=1 SV=1" | P3h1 | 26.8 | 83.513 | 24.512 |
| P11240 | "Cytochrome c oxidase subunit 5A, mitochondrial OS=Rattus norvegicus OX=10116 GN=Cox5a PE=1 SV=1" | Cox5a | 49.3 | 16.129 | 319.37 |
| D3ZHF8 | "Translation factor GUF1, mitochondrial OS=Rattus norvegicus OX=10116 GN=Guf1 PE=1 SV=1" | Guf1 | 16.2 | 73.206 | 20.396 |
| G3V7P3 | "39S ribosomal protein L3, mitochondrial OS=Rattus norvegicus OX=10116 GN=Mrpl3 PE=1 SV=1" | Mrpl3 | 19.3 | 38.492 | 21.428 |
| A0A0G2JSX1 | Non-structural maintenance of chromosomes element 1 homolog OS=Rattus norvegicus OX=10116 GN=Nsmce1 PE=4 SV=1 | Nsmce1 | 14.1 | 33.309 | 5.0332 |
| Q68FZ8 | "Propionyl coenzyme A carboxylase, beta polypeptide OS=Rattus norvegicus OX=10116 GN=Pccb PE=1 SV=1" | Pccb | 23.8 | 58.678 | 17.367 |
| G3V6W2 | Prolactin regulatory element-binding protein OS=Rattus norvegicus OX=10116 GN=Preb PE=1 SV=1 | Preb | 38.4 | 45.371 | 31.377 |
| Q3MHT2 | "Cysteine desulfurase, mitochondrial OS=Rattus norvegicus OX=10116 GN=Nfs1 PE=1 SV=1" | Nfs1 | 30.7 | 50.672 | 126.38 |
| P17764 | "Acetyl-CoA acetyltransferase, mitochondrial OS=Rattus norvegicus OX=10116 GN=Acat1 PE=1 SV=1" | Acat1 | 50.7 | 44.695 | 323.31 |
| A0A0G2K6D5 | Pitrilysin metallopeptidase 1 OS=Rattus norvegicus OX=10116 GN=Pitrm1 PE=1 SV=1 | Pitrm1 | 54.5 | 117.42 | 323.31 |
| A0A0G2KAU2 | Armadillo repeat-containing X-linked protein 3 OS=Rattus norvegicus OX=10116 GN=Armcx3 PE=1 SV=1 | Armcx3 | 17.4 | 47.344 | 6.0606 |
| Q6P686 | Osteoclast-stimulating factor 1 OS=Rattus norvegicus OX=10116 GN=Ostf1 PE=1 SV=1 | Ostf1 | 47.7 | 23.668 | 77.367 |
| P14604 | "Enoyl-CoA hydratase, mitochondrial OS=Rattus norvegicus OX=10116 GN=Echs1 PE=1 SV=1" | Echs1 | 26.2 | 31.516 | 53.96 |
| Q4KM65 | Cleavage and polyadenylation specificity factor subunit 5 OS=Rattus norvegicus OX=10116 GN=Nudt21 PE=2 SV=1 | Nudt21 | 59.9 | 26.24 | 158.29 |
| F1LMJ8 | "Calcium uptake protein 2, mitochondrial OS=Rattus norvegicus OX=10116 GN=Micu2 PE=1 SV=1" | Micu2 | 28.1 | 49.214 | 19.652 |
| Q9EPJ3 | "28S ribosomal protein S26, mitochondrial OS=Rattus norvegicus OX=10116 GN=Mrps26 PE=1 SV=1" | Mrps26 | 24 | 23.349 | 7.2188 |
| D4A104 | Mitochondrial ribosomal protein L45 OS=Rattus norvegicus OX=10116 GN=Mrpl45 PE=1 SV=1 | Mrpl45 | 32.4 | 35.439 | 23.062 |
| F1LM47 | "Succinate--CoA ligase [ADP-forming] subunit beta, mitochondrial OS=Rattus norvegicus OX=10116 GN=Sucla2 PE=1 SV=1" | Sucla2 | 32 | 50.306 | 192.49 |
| Q566E5 | KDEL motif-containing protein 2 OS=Rattus norvegicus OX=10116 GN=Kdelc2 PE=2 SV=1 | Kdelc2 | 6.9 | 58.701 | 2.9235 |
| Q5BJY6 | N-acetylglucosamine-6-phosphate deacetylase OS=Rattus norvegicus OX=10116 GN=Amdhd2 PE=3 SV=2 | Amdhd2 | 18.3 | 43.538 | 11.806 |
| Q7TQ84 | UAP56-interacting factor OS=Rattus norvegicus OX=10116 GN=Fyttd1 PE=2 SV=2 | Fyttd1 | 9.5 | 35.642 | 11.901 |
| Q5XI79 | "Protein arginine methyltransferase NDUFAF7, mitochondrial OS=Rattus norvegicus OX=10116 GN=Ndufaf7 PE=2 SV=1" | Ndufaf7 | 30.3 | 48.715 | 27.172 |
| A0A0G2K3V2 | Aflatoxin B1 aldehyde reductase member 2 OS=Rattus norvegicus OX=10116 GN=Akr7a2 PE=1 SV=1 | Akr7a2 | 21.7 | 37.526 | 17.347 |
| Q03344 | "ATPase inhibitor, mitochondrial OS=Rattus norvegicus OX=10116 GN=Atp5if1 PE=3 SV=2" | Atp5if1 | 32.7 | 12.248 | 6.7494 |
| Q9ES53 | Ubiquitin recognition factor in ER-associated degradation protein 1 OS=Rattus norvegicus OX=10116 GN=Ufd1 PE=1 SV=1 | Ufd1 | 37.1 | 34.485 | 24.193 |
| F1LM93 | Tyrosine-protein kinase Yes OS=Rattus norvegicus OX=10116 GN=Yes1 PE=1 SV=1 | Yes1 | 32 | 60.613 | 20.904 |
| P13832 | Myosin regulatory light chain RLC-A OS=Rattus norvegicus OX=10116 GN=Rlc-a PE=2 SV=2 | Rlc-a | 66.3 | 19.895 | 323.31 |
| D4A1Y5 | "Methylenetetrahydrofolate dehydrogenase (NADP+-dependent) 2, methenyltetrahydrofolate cyclohydrolase OS=Rattus norvegicus OX=10116 GN=Mthfd2 PE=3 SV=1" | Mthfd2 | 55.9 | 37.716 | 60.892 |
| I6L9G5 | Reticulocalbin-3 OS=Rattus norvegicus OX=10116 GN=Rcn3 PE=1 SV=1 | Rcn3 | 58.5 | 37.941 | 142.9 |
| Q4KLK9 | RNA polymerase II subunit A C-terminal domain phosphatase SSU72 OS=Rattus norvegicus OX=10116 GN=Ssu72 PE=2 SV=1 | Ssu72 | 20.1 | 22.544 | 3.0965 |
| Q6AY55 | Dephospho-CoA kinase domain-containing protein OS=Rattus norvegicus OX=10116 GN=Dcakd PE=2 SV=1 | Dcakd | 27.1 | 27.352 | 9.5579 |
| Q6PDW2 | 60S ribosomal protein L21 OS=Rattus norvegicus OX=10116 GN=Rpl21 PE=2 SV=1 | Rpl21 | 38.8 | 18.579 | 167.62 |
| D4A5L9 | Uncharacterized protein OS=Rattus norvegicus OX=10116 PE=3 SV=1 | --- | 54.3 | 11.635 | 323.31 |
| Q66X93 | Staphylococcal nuclease domain-containing protein 1 OS=Rattus norvegicus OX=10116 GN=Snd1 PE=1 SV=1 | Snd1 | 58.9 | 101.95 | 323.31 |
| Q6AYA2 | Vaccinia-related kinase 1 OS=Rattus norvegicus OX=10116 GN=Vrk1 PE=1 SV=1 | Vrk1 | 24.4 | 46.84 | 15.212 |
| G3V6D3 | ATP synthase subunit beta OS=Rattus norvegicus OX=10116 GN=Atp5f1b PE=1 SV=1 | Atp5f1b | 78.6 | 56.344 | 323.31 |
| Q64057 | Alpha-aminoadipic semialdehyde dehydrogenase OS=Rattus norvegicus OX=10116 GN=Aldh7a1 PE=1 SV=2 | Aldh7a1 | 35.8 | 58.748 | 111.91 |
| D4ADD7 | Glutaredoxin 5 OS=Rattus norvegicus OX=10116 GN=Glrx5 PE=1 SV=1 | Glrx5 | 20.4 | 16.432 | 19.461 |
| B2RZ27 | SH3 domain binding glutamic acid-rich protein-like 3 OS=Rattus norvegicus OX=10116 GN=Sh3bgrl3 PE=1 SV=1 | Sh3bgrl3 | 21.5 | 10.477 | 12.355 |
| Q68FS4 | Cytosol aminopeptidase OS=Rattus norvegicus OX=10116 GN=Lap3 PE=1 SV=1 | Lap3 | 45.5 | 56.149 | 178.09 |
| Q9R0I8 | Phosphatidylinositol 5-phosphate 4-kinase type-2 alpha OS=Rattus norvegicus OX=10116 GN=Pip4k2a PE=1 SV=1 | Pip4k2a | 17.5 | 46.209 | 17.408 |
| B0BN56 | "28S ribosomal protein S31, mitochondrial OS=Rattus norvegicus OX=10116 GN=Mrps31 PE=2 SV=1" | Mrps31 | 31.8 | 43.961 | 26.381 |
| F1LXS5 | REST corepressor 1 OS=Rattus norvegicus OX=10116 GN=Rcor1 PE=1 SV=1 | Rcor1 | 24.3 | 42.073 | 16.047 |
| D4A833 | Mitochondrial ribosomal protein S30 OS=Rattus norvegicus OX=10116 GN=Mrps30 PE=1 SV=1 | Mrps30 | 18.6 | 50.001 | 49.375 |
| D3ZUX7 | Acyl-CoA synthetase family member 3 OS=Rattus norvegicus OX=10116 GN=Acsf3 PE=1 SV=1 | Acsf3 | 21.4 | 65.265 | 10.776 |
| O35796 | "Complement component 1 Q subcomponent-binding protein, mitochondrial OS=Rattus norvegicus OX=10116 GN=C1qbp PE=1 SV=2" | C1qbp | 41.9 | 30.997 | 323.31 |
| M0RAK4 | "Frataxin, mitochondrial OS=Rattus norvegicus OX=10116 GN=Fxn PE=1 SV=2" | Fxn | 20.9 | 22.883 | 11.903 |
| P04182 | "Ornithine aminotransferase, mitochondrial OS=Rattus norvegicus OX=10116 GN=Oat PE=1 SV=1" | Oat | 71.8 | 48.332 | 263.77 |
| A0A0G2K9B4 | Mitochondrial ribosomal protein L15 OS=Rattus norvegicus OX=10116 GN=Mrpl15 PE=1 SV=1 | Mrpl15 | 32.9 | 33.652 | 8.6882 |
| P21571 | "ATP synthase-coupling factor 6, mitochondrial OS=Rattus norvegicus OX=10116 GN=Atp5pf PE=1 SV=1" | Atp5pf | 22.2 | 12.494 | 209.22 |
| F1LML7 | Huntingtin-interacting protein 1-related OS=Rattus norvegicus OX=10116 GN=Hip1r PE=1 SV=1 | Hip1r | 39.6 | 119.55 | 132.85 |
| F1M8F0 | RNA-binding motif protein 7 OS=Rattus norvegicus OX=10116 GN=Rbm7 PE=1 SV=3 | Rbm7 | 22.7 | 22.789 | 12.56 |
| Q5PQV5 | Trophoblast glycoprotein OS=Rattus norvegicus OX=10116 GN=Tpbg PE=1 SV=1 | Tpbg | 17.8 | 46.508 | 17.589 |
| Q06647 | "ATP synthase subunit O, mitochondrial OS=Rattus norvegicus OX=10116 GN=Atp5po PE=1 SV=1" | Atp5po | 48.4 | 23.397 | 323.31 |
| G3V728 | "4-nitrophenylphosphatase domain and non-neuronal SNAP25-like protein homolog 1 (C. elegans), isoform CRA_b OS=Rattus norvegicus OX=10116 GN=Nipsnap1 PE=1 SV=1" | Nipsnap1 | 43 | 33.346 | 18.037 |
| D3ZFQ8 | Cytochrome c-1 OS=Rattus norvegicus OX=10116 GN=Cyc1 PE=1 SV=3 | Cyc1 | 35.3 | 35.434 | 206.94 |
| G3V8U8 | Branched-chain-amino-acid aminotransferase OS=Rattus norvegicus OX=10116 GN=Bcat2 PE=1 SV=1 | Bcat2 | 23.9 | 44.229 | 28 |
| B1WC67 | RCG29001 OS=Rattus norvegicus OX=10116 GN=Slc25a24 PE=1 SV=1 | Slc25a24 | 54.5 | 52.89 | 323.31 |
| D3ZG43 | "NADH dehydrogenase (Ubiquinone) Fe-S protein 3 (Predicted), isoform CRA_c OS=Rattus norvegicus OX=10116 GN=Ndufs3 PE=1 SV=1" | Ndufs3 | 36 | 30.226 | 19.536 |
| P97519 | "Hydroxymethylglutaryl-CoA lyase, mitochondrial OS=Rattus norvegicus OX=10116 GN=Hmgcl PE=2 SV=1" | Hmgcl | 25.8 | 34.191 | 11.757 |
| D4A9M4 | Periphilin 1 OS=Rattus norvegicus OX=10116 GN=Pphln1 PE=1 SV=1 | Pphln1 | 10 | 47.317 | 4.2367 |
| P06214 | Delta-aminolevulinic acid dehydratase OS=Rattus norvegicus OX=10116 GN=Alad PE=1 SV=1 | Alad | 22.4 | 36.031 | 19.643 |
| D4ACE9 | "Alpha-aminoadipic semialdehyde synthase, mitochondrial OS=Rattus norvegicus OX=10116 GN=Aass PE=1 SV=3" | Aass | 15.7 | 103.11 | 25.364 |
| P56574 | "Isocitrate dehydrogenase [NADP], mitochondrial OS=Rattus norvegicus OX=10116 GN=Idh2 PE=1 SV=2" | Idh2 | 45.4 | 50.967 | 323.31 |
| F1LTU4 | Ribosome assembly factor mrt4 OS=Rattus norvegicus OX=10116 GN=Mrto4 PE=1 SV=2 | Mrto4 | 37.3 | 22.162 | 37.637 |
| B0BMW2 | 3-hydroxyacyl-CoA dehydrogenase type-2 OS=Rattus norvegicus OX=10116 GN=Hsd17b10 PE=1 SV=1 | Hsd17b10 | 86.2 | 27.249 | 323.31 |
| F1LUD3 | Uncharacterized protein OS=Rattus norvegicus OX=10116 PE=1 SV=2 | --- | 41.2 | 584.8 | 317.45 |
| D4A9Z6 | Mitochondrial ribosomal protein S35 OS=Rattus norvegicus OX=10116 GN=Mrps35 PE=1 SV=1 | Mrps35 | 35.3 | 36.206 | 15.48 |
| A0A0G2JU12 | Microsomal glutathione S-transferase 2 OS=Rattus norvegicus OX=10116 GN=Mgst2 PE=1 SV=1 | Mgst2 | 19.5 | 14.828 | 2.7583 |
| P36506 | Dual specificity mitogen-activated protein kinase kinase 2 OS=Rattus norvegicus OX=10116 GN=Map2k2 PE=1 SV=1 | Map2k2 | 28.5 | 44.281 | 9.5388 |
| F1LPZ1 | Rho GTPase-activating protein 29 OS=Rattus norvegicus OX=10116 GN=Arhgap29 PE=1 SV=2 | Arhgap29 | 5 | 142.47 | 3.8287 |
| F1LMZ4 | "Ribosome-releasing factor 2, mitochondrial OS=Rattus norvegicus OX=10116 GN=Gfm2 PE=1 SV=2" | Gfm2 | 18 | 85.913 | 19.586 |
| M0R567 | Interferon regulatory factor 2-binding protein-like OS=Rattus norvegicus OX=10116 GN=Irf2bpl PE=1 SV=1 | Irf2bpl | 19.9 | 81.367 | 10.166 |
| A0A0H2UHE1 | "Succinate--CoA ligase [ADP/GDP-forming] subunit alpha, mitochondrial OS=Rattus norvegicus OX=10116 GN=Suclg1 PE=1 SV=1" | Suclg1 | 38.6 | 37.559 | 323.31 |
| A0A0G2JW49 | E3 SUMO-protein ligase NSE2 OS=Rattus norvegicus OX=10116 GN=Nsmce2 PE=4 SV=1 | Nsmce2 | 13 | 24.561 | 2.6583 |
| Q5I0P2 | "Glycine cleavage system H protein, mitochondrial OS=Rattus norvegicus OX=10116 GN=Gcsh PE=2 SV=1" | Gcsh | 52.4 | 18.485 | 176.54 |
| P10888 | "Cytochrome c oxidase subunit 4 isoform 1, mitochondrial OS=Rattus norvegicus OX=10116 GN=Cox4i1 PE=1 SV=1" | Cox4i1 | 39.1 | 19.514 | 70.028 |
| M0RCP2 | SH3 and PX domains 2B OS=Rattus norvegicus OX=10116 GN=Sh3pxd2b PE=4 SV=1 | Sh3pxd2b | 37.2 | 26.642 | 40.947 |
| Q6AXY4 | DNA polymerase delta subunit 2 OS=Rattus norvegicus OX=10116 GN=Pold2 PE=1 SV=1 | Pold2 | 20.9 | 51.346 | 8.6988 |
| Q4FZT0 | "Stomatin-like protein 2, mitochondrial OS=Rattus norvegicus OX=10116 GN=Stoml2 PE=1 SV=1" | Stoml2 | 58.4 | 38.413 | 261.48 |
| D3ZTW8 | Mitochondrial ribosomal protein L27 OS=Rattus norvegicus OX=10116 GN=Mrpl27 PE=1 SV=2 | Mrpl27 | 29.1 | 15.823 | 6.9174 |
| D3ZEG8 | Translocase of inner mitochondrial membrane 29 OS=Rattus norvegicus OX=10116 GN=Timm29 PE=1 SV=1 | Timm29 | 32.2 | 29.873 | 48.497 |
| D4A4W6 | "RCG20695, isoform CRA_b OS=Rattus norvegicus OX=10116 GN=Slirp PE=1 SV=1" | Slirp | 86.5 | 12.576 | 68.555 |
| P97570 | 85/88 kDa calcium-independent phospholipase A2 OS=Rattus norvegicus OX=10116 GN=Pla2g6 PE=1 SV=2 | Pla2g6 | 7.7 | 89.555 | 7.4838 |
| Q5U2U0 | "ATP-dependent Clp protease ATP-binding subunit clpX-like, mitochondrial OS=Rattus norvegicus OX=10116 GN=Clpx PE=2 SV=1" | Clpx | 37.6 | 69.207 | 41.785 |
| Q5I0I4 | Distal membrane-arm assembly complex protein 2 OS=Rattus norvegicus OX=10116 GN=Dmac2 PE=2 SV=1 | Dmac2 | 18.5 | 28.507 | 4.517 |
| B0BNB9 | HtrA serine peptidase 2 OS=Rattus norvegicus OX=10116 GN=Htra2 PE=1 SV=1 | Htra2 | 28.8 | 49.094 | 51.145 |
| A0A0H2UHT3 | "39S ribosomal protein L22, mitochondrial OS=Rattus norvegicus OX=10116 GN=Mrpl22 PE=1 SV=1" | Mrpl22 | 17.9 | 25.865 | 3.1805 |
| B2RYT4 | Mitochondrial ribosomal protein S14 OS=Rattus norvegicus OX=10116 GN=Mrps14 PE=1 SV=1 | Mrps14 | 21.9 | 14.924 | 5.6288 |
| B5DF07 | Mitochondrial ribonuclease P catalytic subunit OS=Rattus norvegicus OX=10116 GN=Mrpp3 PE=2 SV=1 | Mrpp3 | 10.4 | 67.007 | 6.2916 |
| D3ZXK4 | Abhydrolase domain-containing 11 OS=Rattus norvegicus OX=10116 GN=Abhd11 PE=1 SV=1 | Abhd11 | 39.7 | 33.215 | 7.0494 |
| F7F189 | NDC80 kinetochore complex component OS=Rattus norvegicus OX=10116 GN=Ndc80 PE=2 SV=2 | Ndc80 | 9.2 | 73.761 | 5.0412 |
| Q497C2 | RCG55882 OS=Rattus norvegicus OX=10116 GN=Rpp38 PE=1 SV=1 | Rpp38 | 19.1 | 30.087 | 4.7229 |
| M0R776 | Mitochondrial ribosomal protein S36 OS=Rattus norvegicus OX=10116 GN=Mrps36 PE=1 SV=1 | Mrps36 | 62.1 | 11.413 | 37.452 |
| D4A4L5 | Iron-sulfur cluster assembly 2 OS=Rattus norvegicus OX=10116 GN=Isca2 PE=1 SV=1 | Isca2 | 37.7 | 16.68 | 11.111 |
| A0A0G2JW34 | CDGSH iron sulfur domain 3 OS=Rattus norvegicus OX=10116 GN=Cisd3 PE=1 SV=1 | Cisd3 | 11.8 | 14.379 | 3.9163 |
| P54645 | 5'-AMP-activated protein kinase catalytic subunit alpha-1 OS=Rattus norvegicus OX=10116 GN=Prkaa1 PE=1 SV=2 | Prkaa1 | 35.1 | 63.973 | 38.636 |
| D4ACM1 | Elongator complex protein 3 OS=Rattus norvegicus OX=10116 GN=Elp3 PE=1 SV=2 | Elp3 | 19.9 | 62.36 | 20.34 |
| Q4FZT8 | SPRY domain-containing protein 4 OS=Rattus norvegicus OX=10116 GN=Spryd4 PE=2 SV=1 | Spryd4 | 15.5 | 23.249 | 3.7643 |
| Q6P7Q1 | BRISC and BRCA1-A complex member 2 OS=Rattus norvegicus OX=10116 GN=Babam2 PE=1 SV=1 | Babam2 | 7.6 | 43.558 | 2.0911 |
| B0BN48 | Cyclin-dependent kinase 2-associated protein OS=Rattus norvegicus OX=10116 GN=Cdk2ap1 PE=2 SV=1 | Cdk2ap1 | 20.2 | 12.354 | 3.6566 |
| Q8R4Z9 | Mitofusin-1 OS=Rattus norvegicus OX=10116 GN=Mfn1 PE=1 SV=1 | Mfn1 | 12.4 | 83.846 | 5.7748 |
| F1LN88 | "Aldehyde dehydrogenase, mitochondrial OS=Rattus norvegicus OX=10116 GN=Aldh2 PE=1 SV=2" | Aldh2 | 46.1 | 56.516 | 323.31 |
| G3V945 | "Aldehyde dehydrogenase family 5, subfamily A1 OS=Rattus norvegicus OX=10116 GN=Aldh5a1 PE=1 SV=1" | Aldh5a1 | 14.1 | 56.145 | 5.6422 |
| D3ZAN3 | Alpha glucosidase 2 alpha neutral subunit (Predicted) OS=Rattus norvegicus OX=10116 GN=Ganab PE=1 SV=1 | Ganab | 42.9 | 90.571 | 224.92 |
| A0A0G2K1S4 | Zinc finger protein 330 OS=Rattus norvegicus OX=10116 GN=Zfp330 PE=1 SV=1 | Zfp330 | 39.2 | 30.659 | 27.592 |
| Q5XIF3 | "NADH dehydrogenase [ubiquinone] iron-sulfur protein 4, mitochondrial OS=Rattus norvegicus OX=10116 GN=Ndufs4 PE=1 SV=1" | Ndufs4 | 41.7 | 19.74 | 12.867 |
| F1LR87 | Beta-hexosaminidase OS=Rattus norvegicus OX=10116 GN=Hexb PE=1 SV=3 | Hexb | 34.2 | 61.5 | 319.77 |
| B2GUZ3 | Methylenetetrahydrofolate dehydrogenase (NADP+-dependent) 1-like OS=Rattus norvegicus OX=10116 GN=Mthfd1l PE=1 SV=1 | Mthfd1l | 64 | 105.84 | 323.31 |
| P63039 | "60 kDa heat shock protein, mitochondrial OS=Rattus norvegicus OX=10116 GN=Hspd1 PE=1 SV=1" | Hspd1 | 76.6 | 60.955 | 323.31 |
| P02401 | 60S acidic ribosomal protein P2 OS=Rattus norvegicus OX=10116 GN=Rplp2 PE=1 SV=2 | Rplp2 | 88.7 | 11.692 | 323.31 |
| P70583 | Deoxyuridine 5'-triphosphate nucleotidohydrolase OS=Rattus norvegicus OX=10116 GN=Dut PE=2 SV=3 | Dut | 43.9 | 22.003 | 24.724 |
| B2GUW4 | Exdl2 protein OS=Rattus norvegicus OX=10116 GN=Exd2 PE=2 SV=1 | Exd2 | 9 | 74.059 | 19.284 |
| Q03346 | Mitochondrial-processing peptidase subunit beta OS=Rattus norvegicus OX=10116 GN=Pmpcb PE=1 SV=3 | Pmpcb | 35.2 | 54.265 | 141.39 |
| A0A0G2JZH8 | Dihydrolipoamide acetyltransferase component of pyruvate dehydrogenase complex OS=Rattus norvegicus OX=10116 GN=Pdhx PE=1 SV=1 | Pdhx | 28.1 | 54.169 | 106.86 |
| P84083 | ADP-ribosylation factor 5 OS=Rattus norvegicus OX=10116 GN=Arf5 PE=1 SV=2 | Arf5 | 50 | 20.529 | 11.875 |
| A0A0G2KB56 | "Glutamine fructose-6-phosphate transaminase 1, isoform CRA_a OS=Rattus norvegicus OX=10116 GN=Gfpt1 PE=1 SV=1" | Gfpt1 | 40.3 | 78.918 | 53.706 |
| Q5BK63 | "NADH dehydrogenase [ubiquinone] 1 alpha subcomplex subunit 9, mitochondrial OS=Rattus norvegicus OX=10116 GN=Ndufa9 PE=1 SV=2" | Ndufa9 | 46.9 | 42.559 | 67.506 |
| Q6PDU2 | Mitochondrial ribosomal protein S18A OS=Rattus norvegicus OX=10116 GN=Mrps18a PE=1 SV=1 | Mrps18a | 19.4 | 22.235 | 5.3696 |
| A0A0G2K4A0 | RAB11 family-interacting protein 5 OS=Rattus norvegicus OX=10116 GN=Rab11fip5 PE=1 SV=1 | Rab11fip5 | 39 | 68.761 | 7.8379 |
| Q4FZU0 | "Acid phosphatase 6, lysophosphatidic OS=Rattus norvegicus OX=10116 GN=Acp6 PE=1 SV=1" | Acp6 | 13.3 | 47.28 | 2.8835 |
| F7FDM3 | Mitochondrial translational initiation factor 2 OS=Rattus norvegicus OX=10116 GN=Mtif2 PE=1 SV=2 | Mtif2 | 13.3 | 79.21 | 14.468 |
| Q4V7D6 | NIF3-like protein 1 OS=Rattus norvegicus OX=10116 GN=Nif3l1 PE=1 SV=1 | Nif3l1 | 12 | 41.518 | 2.3248 |
| Q6AY53 | Deoxyhypusine synthase OS=Rattus norvegicus OX=10116 GN=Dhps PE=2 SV=1 | Dhps | 22.2 | 40.705 | 5.1381 |
| A0A0G2K8F6 | Alpha-mannosidase OS=Rattus norvegicus OX=10116 GN=Man2b2 PE=1 SV=1 | Man2b2 | 12.6 | 114.87 | 13.384 |
| D4A4A9 | Mitochondrial ribosomal protein L19 OS=Rattus norvegicus OX=10116 GN=Mrpl19 PE=1 SV=1 | Mrpl19 | 34.2 | 33.633 | 11.888 |
| Q6PDU7 | "ATP synthase subunit g, mitochondrial OS=Rattus norvegicus OX=10116 GN=Atp5mg PE=1 SV=2" | Atp5mg | 58.3 | 11.433 | 323.31 |
| O55173 | 3-phosphoinositide-dependent protein kinase 1 OS=Rattus norvegicus OX=10116 GN=Pdpk1 PE=1 SV=2 | Pdpk1 | 12.3 | 63.593 | 4.0273 |
| Q5XFW4 | Mitochondrial ribosomal protein L13 OS=Rattus norvegicus OX=10116 GN=Mrpl13 PE=1 SV=1 | Mrpl13 | 28.1 | 20.54 | 4.6129 |
| D3ZVN7 | Protoporphyrinogen oxidase OS=Rattus norvegicus OX=10116 GN=Ppox PE=1 SV=1 | Ppox | 16.6 | 50.784 | 13.166 |
| A0A0G2K3W1 | von Willebrand factor A domain-containing 8 OS=Rattus norvegicus OX=10116 GN=Vwa8 PE=1 SV=1 | Vwa8 | 16 | 53.76 | 4.9683 |
| D4A601 | Translational activator of cytochrome c oxidase 1 OS=Rattus norvegicus OX=10116 GN=Taco1 PE=1 SV=3 | Taco1 | 26.1 | 32.812 | 20.263 |
| A0A0G2JYK4 | "Transcription elongation factor, mitochondrial OS=Rattus norvegicus OX=10116 GN=Tefm PE=1 SV=1" | Tefm | 15.7 | 38.434 | 2.4178 |
| F1MA54 | "[Pyruvate dehydrogenase (acetyl-transferring)] kinase isozyme 1, mitochondrial OS=Rattus norvegicus OX=10116 GN=Pdk1 PE=1 SV=2" | Pdk1 | 35.3 | 49.098 | 36.111 |
| D4A4H5 | "Stromal cell derived factor 2 (Predicted), isoform CRA_b OS=Rattus norvegicus OX=10116 GN=Sdf2 PE=1 SV=2" | Sdf2 | 34.7 | 24.056 | 18.115 |
| D4ADL7 | Xyloside xylosyltransferase 1 OS=Rattus norvegicus OX=10116 GN=Xxylt1 PE=4 SV=1 | Xxylt1 | 31.4 | 43.912 | 11.791 |
| Q62639 | GTP-binding protein Rheb OS=Rattus norvegicus OX=10116 GN=Rheb PE=1 SV=1 | Rheb | 17.4 | 20.479 | 1.6686 |
| P19944 | 60S acidic ribosomal protein P1 OS=Rattus norvegicus OX=10116 GN=Rplp1 PE=3 SV=1 | Rplp1 | 94.7 | 11.498 | 323.31 |
| Q4G067 | Mitochondrial ribosomal protein L44 OS=Rattus norvegicus OX=10116 GN=Mrpl44 PE=1 SV=1 | Mrpl44 | 21.1 | 37.44 | 45.905 |
| Q5XIE6 | "3-hydroxyisobutyryl-CoA hydrolase, mitochondrial OS=Rattus norvegicus OX=10116 GN=Hibch PE=1 SV=2" | Hibch | 20.5 | 43.024 | 11.344 |
| Q3KRD0 | "Aspartate--tRNA ligase, mitochondrial OS=Rattus norvegicus OX=10116 GN=Dars2 PE=1 SV=1" | Dars2 | 12.3 | 73.952 | 3.0476 |
| B0BNM1 | NAD(P)H-hydrate epimerase OS=Rattus norvegicus OX=10116 GN=Naxe PE=2 SV=1 | Naxe | 22 | 30.89 | 6.7369 |
| Q5U2X8 | Acyl-CoA thioesterase 9 OS=Rattus norvegicus OX=10116 GN=Acot9 PE=1 SV=1 | Acot9 | 40.5 | 50.443 | 82.787 |
| O89049 | "Thioredoxin reductase 1, cytoplasmic OS=Rattus norvegicus OX=10116 GN=Txnrd1 PE=1 SV=5" | Txnrd1 | 37.3 | 54.67 | 26.943 |
| D4A7X1 | Mitochondrial ribosomal protein S16 OS=Rattus norvegicus OX=10116 GN=Mrps16 PE=1 SV=1 | Mrps16 | 39.3 | 15.243 | 10.683 |
| A0A096MKG5 | "NAD kinase 2, mitochondrial OS=Rattus norvegicus OX=10116 GN=Nadk2 PE=1 SV=1" | Nadk2 | 10 | 50.654 | 4.8243 |
| Q07803 | "Elongation factor G, mitochondrial OS=Rattus norvegicus OX=10116 GN=Gfm1 PE=1 SV=2" | Gfm1 | 27 | 83.456 | 48.341 |
| P59215 | Guanine nucleotide-binding protein G(o) subunit alpha OS=Rattus norvegicus OX=10116 GN=Gnao1 PE=1 SV=2 | Gnao1 | 35.3 | 40.068 | 106.9 |
| Q5XHZ0 | "Heat shock protein 75 kDa, mitochondrial OS=Rattus norvegicus OX=10116 GN=Trap1 PE=1 SV=1" | Trap1 | 49.3 | 80.46 | 323.31 |
| D4AD36 | FCH and double SH3 domains 1 OS=Rattus norvegicus OX=10116 GN=Fchsd1 PE=1 SV=2 | Fchsd1 | 7.5 | 76.766 | 4.2869 |
| P62815 | "V-type proton ATPase subunit B, brain isoform OS=Rattus norvegicus OX=10116 GN=Atp6v1b2 PE=1 SV=1" | Atp6v1b2 | 46.6 | 56.55 | 95.263 |
| P32551 | "Cytochrome b-c1 complex subunit 2, mitochondrial OS=Rattus norvegicus OX=10116 GN=Uqcrc2 PE=1 SV=2" | Uqcrc2 | 44.9 | 48.396 | 175.5 |
| D4ADS9 | EFR3 homolog A OS=Rattus norvegicus OX=10116 GN=Efr3a PE=1 SV=1 | Efr3a | 41.8 | 92.523 | 106.66 |
| Q4V7E5 | "Peptide chain release factor 1-like, mitochondrial OS=Rattus norvegicus OX=10116 GN=Mtrf1l PE=2 SV=1" | Mtrf1l | 21.2 | 42.343 | 6.4896 |
| P57113 | Maleylacetoacetate isomerase OS=Rattus norvegicus OX=10116 GN=Gstz1 PE=1 SV=2 | Gstz1 | 38 | 23.961 | 10.068 |
| D3ZI16 | "COP9 (Constitutive photomorphogenic) homolog, subunit 6 (Arabidopsis thaliana) (Predicted), isoform CRA_a OS=Rattus norvegicus OX=10116 GN=Cops6 PE=1 SV=1" | Cops6 | 39.2 | 37.702 | 63.628 |
| A0A0G2JYN4 | ERGIC and golgi 2 OS=Rattus norvegicus OX=10116 GN=Ergic2 PE=1 SV=1 | Ergic2 | 16.7 | 42.478 | 5.7568 |
| Q7M0E3 | Destrin OS=Rattus norvegicus OX=10116 GN=Dstn PE=1 SV=3 | Dstn | 61.2 | 18.533 | 108.77 |
| F1M173 | Contactin-5 OS=Rattus norvegicus OX=10116 GN=Cntn5 PE=4 SV=3 | Cntn5 | 1.1 | 120.46 | 1.7937 |
| B0BMT9 | Sqrdl protein OS=Rattus norvegicus OX=10116 GN=Sqor PE=1 SV=1 | Sqor | 56 | 50.201 | 187.47 |
| D3Z8C7 | WD repeat domain 48 OS=Rattus norvegicus OX=10116 GN=Wdr48 PE=1 SV=1 | Wdr48 | 20.7 | 76.079 | 11.891 |
| F7F3Z1 | "Lectin, mannose-binding 2-like OS=Rattus norvegicus OX=10116 GN=Lman2l PE=1 SV=2" | Lman2l | 19.2 | 40.087 | 6.594 |
| Q9QYU2 | "Elongation factor Ts, mitochondrial OS=Rattus norvegicus OX=10116 GN=Tsfm PE=2 SV=1" | Tsfm | 53.4 | 35.178 | 106.29 |
| D4A465 | "Late endosomal/lysosomal adaptor, MAPK and MTOR activator 2 OS=Rattus norvegicus OX=10116 GN=Lamtor2 PE=1 SV=1" | Lamtor2 | 56.8 | 13.48 | 7.0516 |
| Q641X9 | "39S ribosomal protein L9, mitochondrial OS=Rattus norvegicus OX=10116 GN=Mrpl9 PE=2 SV=1" | Mrpl9 | 23.7 | 30.076 | 10.967 |
| Q68FS1 | Cytosolic Fe-S cluster assembly factor NUBP2 OS=Rattus norvegicus OX=10116 GN=Nubp2 PE=1 SV=1 | Nubp2 | 41.7 | 28.926 | 19.84 |
| B2RZ79 | Iron-sulfur cluster assembly enzyme OS=Rattus norvegicus OX=10116 GN=Iscu PE=1 SV=1 | Iscu | 34.1 | 17.967 | 33.813 |
| Q0D2L2 | Mitochondrial ribosomal protein S22 OS=Rattus norvegicus OX=10116 GN=Mrps22 PE=1 SV=1 | Mrps22 | 32.6 | 41.235 | 69.483 |
| Q5I0L3 | "Tyrosine--tRNA ligase, mitochondrial OS=Rattus norvegicus OX=10116 GN=Yars2 PE=2 SV=1" | Yars2 | 42.3 | 52.627 | 31.055 |
| D3ZS58 | NADH dehydrogenase [ubiquinone] 1 alpha subcomplex subunit 2 OS=Rattus norvegicus OX=10116 GN=Ndufa2 PE=1 SV=1 | Ndufa2 | 25.8 | 10.844 | 2.903 |
| Q7TP77 | Aa2-277 OS=Rattus norvegicus OX=10116 GN=Mrpl49 PE=1 SV=1 | Mrpl49 | 22.3 | 19.406 | 12.324 |
| Q9EQX9 | Ubiquitin-conjugating enzyme E2 N OS=Rattus norvegicus OX=10116 GN=Ube2n PE=1 SV=1 | Ube2n | 55.3 | 17.124 | 116.33 |
| D3ZTR1 | Mitochondrial ribosomal protein S17 OS=Rattus norvegicus OX=10116 GN=Mrps17 PE=1 SV=1 | Mrps17 | 51.2 | 13.999 | 29.283 |
| D3ZA85 | "Histone cell cycle regulation defective interacting protein 5 (Predicted), isoform CRA_a OS=Rattus norvegicus OX=10116 GN=Nfu1 PE=1 SV=1" | Nfu1 | 17.4 | 28.367 | 35.295 |
| O88377 | Phosphatidylinositol 5-phosphate 4-kinase type-2 beta OS=Rattus norvegicus OX=10116 GN=Pip4k2b PE=1 SV=1 | Pip4k2b | 18.3 | 47.263 | 10.962 |
| A1L1J8 | "RAB5B, member RAS oncogene family OS=Rattus norvegicus OX=10116 GN=Rab5b PE=1 SV=1" | Rab5b | 50.2 | 23.674 | 34.485 |
| G3V878 | Signal peptidase complex catalytic subunit SEC11 OS=Rattus norvegicus OX=10116 GN=Sec11c PE=1 SV=1 | Sec11c | 18.8 | 21.66 | 5.8697 |
| Q5FVF3 | "Golgi-associated, gamma adaptin ear-containing, ARF-binding protein 1 OS=Rattus norvegicus OX=10116 GN=Gga1 PE=1 SV=1" | Gga1 | 15.1 | 61.762 | 5.8265 |
| D3ZR49 | "alpha-1,2-Mannosidase OS=Rattus norvegicus OX=10116 GN=Man1a2 PE=1 SV=3" | Man1a2 | 14.2 | 72.712 | 9 |
| A0A0G2JSW0 | Myosin regulatory light chain 12B OS=Rattus norvegicus OX=10116 GN=Myl12b PE=4 SV=1 | Myl12b | 66.3 | 19.779 | 107.51 |
| D3ZAI6 | 5'-nucleotidase domain-containing 3 OS=Rattus norvegicus OX=10116 GN=Nt5dc3 PE=1 SV=2 | Nt5dc3 | 27.7 | 63.126 | 12.871 |
| P45592 | Cofilin-1 OS=Rattus norvegicus OX=10116 GN=Cfl1 PE=1 SV=3 | Cfl1 | 62.7 | 18.532 | 323.31 |
| Q5U1Z8 | "Protein preY, mitochondrial OS=Rattus norvegicus OX=10116 GN=Pyurf PE=3 SV=1" | Pyurf | 14.3 | 12.652 | 2.0963 |
| Q7TP03 | Da2-24 OS=Rattus norvegicus OX=10116 GN=Glt8d1 PE=2 SV=1 | Glt8d1 | 23.3 | 45.226 | 8.0649 |
| D3ZQB6 | "Cat eye syndrome chromosome region, candidate 5 homolog (Human) (Predicted) OS=Rattus norvegicus OX=10116 GN=Hdhd5 PE=1 SV=1" | Hdhd5 | 13.6 | 46.476 | 5.5228 |
| G3V6U3 | "ALG2, alpha-1,3/1,6-mannosyltransferase OS=Rattus norvegicus OX=10116 GN=Alg2 PE=1 SV=1" | Alg2 | 47.2 | 47.318 | 28.518 |
| Q6P6R2 | "Dihydrolipoyl dehydrogenase, mitochondrial OS=Rattus norvegicus OX=10116 GN=Dld PE=1 SV=1" | Dld | 48.1 | 54.037 | 323.31 |
| Q5RK17 | Diablo homolog (Drosophila) OS=Rattus norvegicus OX=10116 GN=Diablo PE=1 SV=1 | Diablo | 39.7 | 26.906 | 41.187 |
| D3ZMR9 | Mitochondrial ribosomal protein L21 OS=Rattus norvegicus OX=10116 GN=Mrpl21 PE=1 SV=1 | Mrpl21 | 21.1 | 23.39 | 3.5382 |
| D3ZRC4 | Patatin-like phospholipase domain-containing 8 OS=Rattus norvegicus OX=10116 GN=Pnpla8 PE=1 SV=1 | Pnpla8 | 8.5 | 87.96 | 5.9604 |
| P62744 | AP-2 complex subunit sigma OS=Rattus norvegicus OX=10116 GN=Ap2s1 PE=1 SV=1 | Ap2s1 | 33.1 | 17.018 | 17.506 |
| Q5XIW9 | Flot2 protein OS=Rattus norvegicus OX=10116 GN=Flot2 PE=1 SV=1 | Flot2 | 52.1 | 38.717 | 84.551 |
| D4A2F2 | Par-6 (Partitioning defective 6) homolog beta (C. elegans) (Predicted) OS=Rattus norvegicus OX=10116 GN=Pard6b PE=1 SV=1 | Pard6b | 25.8 | 40.95 | 24.856 |
| A0A0G2K466 | "Oxidoreductase NAD-binding domain containing 1 (Predicted), isoform CRA_a OS=Rattus norvegicus OX=10116 GN=Oxnad1 PE=1 SV=1" | Oxnad1 | 28.1 | 34.847 | 36.75 |
| Q5M9G9 | FAST kinase domain-containing protein 4 OS=Rattus norvegicus OX=10116 GN=Tbrg4 PE=2 SV=1 | Tbrg4 | 27 | 71.18 | 26.732 |
| Q68FT3 | Pyridine nucleotide-disulfide oxidoreductase domain-containing protein 2 OS=Rattus norvegicus OX=10116 GN=Pyroxd2 PE=2 SV=1 | Pyroxd2 | 15.7 | 62.878 | 8.3771 |
| Q6P7R8 | Very-long-chain 3-oxoacyl-CoA reductase OS=Rattus norvegicus OX=10116 GN=Hsd17b12 PE=2 SV=1 | Hsd17b12 | 48.1 | 34.84 | 176.59 |
| A0A0G2K4W2 | Transcription factor BTF3 OS=Rattus norvegicus OX=10116 PE=3 SV=1 | --- | 63.9 | 17.18 | 139.38 |
| A0A0G2QC17 | "Protein phosphatase 2C, magnesium dependent, catalytic subunit, isoform CRA_a OS=Rattus norvegicus OX=10116 GN=Pdp1 PE=1 SV=1" | Pdp1 | 25 | 63.835 | 52.706 |
| Q91ZW6 | "Trimethyllysine dioxygenase, mitochondrial OS=Rattus norvegicus OX=10116 GN=Tmlhe PE=1 SV=2" | Tmlhe | 21.1 | 49.567 | 5.9117 |
| O08776 | NADH dehydrogenase [ubiquinone] 1 alpha subcomplex assembly factor 3 OS=Rattus norvegicus OX=10116 GN=Ndufaf3 PE=2 SV=1 | Ndufaf3 | 31.9 | 20.696 | 18.776 |
| B0BNE6 | "NADH dehydrogenase (Ubiquinone) Fe-S protein 8 (Predicted), isoform CRA_a OS=Rattus norvegicus OX=10116 GN=Ndufs8 PE=1 SV=1" | Ndufs8 | 23.1 | 23.97 | 18.84 |
| Q5XIC0 | "Enoyl-CoA delta isomerase 2, mitochondrial OS=Rattus norvegicus OX=10116 GN=Eci2 PE=1 SV=1" | Eci2 | 38.6 | 43.021 | 31.149 |
| Q63750 | "39S ribosomal protein L23, mitochondrial OS=Rattus norvegicus OX=10116 GN=Mrpl23 PE=2 SV=1" | Mrpl23 | 61 | 17.05 | 40.94 |
| Q6IRH7 | Caseinolytic peptidase B protein homolog OS=Rattus norvegicus OX=10116 GN=Clpb PE=1 SV=1 | Clpb | 29.7 | 75.678 | 30.098 |
| Q6PCT8 | "Succinate dehydrogenase [ubiquinone] cytochrome b small subunit, mitochondrial OS=Rattus norvegicus OX=10116 GN=Sdhd PE=2 SV=1" | Sdhd | 22.6 | 16.976 | 3.9753 |
| B2RYW4 | Mitochondrial ribosomal protein L53 OS=Rattus norvegicus OX=10116 GN=Mrpl53 PE=1 SV=1 | Mrpl53 | 26.1 | 12.735 | 17.635 |
| Q5M849 | Interferon-induced protein 35 OS=Rattus norvegicus OX=10116 GN=Ifi35 PE=1 SV=1 | Ifi35 | 47.6 | 31.764 | 14.035 |
| Q499N5 | "Acyl-CoA synthetase family member 2, mitochondrial OS=Rattus norvegicus OX=10116 GN=Acsf2 PE=2 SV=1" | Acsf2 | 21.8 | 67.886 | 19.167 |
| E9PTB3 | Mitochondrial GTPase 1 OS=Rattus norvegicus OX=10116 GN=Mtg1 PE=1 SV=1 | Mtg1 | 14.7 | 36.561 | 7.904 |
| Q63615 | Vacuolar protein sorting-associated protein 33A OS=Rattus norvegicus OX=10116 GN=Vps33a PE=1 SV=1 | Vps33a | 11.4 | 67.513 | 10.822 |
| Q76MV3 | COX17 cytochrome c oxidase copper chaperone OS=Rattus norvegicus OX=10116 GN=Cox17 PE=1 SV=1 | Cox17 | 84.1 | 6.784 | 141.67 |
| Q924S5 | "Lon protease homolog, mitochondrial OS=Rattus norvegicus OX=10116 GN=Lonp1 PE=2 SV=1" | Lonp1 | 58.6 | 105.79 | 323.31 |
| G3V985 | SCO1 cytochrome c oxidase assembly protein OS=Rattus norvegicus OX=10116 GN=Sco1 PE=1 SV=1 | Sco1 | 21.1 | 31.768 | 52.994 |
| P15650 | "Long-chain specific acyl-CoA dehydrogenase, mitochondrial OS=Rattus norvegicus OX=10116 GN=Acadl PE=1 SV=1" | Acadl | 36.7 | 47.872 | 222.42 |
| Q642E2 | 60S ribosomal protein L28 OS=Rattus norvegicus OX=10116 GN=Rpl28 PE=2 SV=1 | Rpl28 | 31.4 | 15.733 | 206.41 |
| Q5I0D7 | Xaa-Pro dipeptidase OS=Rattus norvegicus OX=10116 GN=Pepd PE=2 SV=1 | Pepd | 21.5 | 54.75 | 28.851 |
| D4A5G8 | Pyruvate dehydrogenase E1 component subunit alpha OS=Rattus norvegicus OX=10116 PE=1 SV=3 | --- | 45.4 | 43.274 | 145.51 |
| Q62834 | Cell division cycle protein 123 homolog OS=Rattus norvegicus OX=10116 GN=Cdc123 PE=1 SV=1 | Cdc123 | 12.2 | 38.812 | 2.9436 |
| D3ZPF2 | Malonyl-CoA-acyl carrier protein transacylase OS=Rattus norvegicus OX=10116 GN=Mcat PE=1 SV=1 | Mcat | 27.6 | 41.832 | 10.414 |
| A0A0G2K6E2 | "Thioredoxin reductase 2, isoform CRA_a OS=Rattus norvegicus OX=10116 GN=Txnrd2 PE=1 SV=1" | Txnrd2 | 24.2 | 56.367 | 8.4929 |
| G3V670 | Methionine aminopeptidase OS=Rattus norvegicus OX=10116 GN=Metap1d PE=1 SV=1 | Metap1d | 13.5 | 37.085 | 3.0349 |
| D3ZPE6 | Mitochondrial ribosomal protein L51 OS=Rattus norvegicus OX=10116 GN=Mrpl51 PE=1 SV=1 | Mrpl51 | 25 | 15.047 | 4.3725 |
| D3Z9K2 | Mitochondrial ribosomal protein L54 OS=Rattus norvegicus OX=10116 GN=Mrpl54 PE=1 SV=1 | Mrpl54 | 49.6 | 15.286 | 52.143 |
| Q4VBH2 | tRNA nucleotidyl transferase 1 OS=Rattus norvegicus OX=10116 GN=Trnt1 PE=1 SV=1 | Trnt1 | 19.4 | 49.821 | 26.469 |
| D3ZQ59 | Nardilysin OS=Rattus norvegicus OX=10116 GN=Nrdc PE=1 SV=1 | Nrdc | 14 | 140.73 | 23.83 |
| Q641Y2 | "NADH dehydrogenase [ubiquinone] iron-sulfur protein 2, mitochondrial OS=Rattus norvegicus OX=10116 GN=Ndufs2 PE=1 SV=1" | Ndufs2 | 46 | 52.561 | 67.514 |
| Q68FX9 | "NAD-dependent protein deacylase sirtuin-5, mitochondrial OS=Rattus norvegicus OX=10116 GN=Sirt5 PE=2 SV=1" | Sirt5 | 46.8 | 34.098 | 18.193 |
| B2RYC9 | Glucosylceramidase OS=Rattus norvegicus OX=10116 GN=Gba PE=1 SV=1 | Gba | 10.7 | 57.491 | 6.3374 |
| Q4V8K5 | BRO1 domain-containing protein BROX OS=Rattus norvegicus OX=10116 GN=Brox PE=2 SV=1 | Brox | 19.7 | 46.191 | 10.555 |
| B2RYW3 | "NADH dehydrogenase (Ubiquinone) 1 beta subcomplex, 9 OS=Rattus norvegicus OX=10116 GN=Ndufb9 PE=1 SV=1" | Ndufb9 | 41.3 | 21.892 | 13.27 |
| G3V734 | "2,4-dienoyl CoA reductase 1, mitochondrial, isoform CRA_a OS=Rattus norvegicus OX=10116 GN=Decr1 PE=1 SV=1" | Decr1 | 32.2 | 36.132 | 56.092 |
| D3ZYT2 | Mitochondrial ribosomal protein S5 OS=Rattus norvegicus OX=10116 GN=Mrps5 PE=1 SV=1 | Mrps5 | 6.2 | 48.136 | 4.2236 |
| P16970 | ATP-binding cassette sub-family D member 3 OS=Rattus norvegicus OX=10116 GN=Abcd3 PE=1 SV=3 | Abcd3 | 10.5 | 75.315 | 4.7888 |
| P20070 | NADH-cytochrome b5 reductase 3 OS=Rattus norvegicus OX=10116 GN=Cyb5r3 PE=1 SV=2 | Cyb5r3 | 60.1 | 34.174 | 323.31 |
| G3V8N8 | Liver regeneration-related protein OS=Rattus norvegicus OX=10116 GN=Slc7a6os PE=1 SV=1 | Slc7a6os | 17.7 | 35.032 | 4.9366 |
| Q68FW7 | "Threonine--tRNA ligase, mitochondrial OS=Rattus norvegicus OX=10116 GN=Tars2 PE=2 SV=1" | Tars2 | 7.5 | 81.671 | 3.9757 |
| A0A0G2K1E2 | Integrin alpha 5 (Mapped) OS=Rattus norvegicus OX=10116 GN=Itga5 PE=1 SV=1 | Itga5 | 17.7 | 119.46 | 112.55 |
| A0A0G2K401 | "Propionyl-CoA carboxylase alpha chain, mitochondrial OS=Rattus norvegicus OX=10116 GN=Pcca PE=1 SV=1" | Pcca | 15.8 | 79.811 | 26.838 |
| Q5RJN0 | NADH dehydrogenase (Ubiquinone) Fe-S protein 7 OS=Rattus norvegicus OX=10116 GN=Ndufs7 PE=1 SV=1 | Ndufs7 | 41.7 | 23.945 | 54.645 |
| G3V8B8 | DnaJ heat shock protein family (Hsp40) member C7 OS=Rattus norvegicus OX=10116 GN=Dnajc7 PE=1 SV=2 | Dnajc7 | 15.1 | 50.1 | 5.2191 |
| D3ZYB6 | DNA-directed RNA polymerase OS=Rattus norvegicus OX=10116 GN=Polrmt PE=3 SV=2 | Polrmt | 5.7 | 135.82 | 1.8238 |
| D3ZIE9 | Delta-1-pyrroline-5-carboxylate synthase OS=Rattus norvegicus OX=10116 GN=Aldh18a1 PE=3 SV=1 | Aldh18a1 | 48.3 | 87.328 | 303.27 |
| Q6P502 | T-complex protein 1 subunit gamma OS=Rattus norvegicus OX=10116 GN=Cct3 PE=1 SV=1 | Cct3 | 60.2 | 60.646 | 323.31 |
| G3V7B5 | Phosphoribosyl pyrophosphate synthase-associated protein 1 OS=Rattus norvegicus OX=10116 GN=Prpsap1 PE=1 SV=1 | Prpsap1 | 24.4 | 42.502 | 7.0226 |
| Q5XIC2 | "Evolutionarily conserved signaling intermediate in Toll pathway, mitochondrial OS=Rattus norvegicus OX=10116 GN=Ecsit PE=1 SV=1" | Ecsit | 31.1 | 49.619 | 19.746 |
| Q7TQ94 | Deaminated glutathione amidase OS=Rattus norvegicus OX=10116 GN=Nit1 PE=2 SV=2 | Nit1 | 44.6 | 36.093 | 117.31 |
| P35284 | Ras-related protein Rab-12 OS=Rattus norvegicus OX=10116 GN=Rab12 PE=1 SV=2 | Rab12 | 32.5 | 27.271 | 11.023 |
| G3V796 | "Acetyl-Coenzyme A dehydrogenase, medium chain OS=Rattus norvegicus OX=10116 GN=Acadm PE=1 SV=1" | Acadm | 25.2 | 46.571 | 22.245 |
| D3ZAW2 | "Phosphatidylserine decarboxylase proenzyme, mitochondrial OS=Rattus norvegicus OX=10116 GN=Pisd PE=1 SV=3" | Pisd | 10.1 | 45.823 | 5.2305 |
| B2GV71 | "Arginine-hydroxylase NDUFAF5, mitochondrial OS=Rattus norvegicus OX=10116 GN=Ndufaf5 PE=2 SV=1" | Ndufaf5 | 10.2 | 38.229 | 2.5105 |
| A0A0G2JV84 | Glutamic--pyruvic transaminase 2 OS=Rattus norvegicus OX=10116 GN=Gpt2 PE=1 SV=1 | Gpt2 | 5.7 | 60.288 | 2.3159 |
| D3ZDP2 | Mitochondrial ribosomal protein L58 OS=Rattus norvegicus OX=10116 GN=Mrpl58 PE=1 SV=1 | Mrpl58 | 22.8 | 23.47 | 6.3854 |
| B5DEJ5 | Eefsec protein OS=Rattus norvegicus OX=10116 GN=Eefsec PE=1 SV=1 | Eefsec | 42.5 | 63.659 | 44.584 |
| A0A0G2JWU3 | Mitochondrial ribosomal protein L1-like OS=Rattus norvegicus OX=10116 GN=LOC100359687 PE=1 SV=1 | LOC100359687 | 23.4 | 34.926 | 10.81 |
| A0A0G2KAN7 | "Glutaminase kidney isoform, mitochondrial OS=Rattus norvegicus OX=10116 GN=Gls PE=1 SV=1" | Gls | 54.5 | 65.838 | 14.639 |
| Q5XI78 | "2-oxoglutarate dehydrogenase, mitochondrial OS=Rattus norvegicus OX=10116 GN=Ogdh PE=1 SV=1" | Ogdh | 44.5 | 116.29 | 323.31 |
| Q6AYA6 | Cytochrome b-245 chaperone 1 OS=Rattus norvegicus OX=10116 GN=Cybc1 PE=1 SV=1 | Cybc1 | 20.3 | 20.894 | 5.7505 |
| D3ZBL5 | "Type I inositol 3,4-bisphosphate 4-phosphatase OS=Rattus norvegicus OX=10116 GN=Inpp4a PE=1 SV=3" | Inpp4a | 6.7 | 105.6 | 5.7827 |
| D4A8N1 | "Dolichyl-phosphate mannosyltransferase subunit 1, catalytic OS=Rattus norvegicus OX=10116 GN=Dpm1 PE=1 SV=1" | Dpm1 | 40.8 | 29.249 | 37.077 |
| D3ZZ20 | AFG3(ATPase family gene 3)-like 1 (S. cerevisiae) OS=Rattus norvegicus OX=10116 GN=Afg3l1 PE=1 SV=2 | Afg3l1 | 20.4 | 87.202 | 19.526 |
| D3ZI43 | Peptidyl-tRNA hydrolase 1 homolog OS=Rattus norvegicus OX=10116 GN=Ptrh1 PE=1 SV=1 | Ptrh1 | 43.1 | 22.071 | 32.301 |
| A0A0G2JUQ8 | Ubiquitin carboxyl-terminal hydrolase 48 OS=Rattus norvegicus OX=10116 GN=Usp48 PE=1 SV=1 | Usp48 | 13.7 | 121.94 | 14.254 |
| F7FG31 | C-terminal-binding protein 1 OS=Rattus norvegicus OX=10116 GN=Ctbp1 PE=1 SV=1 | Ctbp1 | 28.5 | 43.847 | 9.5836 |
| A0A140TAE6 | "Enoyl-[acyl-carrier-protein] reductase, mitochondrial OS=Rattus norvegicus OX=10116 GN=Mecr PE=1 SV=1" | Mecr | 24.7 | 40.334 | 7.7353 |
| F6QBA3 | Perilipin OS=Rattus norvegicus OX=10116 GN=Plin2 PE=1 SV=1 | Plin2 | 35.6 | 46.184 | 83.606 |
| G3V7I0 | Peroxiredoxin 3 OS=Rattus norvegicus OX=10116 GN=Prdx3 PE=1 SV=1 | Prdx3 | 40.5 | 28.299 | 317.05 |
| A0A0G2K9I7 | Intermediate filament family orphan 2 OS=Rattus norvegicus OX=10116 GN=Aldh4a1 PE=1 SV=1 | Aldh4a1 | 39.2 | 57.787 | 236.88 |
| A0A0G2K277 | "Enoyl-CoA delta isomerase 2, mitochondrial OS=Rattus norvegicus OX=10116 GN=Eci3 PE=1 SV=1" | Eci3 | 25.9 | 42.324 | 4.5134 |
| D4ABM5 | Mitochondrial ribosomal protein S34 OS=Rattus norvegicus OX=10116 GN=Mrps34 PE=1 SV=1 | Mrps34 | 39 | 25.799 | 47.01 |
| P12007 | "Isovaleryl-CoA dehydrogenase, mitochondrial OS=Rattus norvegicus OX=10116 GN=Ivd PE=1 SV=2" | Ivd | 43.6 | 46.435 | 186.43 |
| Q4V7F3 | "Probable tRNA N6-adenosine threonylcarbamoyltransferase, mitochondrial OS=Rattus norvegicus OX=10116 GN=Osgepl1 PE=2 SV=1" | Osgepl1 | 13.3 | 44.851 | 3.5488 |
| D3ZQX3 | Mitochondrial ribosomal protein S12 OS=Rattus norvegicus OX=10116 GN=Mrps12 PE=1 SV=1 | Mrps12 | 18 | 15.441 | 2.5997 |
| D3ZZA8 | "SEC24 homolog A, COPII coat complex component OS=Rattus norvegicus OX=10116 GN=Sec24a PE=1 SV=1" | Sec24a | 17.8 | 118.87 | 14.834 |
| Q5PQN9 | "39S ribosomal protein L38, mitochondrial OS=Rattus norvegicus OX=10116 GN=Mrpl38 PE=2 SV=2" | Mrpl38 | 13.7 | 44.837 | 5.6409 |
| A0A0G2K8T0 | Acid ceramidase OS=Rattus norvegicus OX=10116 GN=Asah1 PE=1 SV=1 | Asah1 | 31.8 | 48.282 | 50.237 |
| A0A0G2KB26 | "Limitrin, isoform CRA_b OS=Rattus norvegicus OX=10116 GN=Mxra8 PE=4 SV=1" | Mxra8 | 29.2 | 49.715 | 19.124 |
| Q63159 | "Ubiquinone biosynthesis O-methyltransferase, mitochondrial OS=Rattus norvegicus OX=10116 GN=Coq3 PE=2 SV=2" | Coq3 | 9 | 38.708 | 1.9628 |
| Q5XIH3 | "NADH dehydrogenase [ubiquinone] flavoprotein 1, mitochondrial OS=Rattus norvegicus OX=10116 GN=Ndufv1 PE=1 SV=1" | Ndufv1 | 44.6 | 50.73 | 186.52 |
| D3ZJZ1 | Autophagy protein 5 OS=Rattus norvegicus OX=10116 GN=Atg5 PE=1 SV=3 | Atg5 | 13.5 | 27.704 | 2.7048 |
| G3V7Y3 | "ATP synthase subunit delta, mitochondrial OS=Rattus norvegicus OX=10116 GN=Atp5f1d PE=1 SV=1" | Atp5f1d | 37.5 | 17.563 | 237.83 |
| Q32Q54 | "RCG37273, isoform CRA_a OS=Rattus norvegicus OX=10116 GN=Uqcc1 PE=1 SV=1" | Uqcc1 | 13.4 | 34.546 | 7.5403 |
| Q5M9H2 | "Acyl-Coenzyme A dehydrogenase, very long chain OS=Rattus norvegicus OX=10116 GN=Acadvl PE=1 SV=1" | Acadvl | 46 | 70.821 | 322.01 |
| Q5U2R6 | Putative monooxygenase p33MONOX OS=Rattus norvegicus OX=10116 GN=P33monox PE=1 SV=1 | P33monox | 15.5 | 32.797 | 2.4421 |
| F1LQZ9 | Microtubule-associated protein 6 OS=Rattus norvegicus OX=10116 GN=Map6 PE=1 SV=3 | Map6 | 17.8 | 100.44 | 16.375 |
| F1M581 | Heparanase OS=Rattus norvegicus OX=10116 GN=Hpse PE=4 SV=3 | Hpse | 8.6 | 60.877 | 3.3983 |
| A0A0G2K7L0 | "28S ribosomal protein S10, mitochondrial OS=Rattus norvegicus OX=10116 GN=Mrps10 PE=1 SV=1" | Mrps10 | 24.9 | 23.065 | 3.8091 |
| F7FFR1 | "Arginyl-tRNA synthetase 2, mitochondrial OS=Rattus norvegicus OX=10116 GN=Rars2 PE=1 SV=1" | Rars2 | 14.4 | 65.228 | 7.5679 |
| P85834 | "Elongation factor Tu, mitochondrial OS=Rattus norvegicus OX=10116 GN=Tufm PE=1 SV=1" | Tufm | 56.6 | 49.522 | 323.31 |
| F7EWS7 | Apolipoprotein B mRNA editing enzyme catalytic subunit 3B OS=Rattus norvegicus OX=10116 GN=Apobec3 PE=4 SV=1 | Apobec3 | 15.9 | 47.856 | 5.4901 |
| Q5RK00 | "39S ribosomal protein L46, mitochondrial OS=Rattus norvegicus OX=10116 GN=Mrpl46 PE=2 SV=1" | Mrpl46 | 43.3 | 31.673 | 111.64 |
| Q641W2 | "UPF0160 protein MYG1, mitochondrial OS=Rattus norvegicus OX=10116 GN=Myg1 PE=1 SV=1" | Myg1 | 25.7 | 42.888 | 10.972 |
| D3ZVS2 | L-2-hydroxyglutarate dehydrogenase OS=Rattus norvegicus OX=10116 GN=L2hgdh PE=1 SV=1 | L2hgdh | 32.8 | 50.732 | 20.23 |
| P18886 | "Carnitine O-palmitoyltransferase 2, mitochondrial OS=Rattus norvegicus OX=10116 GN=Cpt2 PE=1 SV=1" | Cpt2 | 40.9 | 74.109 | 63.773 |
| D3ZY47 | Similar to RIKEN cDNA 2310022B05 OS=Rattus norvegicus OX=10116 GN=RGD1559896 PE=1 SV=1 | RGD1559896 | 29 | 35.668 | 15.229 |
| A0A0G2JT47 | Sorting nexin-16 OS=Rattus norvegicus OX=10116 GN=Snx16 PE=1 SV=1 | Snx16 | 8.4 | 40.127 | 3.4852 |
| G3V9W4 | "RCG59854, isoform CRA_b OS=Rattus norvegicus OX=10116 GN=Xpnpep3 PE=1 SV=1" | Xpnpep3 | 18.8 | 56.484 | 6.9362 |
| G3V913 | Heat shock 27kDa protein 1 OS=Rattus norvegicus OX=10116 GN=Hspb1 PE=1 SV=1 | Hspb1 | 75.7 | 22.807 | 55.927 |
| Q5XIM7 | Lysine--tRNA ligase OS=Rattus norvegicus OX=10116 GN=Kars PE=1 SV=1 | Kars | 36.6 | 71.622 | 253.77 |
| F1M5H6 | "Tumor protein p53-binding protein, 2 OS=Rattus norvegicus OX=10116 GN=Tp53bp2 PE=1 SV=2" | Tp53bp2 | 16.1 | 125.31 | 15.213 |
| D4AC36 | Eukaryotic translation initiation factor 3 subunit F OS=Rattus norvegicus OX=10116 GN=Eif3f PE=1 SV=3 | Eif3f | 29.1 | 37.997 | 117.34 |
| B5DFN3 | Ubiquinol-cytochrome-c reductase complex assembly factor 2 OS=Rattus norvegicus OX=10116 GN=Uqcc2 PE=2 SV=1 | Uqcc2 | 46.3 | 16.33 | 8.3613 |
| D3ZT90 | Glutaryl-CoA dehydrogenase OS=Rattus norvegicus OX=10116 GN=Gcdh PE=1 SV=1 | Gcdh | 27.1 | 49.713 | 65.522 |
| G3V940 | Coronin OS=Rattus norvegicus OX=10116 GN=Coro1b PE=1 SV=1 | Coro1b | 31 | 53.938 | 285.43 |
| Q5XJW2 | Growth arrest and DNA damage-inducible proteins-interacting protein 1 OS=Rattus norvegicus OX=10116 GN=Gadd45gip1 PE=2 SV=2 | Gadd45gip1 | 33.3 | 26.467 | 50.59 |
| D4A565 | "NADH dehydrogenase (Ubiquinone) 1 beta subcomplex, 5 (Predicted), isoform CRA_b OS=Rattus norvegicus OX=10116 GN=Ndufb5 PE=1 SV=1" | Ndufb5 | 18 | 21.664 | 3.7208 |
| F7FHF7 | Mitofusin-2 OS=Rattus norvegicus OX=10116 GN=LOC100911485 PE=1 SV=1 | LOC100911485 | 21.1 | 86.812 | 19.112 |
| Q6PEC0 | Bis(5'-nucleosyl)-tetraphosphatase [asymmetrical] OS=Rattus norvegicus OX=10116 GN=Nudt2 PE=2 SV=3 | Nudt2 | 48.3 | 16.929 | 6.079 |
| Q5I0K8 | "28S ribosomal protein S7, mitochondrial OS=Rattus norvegicus OX=10116 GN=Mrps7 PE=2 SV=2" | Mrps7 | 42.6 | 28.197 | 111.01 |
| Q4KLN6 | Ribonucleoside-diphosphate reductase subunit M2 OS=Rattus norvegicus OX=10116 GN=Rrm2 PE=2 SV=1 | Rrm2 | 26.2 | 45.038 | 13.976 |
| M0RC99 | Ras-related protein Rab-5A OS=Rattus norvegicus OX=10116 GN=Rab5a PE=2 SV=1 | Rab5a | 48.4 | 23.624 | 17.555 |
| D4A099 | 5-methylcytosine rRNA methyltransferase NSUN4 OS=Rattus norvegicus OX=10116 GN=Nsun4 PE=1 SV=1 | Nsun4 | 9.7 | 42.612 | 4.3021 |
| F1LRY5 | RCG45398 OS=Rattus norvegicus OX=10116 GN=Sardh PE=1 SV=2 | Sardh | 30.7 | 101.41 | 32.231 |
| Q6IMX8 | Acyl-CoA thioesterase 2 OS=Rattus norvegicus OX=10116 GN=Acot2 PE=1 SV=1 | Acot2 | 17 | 49.628 | 4.0968 |
| D4A2F6 | NHL repeat-containing 3 OS=Rattus norvegicus OX=10116 GN=Nhlrc3 PE=1 SV=2 | Nhlrc3 | 27.5 | 42.636 | 20.758 |
| A0A0G2K2N0 | Tyrosine-protein phosphatase non-receptor type OS=Rattus norvegicus OX=10116 GN=Ptpn14 PE=1 SV=1 | Ptpn14 | 3 | 134.78 | 33.586 |
| Q9WVK7 | "Hydroxyacyl-coenzyme A dehydrogenase, mitochondrial OS=Rattus norvegicus OX=10116 GN=Hadh PE=2 SV=1" | Hadh | 48.4 | 34.447 | 155.47 |
| Q5RK30 | Ribosome maturation protein SBDS OS=Rattus norvegicus OX=10116 GN=Sbds PE=2 SV=1 | Sbds | 27.6 | 28.752 | 73.232 |
| A0A0G2K642 | "3-ketoacyl-CoA thiolase, mitochondrial OS=Rattus norvegicus OX=10116 GN=Acaa2 PE=1 SV=1" | Acaa2 | 71.7 | 41.753 | 310.78 |
| A0A0G2K0V3 | "3'(2'),5'-bisphosphate nucleotidase 1 OS=Rattus norvegicus OX=10116 GN=Bpnt1 PE=1 SV=1" | Bpnt1 | 21.7 | 35.051 | 10.588 |
| D3ZNY3 | "Methylmalonic aciduria (Cobalamin deficiency) cblA type (Predicted), isoform CRA_a OS=Rattus norvegicus OX=10116 GN=Mmaa PE=1 SV=1" | Mmaa | 12.8 | 46.329 | 7.2042 |
| Q6AYG5 | Ethylmalonyl-CoA decarboxylase OS=Rattus norvegicus OX=10116 GN=Echdc1 PE=1 SV=1 | Echdc1 | 17.4 | 32.63 | 7.3401 |
| A0A0A0MXZ0 | "Iron-sulfur cluster assembly 1 homolog, mitochondrial OS=Rattus norvegicus OX=10116 GN=Isca1 PE=1 SV=1" | Isca1 | 24 | 14.122 | 2.484 |
| D3ZC46 | Transcription factor 25 OS=Rattus norvegicus OX=10116 GN=Tcf25 PE=1 SV=2 | Tcf25 | 16.3 | 75.32 | 7.1404 |
| E9PTG8 | Serine/threonine-protein kinase 10 OS=Rattus norvegicus OX=10116 GN=Stk10 PE=1 SV=1 | Stk10 | 14.6 | 111.88 | 20.483 |
| Q561S0 | "NADH dehydrogenase [ubiquinone] 1 alpha subcomplex subunit 10, mitochondrial OS=Rattus norvegicus OX=10116 GN=Ndufa10 PE=1 SV=1" | Ndufa10 | 43.4 | 40.493 | 55.498 |
| D3ZXA6 | Pyruvate dehydrogenase phosphatase regulatory subunit OS=Rattus norvegicus OX=10116 GN=Pdpr PE=1 SV=1 | Pdpr | 4.8 | 98.818 | 4.0802 |
| G3V7J0 | "Aldehyde dehydrogenase family 6, subfamily A1, isoform CRA_b OS=Rattus norvegicus OX=10116 GN=Aldh6a1 PE=1 SV=1" | Aldh6a1 | 30.7 | 57.747 | 39.269 |
| Q6Q0N3 | 5'-nucleotidase domain-containing protein 2 OS=Rattus norvegicus OX=10116 GN=Nt5dc2 PE=2 SV=2 | Nt5dc2 | 41.8 | 63.652 | 130.46 |
| Q63486 | Ras-related GTP-binding protein A OS=Rattus norvegicus OX=10116 GN=Rraga PE=1 SV=1 | Rraga | 8.9 | 36.566 | 6.0716 |
| D4A520 | PTC7 protein phosphatase homolog OS=Rattus norvegicus OX=10116 GN=Pptc7 PE=1 SV=1 | Pptc7 | 16.9 | 32.847 | 4.0174 |
| D3ZX69 | "39S ribosomal protein L10, mitochondrial OS=Rattus norvegicus OX=10116 GN=Mrpl10 PE=1 SV=1" | Mrpl10 | 34.7 | 29.465 | 55.79 |
| M0R6J0 | Mitochondrial ribosomal protein L39 OS=Rattus norvegicus OX=10116 GN=Mrpl39 PE=1 SV=2 | Mrpl39 | 29.5 | 38.374 | 18.269 |
| G3V6F5 | ElaC homolog 2 (E. coli) OS=Rattus norvegicus OX=10116 GN=Elac2 PE=1 SV=1 | Elac2 | 18.3 | 92.33 | 17.523 |
| B2GUZ2 | Spindle and kinetochore-associated protein 3 OS=Rattus norvegicus OX=10116 GN=Ska3 PE=2 SV=1 | Ska3 | 11.5 | 46.352 | 2.7067 |
| G3V717 | Merlin OS=Rattus norvegicus OX=10116 GN=Nf2 PE=1 SV=1 | Nf2 | 11.3 | 69.203 | 3.9204 |
| D3ZFQ3 | Nucleotide-binding protein-like OS=Rattus norvegicus OX=10116 GN=Nubpl PE=1 SV=1 | Nubpl | 23.5 | 34.082 | 7.575 |
| F1M049 | Ataxin 2 OS=Rattus norvegicus OX=10116 GN=Atxn2 PE=1 SV=3 | Atxn2 | 19.2 | 117.3 | 67.122 |
| D3ZC63 | Cytidine/uridine monophosphate kinase 2 OS=Rattus norvegicus OX=10116 GN=Cmpk2 PE=1 SV=3 | Cmpk2 | 11.2 | 50.113 | 5.9152 |
| Q498T4 | "39S ribosomal protein L2, mitochondrial OS=Rattus norvegicus OX=10116 GN=Mrpl2 PE=2 SV=1" | Mrpl2 | 13.2 | 32.999 | 12.9 |
| B1WC61 | Acad9 protein OS=Rattus norvegicus OX=10116 GN=Acad9 PE=1 SV=1 | Acad9 | 44.6 | 68.842 | 274.17 |
| Q66HK3 | Prostaglandin G/H synthase 1 OS=Rattus norvegicus OX=10116 GN=Ptgs1 PE=1 SV=1 | Ptgs1 | 7.5 | 69.046 | 3.9464 |
| D4A978 | Phosphoinositide phospholipase C OS=Rattus norvegicus OX=10116 GN=Plcd3 PE=1 SV=1 | Plcd3 | 17.6 | 89.47 | 23.476 |
| Q64428 | "Trifunctional enzyme subunit alpha, mitochondrial OS=Rattus norvegicus OX=10116 GN=Hadha PE=1 SV=2" | Hadha | 54.5 | 82.664 | 323.31 |
| F1M9C9 | "Histidyl-tRNA synthetase 2, mitochondrial OS=Rattus norvegicus OX=10116 GN=Hars2 PE=1 SV=1" | Hars2 | 13.2 | 57.327 | 5.2799 |
| G3V6P2 | "Dihydrolipoamide S-succinyltransferase (E2 component of 2-oxo-glutarate complex), isoform CRA_a OS=Rattus norvegicus OX=10116 GN=Dlst PE=1 SV=1" | Dlst | 21.1 | 48.899 | 230.99 |
| F1LXA0 | NADH dehydrogenase [ubiquinone] 1 alpha subcomplex subunit 12 OS=Rattus norvegicus OX=10116 GN=Ndufa12 PE=1 SV=2 | Ndufa12 | 20.7 | 17.177 | 14.568 |
| A0A0A0MY00 | "Short/branched chain-specific acyl-CoA dehydrogenase, mitochondrial OS=Rattus norvegicus OX=10116 GN=Acadsb PE=1 SV=2" | Acadsb | 12.4 | 52.042 | 9.1648 |
| P29117 | "Peptidyl-prolyl cis-trans isomerase F, mitochondrial OS=Rattus norvegicus OX=10116 GN=Ppif PE=1 SV=2" | Ppif | 29.1 | 21.81 | 34.268 |
| B2GV06 | "Succinyl-CoA:3-ketoacid coenzyme A transferase 1, mitochondrial OS=Rattus norvegicus OX=10116 GN=Oxct1 PE=1 SV=1" | Oxct1 | 48.5 | 56.203 | 323.31 |
| Q7M733 | Hermansky-Pudlak syndrome 6 protein homolog OS=Rattus norvegicus OX=10116 GN=Hps6 PE=1 SV=1 | Hps6 | 7.3 | 87.461 | 4.7345 |
| D4A040 | Mitochondrial ribosomal protein S11 OS=Rattus norvegicus OX=10116 GN=Mrps11 PE=1 SV=1 | Mrps11 | 35.1 | 20.168 | 12.317 |
| E9PSL1 | Sperm-associated antigen 5 OS=Rattus norvegicus OX=10116 GN=Spag5 PE=4 SV=2 | Spag5 | 4.1 | 132.44 | 4.9297 |
| P10818 | "Cytochrome c oxidase subunit 6A1, mitochondrial OS=Rattus norvegicus OX=10116 GN=Cox6a1 PE=1 SV=2" | Cox6a1 | 42.3 | 12.301 | 49.194 |
| A0A0G2JXF9 | Host cell factor 2 OS=Rattus norvegicus OX=10116 GN=Hcfc2 PE=4 SV=1 | Hcfc2 | 14.2 | 79.018 | 8.4864 |
| A0A0G2K167 | Regulator of microtubule dynamics protein 1 OS=Rattus norvegicus OX=10116 GN=Rmdn1 PE=1 SV=1 | Rmdn1 | 11.9 | 36.795 | 4.4405 |
| P83565 | "39S ribosomal protein L40, mitochondrial OS=Rattus norvegicus OX=10116 GN=Mrpl40 PE=1 SV=2" | Mrpl40 | 42.7 | 24.397 | 25.75 |
| D3ZCZ9 | "NADH dehydrogenase [ubiquinone] iron-sulfur protein 6, mitochondrial OS=Rattus norvegicus OX=10116 GN=LOC100912599 PE=1 SV=1" | LOC100912599 | 64.7 | 13.04 | 18.23 |
| Q6AY19 | "Atypical kinase COQ8B, mitochondrial OS=Rattus norvegicus OX=10116 GN=Coq8b PE=1 SV=1" | Coq8b | 19.3 | 58.904 | 9.2376 |
| Q68FT1 | "Ubiquinone biosynthesis protein COQ9, mitochondrial OS=Rattus norvegicus OX=10116 GN=Coq9 PE=1 SV=2" | Coq9 | 30.8 | 35.145 | 84.978 |
| D3ZEH2 | FAD-dependent oxidoreductase domain containing 1 OS=Rattus norvegicus OX=10116 GN=Foxred1 PE=1 SV=1 | Foxred1 | 10.9 | 54.229 | 21.144 |
| P11348 | Dihydropteridine reductase OS=Rattus norvegicus OX=10116 GN=Qdpr PE=1 SV=1 | Qdpr | 49.4 | 25.552 | 225.95 |
| Q4V8J7 | Spindlin-1 OS=Rattus norvegicus OX=10116 GN=Spin1 PE=2 SV=1 | Spin1 | 13.4 | 29.628 | 5.796 |
| A9UMV2 | Mitochondrial ribosomal protein S24 OS=Rattus norvegicus OX=10116 GN=Mrps24 PE=1 SV=1 | Mrps24 | 28.7 | 18.742 | 10.804 |
| A0A0H2UHY0 | "Valine--tRNA ligase, mitochondrial OS=Rattus norvegicus OX=10116 GN=Vars2 PE=1 SV=1" | Vars2 | 7 | 118.37 | 5.3423 |
| Q8VID1 | Dehydrogenase/reductase SDR family member 4 OS=Rattus norvegicus OX=10116 GN=Dhrs4 PE=2 SV=2 | Dhrs4 | 18.6 | 29.821 | 6.1753 |
| P50411 | Protein phosphatase inhibitor 2 OS=Rattus norvegicus OX=10116 GN=Ppp1r2 PE=1 SV=2 | Ppp1r2 | 46.3 | 23.071 | 9.7153 |
| A0A0G2K824 | Mannose-1-phosphate guanyltransferase alpha OS=Rattus norvegicus OX=10116 GN=Gmppa PE=1 SV=1 | Gmppa | 7.1 | 46.648 | 4.3532 |
| Q4QQV8 | Charged multivesicular body protein 5 OS=Rattus norvegicus OX=10116 GN=Chmp5 PE=2 SV=1 | Chmp5 | 30.6 | 24.575 | 33.519 |
| B0BNJ4 | "ETHE1, persulfide dioxygenase OS=Rattus norvegicus OX=10116 GN=Ethe1 PE=1 SV=1" | Ethe1 | 66.5 | 27.676 | 103.12 |
| Q66H47 | "39S ribosomal protein L24, mitochondrial OS=Rattus norvegicus OX=10116 GN=Mrpl24 PE=2 SV=1" | Mrpl24 | 24.1 | 25 | 3.6429 |
| P19511 | "ATP synthase F(0) complex subunit B1, mitochondrial OS=Rattus norvegicus OX=10116 GN=Atp5pb PE=1 SV=1" | Atp5pb | 44.9 | 28.868 | 286.2 |
| F1LMQ3 | "Proteasome 26S subunit, non-ATPase 8 OS=Rattus norvegicus OX=10116 GN=Psmd8 PE=1 SV=1" | Psmd8 | 21.5 | 39.877 | 54.332 |
| A0A0A0MY43 | Activating signal cointegrator 1 complex subunit 3 OS=Rattus norvegicus OX=10116 GN=Ascc3 PE=1 SV=1 | Ascc3 | 13.8 | 250.53 | 40.226 |
| B5DF65 | Biliverdin reductase B OS=Rattus norvegicus OX=10116 GN=Blvrb PE=1 SV=1 | Blvrb | 65.5 | 22.094 | 87.314 |
| Q5BJP3 | Ubiquitin-fold modifier 1 OS=Rattus norvegicus OX=10116 GN=Ufm1 PE=3 SV=1 | Ufm1 | 58.8 | 9.1175 | 20.012 |
| D4A3V2 | NADH dehydrogenase [ubiquinone] 1 alpha subcomplex subunit 6 OS=Rattus norvegicus OX=10116 GN=Ndufa6 PE=1 SV=1 | Ndufa6 | 33.1 | 15.224 | 16.801 |
| Q5I0K5 | "Mycophenolic acid acyl-glucuronide esterase, mitochondrial OS=Rattus norvegicus OX=10116 GN=Abhd10 PE=1 SV=2" | Abhd10 | 20.5 | 33.152 | 12.441 |
| Q920L2 | "Succinate dehydrogenase [ubiquinone] flavoprotein subunit, mitochondrial OS=Rattus norvegicus OX=10116 GN=Sdha PE=1 SV=1" | Sdha | 56.7 | 71.614 | 323.31 |
| P20909 | Collagen alpha-1(XI) chain OS=Rattus norvegicus OX=10116 GN=Col11a1 PE=1 SV=2 | Col11a1 | 5 | 181.02 | 5.2503 |
| D4A5Z0 | Hexosyltransferase OS=Rattus norvegicus OX=10116 GN=Csgalnact2 PE=3 SV=1 | Csgalnact2 | 7.9 | 62.654 | 1.5892 |
| F6Q5K7 | Mitochondrial ribosomal protein S18B OS=Rattus norvegicus OX=10116 GN=Mrps18b PE=1 SV=1 | Mrps18b | 44.7 | 29.159 | 73.96 |
| D3ZTW7 | ATP synthase mitochondrial F1 complex assembly factor 2 OS=Rattus norvegicus OX=10116 GN=Atpaf2 PE=1 SV=1 | Atpaf2 | 52 | 34.192 | 30.503 |
| P21775 | "3-ketoacyl-CoA thiolase A, peroxisomal OS=Rattus norvegicus OX=10116 GN=Acaa1a PE=1 SV=2" | Acaa1a | 46.7 | 43.833 | 18.892 |
| Q5I0D1 | Glyoxalase domain-containing protein 4 OS=Rattus norvegicus OX=10116 GN=Glod4 PE=1 SV=1 | Glod4 | 45.6 | 33.267 | 63.026 |
| P51146 | Ras-related protein Rab-4B OS=Rattus norvegicus OX=10116 GN=Rab4b PE=2 SV=1 | Rab4b | 34.7 | 23.629 | 7.938 |
| B2RYT7 | Haloacid dehalogenase-like hydrolase domain-containing 3 OS=Rattus norvegicus OX=10116 GN=Hdhd3 PE=1 SV=1 | Hdhd3 | 14.3 | 27.794 | 3.4121 |
| F1MAK1 | Formin-binding protein 4 OS=Rattus norvegicus OX=10116 GN=Fnbp4 PE=1 SV=3 | Fnbp4 | 13.2 | 113.09 | 11.173 |
| Q5U2R4 | tRNA methyltransferase 10 homolog C OS=Rattus norvegicus OX=10116 GN=Trmt10c PE=2 SV=1 | Trmt10c | 15.9 | 48.292 | 23.732 |
| F1LQJ7 | Phosphoenolpyruvate carboxykinase 2 (mitochondrial) OS=Rattus norvegicus OX=10116 GN=Pck2 PE=1 SV=2 | Pck2 | 23.9 | 71.206 | 36.907 |
| D3ZFJ6 | "Lactamase, beta OS=Rattus norvegicus OX=10116 GN=Lactb PE=1 SV=1" | Lactb | 21.5 | 60.42 | 27.348 |
| Q5U2V5 | Cardiolipin synthase (CMP-forming) OS=Rattus norvegicus OX=10116 GN=Crls1 PE=2 SV=1 | Crls1 | 13.2 | 32.628 | 3.412 |
| A0A0G2K2Q2 | Glycine C-acetyltransferase OS=Rattus norvegicus OX=10116 GN=Gcat PE=1 SV=1 | Gcat | 37.3 | 45.197 | 38.951 |
| A0A0G2K0T5 | "Calcium uptake protein 1, mitochondrial OS=Rattus norvegicus OX=10116 GN=Micu1 PE=1 SV=1" | Micu1 | 35.1 | 54.817 | 81.158 |
| D3ZJY1 | Mitochondrial ribosomal protein L28 OS=Rattus norvegicus OX=10116 GN=Mrpl28 PE=1 SV=1 | Mrpl28 | 21.8 | 30.236 | 5.7744 |
| B2GV15 | Dihydrolipoamide acetyltransferase component of pyruvate dehydrogenase complex OS=Rattus norvegicus OX=10116 GN=Dbt PE=1 SV=1 | Dbt | 25.5 | 53.273 | 20.223 |
| Q4QQV3 | Protein FAM162A OS=Rattus norvegicus OX=10116 GN=Fam162a PE=2 SV=1 | Fam162a | 34.2 | 17.827 | 38.573 |
| Q64350 | Translation initiation factor eIF-2B subunit epsilon OS=Rattus norvegicus OX=10116 GN=Eif2b5 PE=1 SV=2 | Eif2b5 | 17.7 | 80.205 | 14.467 |
| D3ZLE6 | "RT1 class I, CE7 OS=Rattus norvegicus OX=10116 GN=RT1-CE7 PE=2 SV=3" | RT1-CE7 | 11.7 | 39.347 | 14.869 |
| A0A0G2K528 | Syntaxin 16 OS=Rattus norvegicus OX=10116 GN=Stx16 PE=1 SV=1 | Stx16 | 30.7 | 35.441 | 9.2028 |
| D4AAE9 | CDGSH iron sulfur domain 2 OS=Rattus norvegicus OX=10116 GN=Cisd2 PE=1 SV=1 | Cisd2 | 29.6 | 15.293 | 36.511 |
| Q68FY0 | "Cytochrome b-c1 complex subunit 1, mitochondrial OS=Rattus norvegicus OX=10116 GN=Uqcrc1 PE=1 SV=1" | Uqcrc1 | 42.5 | 52.848 | 323.31 |
| D4AB01 | "Histidine triad nucleotide binding protein 2 (Predicted), isoform CRA_a OS=Rattus norvegicus OX=10116 GN=Hint2 PE=1 SV=1" | Hint2 | 42.3 | 17.38 | 25.939 |
| P61354 | 60S ribosomal protein L27 OS=Rattus norvegicus OX=10116 GN=Rpl27 PE=2 SV=2 | Rpl27 | 43.4 | 15.798 | 323.31 |
| A0A0A0MXZ3 | "D-2-hydroxyglutarate dehydrogenase, mitochondrial OS=Rattus norvegicus OX=10116 GN=D2hgdh PE=1 SV=1" | D2hgdh | 13.9 | 57.74 | 8.0816 |
| G3V624 | Coronin OS=Rattus norvegicus OX=10116 GN=Coro1c PE=1 SV=1 | Coro1c | 42.4 | 53.177 | 323.31 |
| A0A0G2K4T8 | Mitochondrial intermediate peptidase OS=Rattus norvegicus OX=10116 GN=Mipep PE=1 SV=1 | Mipep | 17.9 | 80.875 | 14.939 |
| F1LU71 | "Methylglutaconyl-CoA hydratase, mitochondrial OS=Rattus norvegicus OX=10116 GN=Auh PE=1 SV=2" | Auh | 13.7 | 33.341 | 26.098 |
| D3ZME7 | HscB mitochondrial iron-sulfur cluster co-chaperone OS=Rattus norvegicus OX=10116 GN=Hscb PE=1 SV=1 | Hscb | 26.5 | 27.065 | 4.6399 |
| A0A096MJR6 | Poly [ADP-ribose] polymerase (Fragment) OS=Rattus norvegicus OX=10116 PE=1 SV=1 | --- | 8.3 | 89.891 | 4.8383 |
| A0A096MKA7 | Protein ABHD16A (Fragment) OS=Rattus norvegicus OX=10116 GN=Abhd16a PE=1 SV=1 | Abhd16a | 26.4 | 13.962 | 2.3735 |
| A0A0G2JSH2 | "3-hydroxybutyrate dehydrogenase, type 1, isoform CRA_a OS=Rattus norvegicus OX=10116 GN=Bdh1 PE=1 SV=1" | Bdh1 | 10.8 | 38.333 | 1.8533 |
| A0A0G2JSL7 | RCG39005 OS=Rattus norvegicus OX=10116 GN=Pdp2 PE=1 SV=1 | Pdp2 | 10.2 | 59.686 | 2.0519 |
| A0A0G2JZF6 | NADH:ubiquinone oxidoreductase complex assembly factor 2 OS=Rattus norvegicus OX=10116 GN=Ndufaf2 PE=1 SV=1 | Ndufaf2 | 34.3 | 19.554 | 5.1175 |
| A0A0G2K099 | Uncharacterized protein OS=Rattus norvegicus OX=10116 PE=1 SV=1 | --- | 46.5 | 54.295 | 4.1199 |
| A0A0G2K7D7 | "Asparaginyl-tRNA synthetase 2, mitochondrial OS=Rattus norvegicus OX=10116 GN=Nars2 PE=1 SV=1" | Nars2 | 9.5 | 49.758 | 2.8731 |
| A0A0G2K8Q8 | "Ubiquinol-cytochrome c reductase, complex III subunit X OS=Rattus norvegicus OX=10116 GN=Uqcr10 PE=1 SV=1" | Uqcr10 | 27.4 | 7.099 | 87.859 |
| A0A0G2K9Q1 | Golgi phosphoprotein 3-like OS=Rattus norvegicus OX=10116 GN=Golph3l PE=1 SV=1 | Golph3l | 15 | 33.961 | 2.1293 |
| A0A0H2UHL2 | "Glutaminase liver isoform, mitochondrial OS=Rattus norvegicus OX=10116 GN=Gls2 PE=1 SV=1" | Gls2 | 7.4 | 63.888 | 1.7158 |
| A0A0H2UI05 | Cilia- and flagella-associated protein 20 OS=Rattus norvegicus OX=10116 GN=Cfap20 PE=4 SV=1 | Cfap20 | 8.8 | 22.748 | 2.9393 |
| A4F267 | Mitochondrial import receptor subunit TOM40B OS=Rattus norvegicus OX=10116 GN=Tomm40l PE=1 SV=1 | Tomm40l | 12.3 | 34.048 | 2.5409 |
| B0BNE3 | Trafficking protein particle complex subunit 5 OS=Rattus norvegicus OX=10116 GN=Trappc5 PE=1 SV=1 | Trappc5 | 21.3 | 20.795 | 2.5163 |
| B0BNE9 | LOC684274 protein OS=Rattus norvegicus OX=10116 GN=Mtif3 PE=1 SV=1 | Mtif3 | 13.4 | 31.526 | 2.0585 |
| B0K032 | "Protein tyrosine phosphatase 4a3 (Predicted), isoform CRA_a OS=Rattus norvegicus OX=10116 GN=Ptp4a3 PE=2 SV=1" | Ptp4a3 | 11 | 19.652 | 1.5115 |
| B1H245 | BCL tumor suppressor 7B OS=Rattus norvegicus OX=10116 GN=Bcl7b PE=1 SV=1 | Bcl7b | 11.9 | 22.252 | 1.9149 |
| B2GV62 | Mitochondrial ribosomal protein L20 OS=Rattus norvegicus OX=10116 GN=Mrpl20 PE=1 SV=1 | Mrpl20 | 14.1 | 17.525 | 2.2917 |
| B2RYU0 | "NADH dehydrogenase (Ubiquinone) 1 beta subcomplex, 2 (Predicted), isoform CRA_b OS=Rattus norvegicus OX=10116 GN=Ndufb2 PE=1 SV=1" | Ndufb2 | 18.1 | 11.842 | 2.109 |
| B2RYW9 | Fumarylacetoacetate hydrolase domain-containing protein 2 OS=Rattus norvegicus OX=10116 GN=Fahd2 PE=1 SV=1 | Fahd2 | 16.3 | 34.581 | 2.0733 |
| B2RZ57 | Mitochondrial ribosomal protein L18 OS=Rattus norvegicus OX=10116 GN=Mrpl18 PE=1 SV=1 | Mrpl18 | 7.2 | 20.65 | 1.7387 |
| B2RZ76 | Dehydrogenase/reductase X-linked OS=Rattus norvegicus OX=10116 GN=Dhrsx PE=1 SV=1 | Dhrsx | 13.1 | 33.375 | 8.2238 |
| B2RZB3 | Eva-1 homolog B OS=Rattus norvegicus OX=10116 GN=Eva1b PE=1 SV=1 | Eva1b | 9.8 | 24.577 | 1.9617 |
| B2RZD0 | Mitochondrial ribosomal protein 63 OS=Rattus norvegicus OX=10116 GN=Mrpl57 PE=1 SV=1 | Mrpl57 | 28.4 | 12.039 | 4.0195 |
| B4F7A1 | Complex III assembly factor LYRM7 OS=Rattus norvegicus OX=10116 GN=Lyrm7 PE=3 SV=1 | Lyrm7 | 18.3 | 12.03 | 3.1317 |
| B5DEP4 | "28S ribosomal protein L42, mitochondrial OS=Rattus norvegicus OX=10116 GN=Mrpl42 PE=1 SV=1" | Mrpl42 | 9.2 | 16.564 | 2.0882 |
| B5DER5 | "Coiled-coil-helix-coiled-coil-helix domain containing 1 (Predicted), isoform CRA_a OS=Rattus norvegicus OX=10116 GN=Chchd1 PE=1 SV=1" | Chchd1 | 13.6 | 13.407 | 2.1036 |
| B5DFI3 | AP complex subunit sigma OS=Rattus norvegicus OX=10116 GN=Ap1s1 PE=1 SV=1 | Ap1s1 | 10.1 | 18.733 | 3.7977 |
| D3ZBG6 | Peptidyl-tRNA hydrolase domain-containing 1 OS=Rattus norvegicus OX=10116 GN=Ptrhd1 PE=1 SV=1 | Ptrhd1 | 21.4 | 16.026 | 7.3072 |
| D3ZDX7 | Mitochondrial ribosomal protein L48 OS=Rattus norvegicus OX=10116 GN=Mrpl48 PE=1 SV=1 | Mrpl48 | 5.7 | 24.019 | 2.0371 |
| D3ZDZ7 | Fucokinase OS=Rattus norvegicus OX=10116 GN=Fuk PE=1 SV=1 | Fuk | 3.8 | 111.18 | 2.4577 |
| D3ZE91 | "Potassium channel tetramerisation domain containing 9 (Predicted), isoform CRA_b OS=Rattus norvegicus OX=10116 GN=Kctd9 PE=4 SV=1" | Kctd9 | 4.6 | 42.502 | 3.2738 |
| D3ZE97 | Uncharacterized protein OS=Rattus norvegicus OX=10116 PE=4 SV=1 | --- | 23.1 | 18.452 | 1.654 |
| D3ZF99 | Mitochondrial ribosomal protein L55 OS=Rattus norvegicus OX=10116 GN=Mrpl55 PE=1 SV=2 | Mrpl55 | 12.6 | 15.062 | 4.4143 |
| D3ZJN9 | Ecotropic viral integration site 5 OS=Rattus norvegicus OX=10116 GN=Evi5 PE=1 SV=3 | Evi5 | 4.9 | 92.862 | 5.1629 |
| D3ZLF0 | GTP-binding protein 6 (putative) OS=Rattus norvegicus OX=10116 GN=Gtpbp6 PE=1 SV=1 | Gtpbp6 | 3.5 | 56.551 | 1.7008 |
| D3ZMR1 | Translocase of outer mitochondrial membrane 7 OS=Rattus norvegicus OX=10116 GN=Tomm7 PE=1 SV=1 | Tomm7 | 30.9 | 6.1773 | 2.6 |
| D3ZT98 | BolA family member 3 OS=Rattus norvegicus OX=10116 GN=Bola3 PE=1 SV=1 | Bola3 | 18.2 | 12.297 | 16.66 |
| D3ZUL4 | "B-cell CLL/lymphoma 7C (Predicted), isoform CRA_a OS=Rattus norvegicus OX=10116 GN=Bcl7c PE=1 SV=1" | Bcl7c | 12.4 | 23.485 | 3.1064 |
| D3ZXF8 | Mitochondrial ribosomal protein L43 OS=Rattus norvegicus OX=10116 GN=Mrpl43 PE=1 SV=3 | Mrpl43 | 20.5 | 17.575 | 1.8186 |
| D4A2G7 | ELL associated factor 1 (Predicted) OS=Rattus norvegicus OX=10116 GN=Eaf1 PE=1 SV=1 | Eaf1 | 4.8 | 38.25 | 2.5856 |
| D4A3T2 | GATA zinc finger domain-containing 1 OS=Rattus norvegicus OX=10116 GN=Gatad1 PE=4 SV=1 | Gatad1 | 12.9 | 28.409 | 2.572 |
| D4A3V6 | Biogenesis of lysosome-related organelles complex 1 subunit 3 OS=Rattus norvegicus OX=10116 GN=Bloc1s3 PE=1 SV=1 | Bloc1s3 | 17.4 | 20.3 | 4.3145 |
| D4A5Q9 | Glycine decarboxylase OS=Rattus norvegicus OX=10116 GN=Gldc PE=1 SV=1 | Gldc | 27.1 | 98.547 | 19.985 |
| D4A6D7 | "Tetratricopeptide repeat protein 19, mitochondrial OS=Rattus norvegicus OX=10116 GN=Ttc19 PE=3 SV=1" | Ttc19 | 11.2 | 41.333 | 2.8969 |
| D4A746 | GDP-mannose pyrophosphorylase B OS=Rattus norvegicus OX=10116 GN=Gmppb PE=1 SV=1 | Gmppb | 5 | 39.902 | 2.8944 |
| D4A7L4 | "NADH dehydrogenase (Ubiquinone) 1 beta subcomplex, 11 (Predicted) OS=Rattus norvegicus OX=10116 GN=Ndufb11 PE=1 SV=1" | Ndufb11 | 38.4 | 17.634 | 23.405 |
| D4A8N2 | Ferredoxin 1-like OS=Rattus norvegicus OX=10116 GN=Fdx2 PE=1 SV=1 | Fdx2 | 13.8 | 18.77 | 1.5424 |
| D4A904 | N-acetylglutamate synthase OS=Rattus norvegicus OX=10116 GN=Nags PE=1 SV=1 | Nags | 4.4 | 57.42 | -2 |
| D4A9K3 | D-aminoacyl-tRNA deacylase OS=Rattus norvegicus OX=10116 GN=Dtd1 PE=1 SV=1 | Dtd1 | 12.6 | 19.636 | 2.0557 |
| D4A9V7 | "Methionyl-tRNA synthetase 2, mitochondrial OS=Rattus norvegicus OX=10116 GN=LOC100911305 PE=1 SV=1" | LOC100911305 | 4.4 | 65.752 | 3.5859 |
| F1LMM8 | "[Pyruvate dehydrogenase (acetyl-transferring)] kinase isozyme 2, mitochondrial OS=Rattus norvegicus OX=10116 GN=Pdk2 PE=1 SV=3" | Pdk2 | 3.9 | 46.239 | 3.2562 |
| F1LNG8 | Protein Mpv17 OS=Rattus norvegicus OX=10116 GN=Mpv17 PE=1 SV=2 | Mpv17 | 13.6 | 18.75 | 1.6671 |
| F1LYI7 | Transmembrane protein 256 OS=Rattus norvegicus OX=10116 GN=Tmem256 PE=1 SV=2 | Tmem256 | 24.8 | 11.776 | 2.9109 |
| F1M772 | Uncharacterized protein OS=Rattus norvegicus OX=10116 GN=RGD1560723 PE=4 SV=2 | RGD1560723 | 9.1 | 17.81 | 4.664 |
| F1M7S2 | Proteasome inhibitor PI31 subunit OS=Rattus norvegicus OX=10116 GN=Psmf1 PE=1 SV=2 | Psmf1 | 12.2 | 29.882 | 2.8576 |
| F1M9P0 | Adaptor-related protein complex 1-associated regulatory protein OS=Rattus norvegicus OX=10116 GN=Ap1ar PE=4 SV=3 | Ap1ar | 10.1 | 33.736 | 2.3823 |
| F7F557 | Acyl-coenzyme A thioesterase 8 OS=Rattus norvegicus OX=10116 GN=Acot8 PE=1 SV=1 | Acot8 | 8.8 | 42.025 | 1.5425 |
| F7F588 | Nicotinamide-nucleotide adenylyltransferase OS=Rattus norvegicus OX=10116 GN=Nmnat3 PE=1 SV=1 | Nmnat3 | 7.8 | 27.577 | 1.5895 |
| G3V6R7 | "3-oxoacyl-[acyl-carrier-protein] synthase, mitochondrial OS=Rattus norvegicus OX=10116 GN=Oxsm PE=1 SV=1" | Oxsm | 5 | 48.004 | 3.7614 |
| G3V7L0 | "Adrenodoxin, mitochondrial OS=Rattus norvegicus OX=10116 GN=Fdx1 PE=1 SV=1" | Fdx1 | 17.6 | 20.149 | 1.9508 |
| G3V8W9 | Similar to CG12279-PA OS=Rattus norvegicus OX=10116 GN=Tstd3 PE=1 SV=1 | Tstd3 | 13.4 | 17.536 | 2.0005 |
| G3V981 | Similar to RIKEN cDNA A430005L14 OS=Rattus norvegicus OX=10116 GN=RGD1304567 PE=1 SV=1 | RGD1304567 | 9 | 23.653 | 3.1863 |
| G3V996 | LETM1 domain-containing 1 OS=Rattus norvegicus OX=10116 GN=Letmd1 PE=1 SV=1 | Letmd1 | 8.9 | 41.967 | 2.2663 |
| G3V9I7 | AarF domain-containing kinase 5 OS=Rattus norvegicus OX=10116 GN=Adck5 PE=1 SV=2 | Adck5 | 5.3 | 64.473 | 1.64 |
| H8Y6S5 | GRINL1A combined protein 15 OS=Rattus norvegicus OX=10116 GN=Polr2m PE=2 SV=1 | Polr2m | 4.9 | 86.894 | 1.8149 |
| M0R3K2 | "Glutamyl-tRNA(Gln) amidotransferase subunit C, mitochondrial OS=Rattus norvegicus OX=10116 GN=Gatc PE=1 SV=2" | Gatc | 17 | 16.965 | 3.1505 |
| M0R5E8 | Methenyltetrahydrofolate synthetase domain containing OS=Rattus norvegicus OX=10116 GN=Mthfsd PE=1 SV=2 | Mthfsd | 6 | 40.164 | 1.7115 |
| M0R6Q9 | Decaprenyl diphosphate synthase subunit 1 OS=Rattus norvegicus OX=10116 GN=Pdss1 PE=3 SV=1 | Pdss1 | 4.4 | 45.735 | 2.9873 |
| M0R7R2 | Similar to Protein C6orf203 OS=Rattus norvegicus OX=10116 GN=LOC683897 PE=1 SV=1 | LOC683897 | 14.6 | 29.264 | 4.9556 |
| O88994 | Mitochondrial amidoxime reducing component 2 OS=Rattus norvegicus OX=10116 GN=Marc2 PE=2 SV=1 | Marc2 | 20.1 | 38.248 | 2.3347 |
| P07633 | "Propionyl-CoA carboxylase beta chain, mitochondrial OS=Rattus norvegicus OX=10116 GN=Pccb PE=2 SV=1" | Pccb | 21.6 | 58.626 | 4.2587 |
| P10715 | "Cytochrome c, testis-specific OS=Rattus norvegicus OX=10116 GN=Cyct PE=2 SV=2" | Cyct | 17.1 | 11.742 | 1.9693 |
| P35171 | "Cytochrome c oxidase subunit 7A2, mitochondrial OS=Rattus norvegicus OX=10116 GN=Cox7a2 PE=1 SV=1" | Cox7a2 | 15.7 | 9.3529 | 23.075 |
| P62859 | 40S ribosomal protein S28 OS=Rattus norvegicus OX=10116 GN=Rps28 PE=1 SV=1 | Rps28 | 33.3 | 7.8409 | 70.568 |
| Q06C60 | BolA family member 1 OS=Rattus norvegicus OX=10116 GN=Bola1 PE=1 SV=1 | Bola1 | 12.4 | 14.403 | 4.5383 |
| Q3MIB4 | "Lon protease homolog 2, peroxisomal OS=Rattus norvegicus OX=10116 GN=Lonp2 PE=1 SV=2" | Lonp2 | 2.3 | 94.392 | 1.8356 |
| Q4KM92 | tRNA pseudouridine synthase A OS=Rattus norvegicus OX=10116 GN=Pus1 PE=1 SV=2 | Pus1 | 7.3 | 47.515 | 3.0896 |
| Q4V794 | "Similar to DNA segment, Chr 8, ERATO Doi 531, expressed, isoform CRA_c OS=Rattus norvegicus OX=10116 GN=Vps37a PE=1 SV=1" | Vps37a | 5 | 44.487 | 2.4736 |
| Q5EBA0 | "GTPase Era, mitochondrial OS=Rattus norvegicus OX=10116 GN=Eral1 PE=2 SV=2" | Eral1 | 8.7 | 48.379 | 2.0356 |
| Q5I0C5 | "Methionyl-tRNA formyltransferase, mitochondrial OS=Rattus norvegicus OX=10116 GN=Mtfmt PE=2 SV=1" | Mtfmt | 6.8 | 42.997 | 1.809 |
| Q5M949 | Nipsnap homolog 3A (C. elegans) OS=Rattus norvegicus OX=10116 GN=Nipsnap3b PE=1 SV=1 | Nipsnap3b | 10.5 | 28.34 | 2.454 |
| Q5PPI6 | Mitochondrial genome maintenance exonuclease 1 OS=Rattus norvegicus OX=10116 GN=Mgme1 PE=1 SV=1 | Mgme1 | 6.5 | 38.658 | 1.5062 |
| Q5PQP2 | Receptor-binding cancer antigen expressed on SiSo cells OS=Rattus norvegicus OX=10116 GN=Ebag9 PE=1 SV=1 | Ebag9 | 8.9 | 24.187 | 8.0626 |
| Q5RKI9 | "Ribosome-recycling factor, mitochondrial OS=Rattus norvegicus OX=10116 GN=Mrrf PE=2 SV=1" | Mrrf | 19.8 | 29.152 | 5.2571 |
| Q5U1Z2 | Trafficking protein particle complex subunit 3 OS=Rattus norvegicus OX=10116 GN=Trappc3 PE=2 SV=1 | Trappc3 | 15.6 | 20.302 | 2.7441 |
| Q5U2R1 | Decaprenyl-diphosphate synthase subunit 2 OS=Rattus norvegicus OX=10116 GN=Pdss2 PE=2 SV=1 | Pdss2 | 4.2 | 44.294 | 2.0528 |
| Q5XFX7 | RAD23 homolog A (S. cerevisiae) OS=Rattus norvegicus OX=10116 GN=Rad23a PE=1 SV=1 | Rad23a | 4.6 | 38.067 | 1.6701 |
| Q5XIA8 | Growth hormone-inducible transmembrane protein OS=Rattus norvegicus OX=10116 GN=Ghitm PE=2 SV=1 | Ghitm | 10.4 | 37.178 | 2.5813 |
| Q5XIJ4 | Protein FAM210A OS=Rattus norvegicus OX=10116 GN=Fam210a PE=2 SV=1 | Fam210a | 9.2 | 31.474 | 2.4262 |
| Q62651 | "Delta(3,5)-Delta(2,4)-dienoyl-CoA isomerase, mitochondrial OS=Rattus norvegicus OX=10116 GN=Ech1 PE=1 SV=2" | Ech1 | 13.1 | 36.171 | 3.0484 |
| Q6AY04 | "m-AAA protease-interacting protein 1, mitochondrial OS=Rattus norvegicus OX=10116 GN=Maip1 PE=2 SV=1" | Maip1 | 8.6 | 33.09 | 1.6253 |
| Q6AY72 | UPF0449 protein C19orf25 homolog OS=Rattus norvegicus OX=10116 PE=3 SV=1 | --- | 33 | 12.142 | 12.809 |
| Q6AY77 | Gem (nuclear organelle)-associated protein 8 OS=Rattus norvegicus OX=10116 GN=Gemin8 PE=2 SV=1 | Gemin8 | 12.6 | 29.024 | 4.8753 |
| Q6IM78 | "DNA topoisomerase I, mitochondrial OS=Rattus norvegicus OX=10116 GN=Top1mt PE=2 SV=1" | Top1mt | 3.9 | 69.01 | 1.5062 |
| Q6TUF2 | "Succinate dehydrogenase assembly factor 3, mitochondrial OS=Rattus norvegicus OX=10116 GN=Sdhaf3 PE=2 SV=2" | Sdhaf3 | 19.2 | 14.525 | 2.3883 |
| Q9JHL7 | Carcinoembryonic antigen-related cell adhesion molecule 1 OS=Rattus norvegicus OX=10116 GN=Ceacam1 PE=1 SV=1 | Ceacam1 | 6.7 | 50.44 | 4.1514 |
| Q9WVR8 | Menin OS=Rattus norvegicus OX=10116 GN=Men1 PE=1 SV=1 | Men1 | 2.6 | 67.335 | 4.1195 |
| Q9Z2X5 | Homer protein homolog 3 OS=Rattus norvegicus OX=10116 GN=Homer3 PE=1 SV=2 | Homer3 | 5.9 | 39.891 | 1.9026 |
| Q5I0C9 | "Cirrhosis, autosomal recessive 1A (Human) OS=Rattus norvegicus OX=10116 GN=Utp4 PE=1 SV=1" | Utp4 | 31.6 | 76.862 | 39.195 |
| D3ZN95 | Host cell factor C1 OS=Rattus norvegicus OX=10116 GN=Hcfc1 PE=1 SV=1 | Hcfc1 | 21.9 | 209.15 | 279.86 |
| D4A1H8 | "PWP1 homolog, endonuclein OS=Rattus norvegicus OX=10116 GN=Pwp1 PE=1 SV=1" | Pwp1 | 15.4 | 55.809 | 6.3862 |
| F1LNF3 | "Nuclear transcription factor, X-box-binding-like 1 OS=Rattus norvegicus OX=10116 GN=Nfxl1 PE=1 SV=1" | Nfxl1 | 4.9 | 101.92 | 3.1387 |
| Q9JMB5 | Proteasomal ubiquitin receptor ADRM1 OS=Rattus norvegicus OX=10116 GN=Adrm1 PE=1 SV=2 | Adrm1 | 19.4 | 42.102 | 25.087 |
| M0R3M4 | RAN-binding protein 2 OS=Rattus norvegicus OX=10116 GN=Ranbp2 PE=1 SV=1 | Ranbp2 | 33.5 | 344.39 | 323.31 |
| P61980 | Heterogeneous nuclear ribonucleoprotein K OS=Rattus norvegicus OX=10116 GN=Hnrnpk PE=1 SV=1 | Hnrnpk | 65.2 | 50.976 | 323.31 |
| G3V8L3 | "Lamin A, isoform CRA_b OS=Rattus norvegicus OX=10116 GN=Lmna PE=1 SV=1" | Lmna | 66.2 | 74.311 | 323.31 |
| P27008 | Poly [ADP-ribose] polymerase 1 OS=Rattus norvegicus OX=10116 GN=Parp1 PE=1 SV=4 | Parp1 | 53.7 | 112.66 | 323.31 |
| D3ZAQ6 | Nucleolar protein 7 OS=Rattus norvegicus OX=10116 GN=Nol7 PE=1 SV=3 | Nol7 | 16.2 | 33.624 | 19.698 |
| Q3KR55 | "RCG60540, isoform CRA_a OS=Rattus norvegicus OX=10116 GN=U2af1 PE=1 SV=1" | U2af1 | 49 | 27.815 | 200.49 |
| Q63014 | A-kinase anchor protein 8 OS=Rattus norvegicus OX=10116 GN=Akap8 PE=1 SV=1 | Akap8 | 19.5 | 76.161 | 86.649 |
| D4AB23 | Nucleolar complex protein 3 homolog OS=Rattus norvegicus OX=10116 GN=Noc3l PE=1 SV=1 | Noc3l | 19.3 | 92.424 | 44.084 |
| Q5XIP6 | Flap endonuclease 1 OS=Rattus norvegicus OX=10116 GN=Fen1 PE=2 SV=1 | Fen1 | 41.6 | 42.608 | 164.13 |
| D3ZD05 | KN motif and ankyrin repeat domains 2 OS=Rattus norvegicus OX=10116 GN=Kank2 PE=1 SV=2 | Kank2 | 49.1 | 90.883 | 323.31 |
| Q01986 | Dual specificity mitogen-activated protein kinase kinase 1 OS=Rattus norvegicus OX=10116 GN=Map2k1 PE=1 SV=2 | Map2k1 | 52.9 | 43.465 | 112.8 |
| G3V8L9 | Caveolae-associated protein 1 OS=Rattus norvegicus OX=10116 GN=Cavin1 PE=1 SV=1 | Cavin1 | 43.1 | 43.908 | 323.31 |
| G3V741 | "Phosphate carrier protein, mitochondrial OS=Rattus norvegicus OX=10116 GN=Slc25a3 PE=1 SV=1" | Slc25a3 | 43.5 | 39.531 | 285.46 |
| F2Z3T9 | U2 snRNP auxiliary factor large subunit OS=Rattus norvegicus OX=10116 GN=U2af2 PE=1 SV=2 | U2af2 | 47.6 | 53.12 | 323.31 |
| F1LNF0 | Myosin heavy chain 14 OS=Rattus norvegicus OX=10116 GN=Myh14 PE=1 SV=1 | Myh14 | 30.4 | 228.91 | 131.88 |
| Q6MGC3 | BING4 protein OS=Rattus norvegicus OX=10116 GN=Wdr46 PE=1 SV=1 | Wdr46 | 25.3 | 67.472 | 25.87 |
| A0A0G2K9A7 | WD repeat domain 75 OS=Rattus norvegicus OX=10116 GN=Wdr75 PE=1 SV=1 | Wdr75 | 33.8 | 94.203 | 112.18 |
| D3Z9L0 | Acylglycerol kinase OS=Rattus norvegicus OX=10116 GN=Agk PE=1 SV=2 | Agk | 13.1 | 54.258 | 50.172 |
| D3ZBL3 | Neuroguidin OS=Rattus norvegicus OX=10116 GN=Ngdn PE=1 SV=1 | Ngdn | 31.4 | 35.814 | 39.324 |
| Q5BJS0 | ATP-dependent RNA helicase DHX30 OS=Rattus norvegicus OX=10116 GN=Dhx30 PE=1 SV=1 | Dhx30 | 29.8 | 134 | 38.562 |
| P17325 | Transcription factor AP-1 OS=Rattus norvegicus OX=10116 GN=Jun PE=1 SV=1 | Jun | 14.4 | 36 | 7.4917 |
| Q60587 | "Trifunctional enzyme subunit beta, mitochondrial OS=Rattus norvegicus OX=10116 GN=Hadhb PE=1 SV=1" | Hadhb | 53.1 | 51.414 | 274.89 |
| D4A7N1 | MICOS complex subunit Mic25 OS=Rattus norvegicus OX=10116 GN=Chchd6 PE=1 SV=1 | Chchd6 | 34.1 | 29.211 | 44.672 |
| P43138 | DNA-(apurinic or apyrimidinic site) lyase OS=Rattus norvegicus OX=10116 GN=Apex1 PE=1 SV=2 | Apex1 | 59.3 | 35.538 | 270.9 |
| Q5U2M4 | DNA ligase OS=Rattus norvegicus OX=10116 GN=Lig3 PE=1 SV=1 | Lig3 | 24.4 | 105.44 | 65.007 |
| Q5PQZ9 | NADH dehydrogenase [ubiquinone] 1 subunit C2 OS=Rattus norvegicus OX=10116 GN=Ndufc2 PE=1 SV=1 | Ndufc2 | 19.2 | 14.359 | 51.751 |
| D3ZTR5 | RCG21454 OS=Rattus norvegicus OX=10116 GN=Zbed5 PE=4 SV=1 | Zbed5 | 8.2 | 84.034 | 83.141 |
| Q56A27 | Nuclear cap-binding protein subunit 1 OS=Rattus norvegicus OX=10116 GN=Ncbp1 PE=1 SV=1 | Ncbp1 | 36.1 | 91.91 | 47.736 |
| D4A4K4 | Vacuolar protein sorting 13 homolog C OS=Rattus norvegicus OX=10116 GN=Vps13c PE=1 SV=2 | Vps13c | 13.2 | 418.62 | 79.533 |
| B1WC75 | DLG-associated protein 5 OS=Rattus norvegicus OX=10116 GN=Dlgap5 PE=2 SV=1 | Dlgap5 | 30.4 | 90.52 | 66.081 |
| F1LX07 | Solute carrier family 25 member 12 OS=Rattus norvegicus OX=10116 GN=Slc25a12 PE=1 SV=3 | Slc25a12 | 43.4 | 71.916 | 221.44 |
| O08623 | Sequestosome-1 OS=Rattus norvegicus OX=10116 GN=Sqstm1 PE=1 SV=1 | Sqstm1 | 49.4 | 47.681 | 63.203 |
| Q9Z2P5 | Receptor-interacting serine/threonine-protein kinase 3 OS=Rattus norvegicus OX=10116 GN=Ripk3 PE=1 SV=3 | Ripk3 | 34.3 | 52.217 | 67.251 |
| D3ZL85 | Cytochrome c heme lyase OS=Rattus norvegicus OX=10116 GN=Hccs PE=1 SV=1 | Hccs | 47.1 | 31.129 | 23.336 |
| Q6AXV0 | Polynucleotide kinase 3'-phosphatase OS=Rattus norvegicus OX=10116 GN=Pnkp PE=1 SV=1 | Pnkp | 39.3 | 57.186 | 28.82 |
| P18395 | Cold shock domain-containing protein E1 OS=Rattus norvegicus OX=10116 GN=Csde1 PE=2 SV=1 | Csde1 | 27.9 | 88.894 | 68.124 |
| P06536 | Glucocorticoid receptor OS=Rattus norvegicus OX=10116 GN=Nr3c1 PE=1 SV=2 | Nr3c1 | 15.3 | 87.555 | 16.208 |
| B2RYS2 | Cytochrome b-c1 complex subunit 7 OS=Rattus norvegicus OX=10116 GN=Uqcrb PE=1 SV=1 | Uqcrb | 53.2 | 13.558 | 111.74 |
| D4A1D3 | Sacsin molecular chaperone OS=Rattus norvegicus OX=10116 GN=Sacs PE=1 SV=2 | Sacs | 7.4 | 521.48 | 27.98 |
| B4F7A9 | Casein kinase 2 alpha 2 OS=Rattus norvegicus OX=10116 GN=Csnk2a2 PE=1 SV=1 | Csnk2a2 | 54.3 | 41.201 | 57.906 |
| D4ADE7 | CD3e molecule-associated protein OS=Rattus norvegicus OX=10116 GN=Cd3eap PE=1 SV=1 | Cd3eap | 29.9 | 49.412 | 42.035 |
| A0A0G2K4G0 | Retinoic acid-induced 1 OS=Rattus norvegicus OX=10116 GN=Rai1 PE=1 SV=1 | Rai1 | 3.3 | 201.06 | 4.2151 |
| A0A0G2QC41 | Histone deacetylase OS=Rattus norvegicus OX=10116 GN=Hdac6 PE=1 SV=1 | Hdac6 | 21.9 | 125.49 | 29.546 |
| D3ZYL0 | Receptor (TNFRSF)-interacting serine-threonine kinase 1 (Predicted) OS=Rattus norvegicus OX=10116 GN=Ripk1 PE=1 SV=1 | Ripk1 | 13.4 | 74.756 | 4.0588 |
| D4A208 | SLIT-ROBO Rho GTPase-activating protein 2 OS=Rattus norvegicus OX=10116 GN=Srgap2 PE=1 SV=1 | Srgap2 | 37.8 | 120.88 | 79.262 |
| A0A0G2K1W1 | RAB11 family-interacting protein 5 OS=Rattus norvegicus OX=10116 GN=Rab11fip5 PE=1 SV=1 | Rab11fip5 | 25.4 | 123.81 | 62.418 |
| F1LP46 | "ATP-dependent RNA helicase SUPV3L1, mitochondrial OS=Rattus norvegicus OX=10116 GN=Supv3l1 PE=1 SV=2" | Supv3l1 | 10.4 | 78.922 | 7.859 |
| A0A0G2K1Q1 | NLR family member X1 OS=Rattus norvegicus OX=10116 GN=Nlrx1 PE=1 SV=1 | Nlrx1 | 9.7 | 107.33 | 9.0362 |
| B3DMA2 | Acyl-CoA dehydrogenase family member 11 OS=Rattus norvegicus OX=10116 GN=Acad11 PE=1 SV=1 | Acad11 | 3.9 | 87.37 | 1.8729 |
| A0A0G2JU49 | Metaxin 1 OS=Rattus norvegicus OX=10116 GN=Mtx1 PE=1 SV=1 | Mtx1 | 31.1 | 51.925 | 76.158 |
| A0A0H2UHK3 | "FAST kinase domain-containing protein 2, mitochondrial OS=Rattus norvegicus OX=10116 GN=Fastkd2 PE=1 SV=1" | Fastkd2 | 10.2 | 78.893 | 8.8086 |
| P14056 | Serine/threonine-protein kinase A-Raf OS=Rattus norvegicus OX=10116 GN=Araf PE=1 SV=1 | Araf | 8.8 | 67.551 | 4.8262 |
| Q5U312 | Ankycorbin OS=Rattus norvegicus OX=10116 GN=Rai14 PE=1 SV=2 | Rai14 | 38.9 | 109.13 | 132.67 |
| Q9NQR8 | NADH dehydrogenase [ubiquinone] 1 alpha subcomplex assembly factor 4 OS=Rattus norvegicus OX=10116 GN=Ndufaf4 PE=1 SV=1 | Ndufaf4 | 38.5 | 20.158 | 6.312 |
| Q6TUH0 | "2',5'-phosphodiesterase 12 OS=Rattus norvegicus OX=10116 GN=Pde12 PE=1 SV=1" | Pde12 | 9.6 | 78.885 | 5.2713 |
| Q5XIG0 | "ADP-ribose pyrophosphatase, mitochondrial OS=Rattus norvegicus OX=10116 GN=Nudt9 PE=2 SV=1" | Nudt9 | 7.4 | 38.562 | 2.5134 |
| Q9ES71 | Dihydroxyacetone phosphate acyltransferase OS=Rattus norvegicus OX=10116 GN=Gnpat PE=1 SV=1 | Gnpat | 14 | 77.075 | 8.2161 |
| A0A0G2JTL5 | "Pyruvate carboxylase, mitochondrial OS=Rattus norvegicus OX=10116 GN=Pc PE=1 SV=1" | Pc | 38.4 | 140 | 99.159 |
| Q5XIM0 | "BCS1 homolog, ubiquinol-cytochrome c reductase complex chaperone OS=Rattus norvegicus OX=10116 GN=Bcs1l PE=1 SV=1" | Bcs1l | 34.7 | 47.393 | 107.12 |
| D3ZHW0 | ATP-dependent RNA helicase DHX29 OS=Rattus norvegicus OX=10116 GN=Dhx29 PE=1 SV=2 | Dhx29 | 24.7 | 154.12 | 54.694 |
| D3ZJB8 | RBR-type E3 ubiquitin transferase OS=Rattus norvegicus OX=10116 GN=Arih2 PE=1 SV=1 | Arih2 | 19.9 | 57.74 | 11.02 |
| Q5XIG4 | OCIA domain-containing protein 1 OS=Rattus norvegicus OX=10116 GN=Ociad1 PE=1 SV=1 | Ociad1 | 53.8 | 27.659 | 36.438 |
| D3ZHK4 | RB1-inducible coiled-coil 1 OS=Rattus norvegicus OX=10116 GN=Rb1cc1 PE=1 SV=1 | Rb1cc1 | 12.4 | 182.23 | 14.92 |
| D4AE90 | RCC1-like OS=Rattus norvegicus OX=10116 GN=Rcc1l PE=1 SV=1 | Rcc1l | 11.1 | 50.113 | 1.9551 |
| A0A0G2K261 | "Isoleucyl-tRNA synthetase 2, mitochondrial OS=Rattus norvegicus OX=10116 GN=Iars2 PE=1 SV=1" | Iars2 | 30.7 | 112.68 | 122.47 |
| A0A0G2KAL9 | Mitochondrial fission factor OS=Rattus norvegicus OX=10116 GN=Mff PE=1 SV=1 | Mff | 35.4 | 32.801 | 27.585 |
| Q5U300 | Ubiquitin-like modifier-activating enzyme 1 OS=Rattus norvegicus OX=10116 GN=Uba1 PE=1 SV=1 | Uba1 | 42 | 117.79 | 323.31 |
| D4A3T4 | Zinc finger FYVE-type-containing 1 OS=Rattus norvegicus OX=10116 GN=Zfyve1 PE=1 SV=1 | Zfyve1 | 10.4 | 86.903 | 6.0853 |
| A0A0G2JWK2 | Methyl-CpG-binding protein 2 OS=Rattus norvegicus OX=10116 GN=Mecp2 PE=1 SV=1 | Mecp2 | 6.5 | 53.048 | 2.4964 |
| F1LNB3 | A-kinase-anchoring protein 10 OS=Rattus norvegicus OX=10116 GN=Akap10 PE=1 SV=1 | Akap10 | 12.4 | 73.773 | 3.5665 |
| Q5FWT5 | "Glutamyl-tRNA(Gln) amidotransferase subunit A, mitochondrial OS=Rattus norvegicus OX=10116 GN=Qrsl1 PE=2 SV=1" | Qrsl1 | 6.9 | 56.837 | 4.0464 |
| A0A0G2JVK4 | "Neurolysin, mitochondrial OS=Rattus norvegicus OX=10116 GN=Nln PE=1 SV=1" | Nln | 25 | 77.801 | 12.824 |
| D3ZD73 | DEAD-box helicase 6 OS=Rattus norvegicus OX=10116 GN=Ddx6 PE=1 SV=1 | Ddx6 | 61.3 | 54.244 | 323.31 |
| B0BN83 | Armadillo repeat-containing protein 1 OS=Rattus norvegicus OX=10116 GN=Armc1 PE=1 SV=1 | Armc1 | 47.2 | 31.204 | 36.049 |
| F1M3K6 | BCL2-associated athanogene 4 OS=Rattus norvegicus OX=10116 GN=Bag4 PE=1 SV=3 | Bag4 | 10.5 | 49.025 | 3.0496 |
| A0A0U1RRV5 | Clustered mitochondria protein homolog OS=Rattus norvegicus OX=10116 GN=Cluh PE=1 SV=1 | Cluh | 17.3 | 151.16 | 26.293 |
| Q80Z30 | Protein phosphatase 1E OS=Rattus norvegicus OX=10116 GN=Ppm1e PE=1 SV=1 | Ppm1e | 6.9 | 83.438 | 2.8968 |
| A0A0G2K714 | Endophilin-B1 OS=Rattus norvegicus OX=10116 GN=Sh3glb1 PE=1 SV=1 | Sh3glb1 | 20.8 | 43.078 | 21.247 |
| D4ACN6 | Collagen type IV alpha 3-binding protein OS=Rattus norvegicus OX=10116 GN=Col4a3bp PE=1 SV=2 | Col4a3bp | 16 | 71.068 | 8.3882 |
| D3ZPN5 | Mitochondrial poly(A) polymerase OS=Rattus norvegicus OX=10116 GN=Mtpap PE=1 SV=2 | Mtpap | 23.5 | 37.284 | 3.6632 |
| Q5XIC8 | Alpha-ketoglutarate-dependent dioxygenase alkB homolog 3 OS=Rattus norvegicus OX=10116 GN=Alkbh3 PE=2 SV=1 | Alkbh3 | 18.6 | 34.011 | 5.1506 |
| D3ZKQ4 | "RAB, member RAS oncogene family-like 6 OS=Rattus norvegicus OX=10116 GN=Rabl6 PE=1 SV=1" | Rabl6 | 7.9 | 80.253 | 4.5868 |
| F1M0A6 | Tyrosine-protein kinase OS=Rattus norvegicus OX=10116 GN=LOC100909750 PE=3 SV=1 | LOC100909750 | 4.6 | 122.24 | 2.5053 |
| G3V644 | "NADH dehydrogenase (Ubiquinone) flavoprotein 3-like, isoform CRA_a OS=Rattus norvegicus OX=10116 GN=Ndufv3 PE=1 SV=1" | Ndufv3 | 34.7 | 49.299 | 64.882 |
| A0A0A0MXZ5 | Ubiquitin carboxyl-terminal hydrolase BAP1 OS=Rattus norvegicus OX=10116 GN=Bap1 PE=1 SV=1 | Bap1 | 1.6 | 81.734 | 1.8712 |
| A0A0G2K2S3 | Mitochondrial fission regulator 2 OS=Rattus norvegicus OX=10116 GN=Bclaf1 PE=1 SV=1 | Bclaf1 | 12.6 | 184.78 | 2.0777 |
| D3ZKU7 | Biogenesis of lysosome-related organelles complex 1 subunit 1 OS=Rattus norvegicus OX=10116 GN=Bloc1s1 PE=3 SV=1 | Bloc1s1 | 36.8 | 14.311 | 5.1762 |
| D3ZMW3 | "Misato 1, mitochondrial distribution and morphology regulator OS=Rattus norvegicus OX=10116 GN=Msto1 PE=1 SV=1" | Msto1 | 9 | 60.633 | 2.3301 |
| D3ZY40 | PCF11 cleavage and polyadenylation factor subunit OS=Rattus norvegicus OX=10116 GN=Pcf11 PE=1 SV=1 | Pcf11 | 1.2 | 172.66 | 2.2305 |
| D4A175 | tRNA dimethylallyltransferase OS=Rattus norvegicus OX=10116 GN=Trit1 PE=3 SV=1 | Trit1 | 3.8 | 53.666 | 1.9513 |
| M0R4L6 | "Glutamyl-tRNA(Gln) amidotransferase subunit B, mitochondrial OS=Rattus norvegicus OX=10116 GN=Gatb PE=1 SV=1" | Gatb | 7.7 | 61.982 | 5.1262 |
| O08557 | "N(G),N(G)-dimethylarginine dimethylaminohydrolase 1 OS=Rattus norvegicus OX=10116 GN=Ddah1 PE=1 SV=3" | Ddah1 | 21.8 | 31.426 | 6.1701 |
| P15337 | Cyclic AMP-responsive element-binding protein 1 OS=Rattus norvegicus OX=10116 GN=Creb1 PE=1 SV=1 | Creb1 | 4.1 | 36.633 | 2.026 |
| Q3ZAU5 | DDHD domain-containing 1 OS=Rattus norvegicus OX=10116 GN=Ddhd1 PE=1 SV=1 | Ddhd1 | 3.5 | 95.173 | 2.1756 |
| Q568Y3 | E3 ubiquitin-protein ligase RNF185 OS=Rattus norvegicus OX=10116 GN=Rnf185 PE=2 SV=1 | Rnf185 | 13.5 | 20.493 | 4.1253 |
| Q66H85 | Ankyrin repeat and zinc finger domain-containing protein 1 OS=Rattus norvegicus OX=10116 GN=Ankzf1 PE=2 SV=1 | Ankzf1 | 6.8 | 80.584 | 2.5004 |
| Q63707 | "Dihydroorotate dehydrogenase (quinone), mitochondrial OS=Rattus norvegicus OX=10116 GN=Dhodh PE=1 SV=1" | Dhodh | 56.5 | 42.662 | 161.85 |
| Q5XI22 | "Acetyl-CoA acetyltransferase, cytosolic OS=Rattus norvegicus OX=10116 GN=Acat2 PE=1 SV=1" | Acat2 | 34.5 | 41.108 | 41.229 |
| D3ZT71 | BCL2-like 13 OS=Rattus norvegicus OX=10116 GN=Bcl2l13 PE=1 SV=3 | Bcl2l13 | 21.7 | 46.734 | 15.514 |
| A0A0G2K1D2 | "RAP1, GTP-GDP dissociation stimulator 1 (Predicted), isoform CRA_a OS=Rattus norvegicus OX=10116 GN=Rap1gds1 PE=1 SV=1" | Rap1gds1 | 23.1 | 57.511 | 13.322 |
| Q4G086 | BCL2-interacting protein 3-like OS=Rattus norvegicus OX=10116 GN=Bnip3l PE=1 SV=1 | Bnip3l | 17 | 23.646 | 10.433 |
| P04166 | Cytochrome b5 type B OS=Rattus norvegicus OX=10116 GN=Cyb5b PE=1 SV=2 | Cyb5b | 52.1 | 16.265 | 323.31 |
| Q7TSA0 | Mitochondrial Rho GTPase 2 OS=Rattus norvegicus OX=10116 GN=Rhot2 PE=1 SV=1 | Rhot2 | 25.7 | 69.152 | 31.204 |
| F1LPV8 | "Succinate--CoA ligase [GDP-forming] subunit beta, mitochondrial OS=Rattus norvegicus OX=10116 GN=Suclg2 PE=1 SV=2" | Suclg2 | 45 | 46.638 | 242.74 |
| Q75Q39 | Mitochondrial import receptor subunit TOM70 OS=Rattus norvegicus OX=10116 GN=Tomm70 PE=1 SV=1 | Tomm70 | 51.6 | 67.444 | 323.31 |
| Q3B7D0 | "Oxygen-dependent coproporphyrinogen-III oxidase, mitochondrial OS=Rattus norvegicus OX=10116 GN=Cpox PE=1 SV=1" | Cpox | 56.4 | 49.278 | 36.683 |
| Q5BJZ3 | Nicotinamide nucleotide transhydrogenase OS=Rattus norvegicus OX=10116 GN=Nnt PE=1 SV=1 | Nnt | 33.4 | 113.87 | 323.31 |
| P00406 | Cytochrome c oxidase subunit 2 OS=Rattus norvegicus OX=10116 GN=Mtco2 PE=1 SV=3 | Mtco2 | 37.4 | 25.928 | 77.475 |
| P26431 | Sodium/hydrogen exchanger 1 OS=Rattus norvegicus OX=10116 GN=Slc9a1 PE=1 SV=2 | Slc9a1 | 10.7 | 91.646 | 12.512 |
| Q9R066 | Coxsackievirus and adenovirus receptor homolog OS=Rattus norvegicus OX=10116 GN=Cxadr PE=1 SV=2 | Cxadr | 12.9 | 39.948 | 3.7123 |
| P08050 | Gap junction alpha-1 protein OS=Rattus norvegicus OX=10116 GN=Gja1 PE=1 SV=2 | Gja1 | 33.2 | 43.031 | 52.749 |
| B0BN30 | Mitochondrial carrier 1 OS=Rattus norvegicus OX=10116 GN=Mtch1 PE=1 SV=1 | Mtch1 | 28 | 41.592 | 19.148 |
| Q5RKI8 | "ATP-binding cassette sub-family B member 8, mitochondrial OS=Rattus norvegicus OX=10116 GN=Abcb8 PE=2 SV=1" | Abcb8 | 16.1 | 77.77 | 12.734 |
| A0A0G2K5L2 | Mitochondrial glutamate carrier 1-like OS=Rattus norvegicus OX=10116 GN=LOC100911440 PE=1 SV=1 | LOC100911440 | 26 | 34.662 | 90.426 |
| Q641Z9 | Succinate dehydrogenase complex subunit C OS=Rattus norvegicus OX=10116 GN=Sdhc PE=1 SV=1 | Sdhc | 21.3 | 18.202 | 19.773 |
| P53987 | Monocarboxylate transporter 1 OS=Rattus norvegicus OX=10116 GN=Slc16a1 PE=1 SV=1 | Slc16a1 | 18 | 53.238 | 323.31 |
| Q66HP8 | Mitochondrial carnitine/acylcarnitine carrier protein OS=Rattus norvegicus OX=10116 GN=Slc25a20 PE=1 SV=1 | Slc25a20 | 38.5 | 33.071 | 26.972 |
| A0A0G2JUS7 | "Transmembrane protein 11, mitochondrial OS=Rattus norvegicus OX=10116 GN=Tmem11 PE=1 SV=1" | Tmem11 | 23.3 | 19.834 | 9.1741 |
| D3ZRH1 | "OXA1L, mitochondrial inner membrane protein OS=Rattus norvegicus OX=10116 GN=Oxa1l PE=1 SV=1" | Oxa1l | 11.1 | 48.023 | 6.2037 |
| A0A0G2JVW5 | E3 ubiquitin-protein ligase HUWE1 OS=Rattus norvegicus OX=10116 GN=Huwe1 PE=1 SV=1 | Huwe1 | 8.7 | 447.65 | 32.189 |
| Q7TT47 | Paraplegin OS=Rattus norvegicus OX=10116 GN=Spg7 PE=2 SV=2 | Spg7 | 25.2 | 86.102 | 22.369 |
| D4ACA5 | Mitochondrial import inner membrane translocase subunit TIM17 OS=Rattus norvegicus OX=10116 GN=LOC100911130 PE=1 SV=1 | LOC100911130 | 43.6 | 18.377 | 73.657 |
| G3V9P7 | Huntingtin OS=Rattus norvegicus OX=10116 GN=Htt PE=1 SV=1 | Htt | 2.5 | 344.74 | 5.5683 |
| Q5U2T5 | "RCG33556, isoform CRA_b OS=Rattus norvegicus OX=10116 GN=Stard3 PE=1 SV=1" | Stard3 | 20 | 50.385 | 10.205 |
| Q80W57 | ATP-binding cassette sub-family G member 2 OS=Rattus norvegicus OX=10116 GN=Abcg2 PE=1 SV=1 | Abcg2 | 10.8 | 72.96 | 8.03 |
| Q2IBC5 | Caveolin-2 OS=Rattus norvegicus OX=10116 GN=Cav2 PE=1 SV=2 | Cav2 | 34 | 18.266 | 42.681 |
| Q5XIF5 | Sphingolipid delta(4)-desaturase DES1 OS=Rattus norvegicus OX=10116 GN=Degs1 PE=2 SV=1 | Degs1 | 26.3 | 38.055 | 53.233 |
| Q2YDU8 | Protein spinster homolog 1 OS=Rattus norvegicus OX=10116 GN=Spns1 PE=2 SV=2 | Spns1 | 7.2 | 56.795 | 6.0717 |
| D3ZS74 | "Metalloendopeptidase OMA1, mitochondrial OS=Rattus norvegicus OX=10116 GN=Oma1 PE=3 SV=1" | Oma1 | 6 | 57.144 | 2.7315 |
| Q5FWU3 | Autophagy-related protein 9A OS=Rattus norvegicus OX=10116 GN=Atg9a PE=1 SV=1 | Atg9a | 13.1 | 94.487 | 8.3154 |
| O70595 | "ATP-binding cassette sub-family B member 6, mitochondrial OS=Rattus norvegicus OX=10116 GN=Abcb6 PE=2 SV=1" | Abcb6 | 11.1 | 93.304 | 26.26 |
| A0A0H2UHF6 | PRA1 family protein OS=Rattus norvegicus OX=10116 GN=Arl6ip5 PE=1 SV=1 | Arl6ip5 | 18.4 | 23.639 | 45.183 |
| B0BNF6 | Membrane-associated ring finger (C3HC4) 5 OS=Rattus norvegicus OX=10116 GN=March5 PE=1 SV=1 | March5 | 47.8 | 31.231 | 17.283 |
| G3V8T9 | Apoptosis regulator BAX OS=Rattus norvegicus OX=10116 GN=Bax PE=1 SV=1 | Bax | 40.6 | 21.444 | 28.519 |
| P97887 | Presenilin-1 OS=Rattus norvegicus OX=10116 GN=Psen1 PE=1 SV=1 | Psen1 | 11.3 | 52.789 | 10.83 |
| Q704E8 | "ATP-binding cassette sub-family B member 7, mitochondrial OS=Rattus norvegicus OX=10116 GN=Abcb7 PE=1 SV=1" | Abcb7 | 34.6 | 82.557 | 35.054 |
| P05508 | NADH-ubiquinone oxidoreductase chain 4 OS=Rattus norvegicus OX=10116 GN=Mtnd4 PE=3 SV=3 | Mtnd4 | 15.7 | 51.782 | 11.709 |
| G3V887 | V-type proton ATPase subunit a OS=Rattus norvegicus OX=10116 GN=Tcirg1 PE=1 SV=1 | Tcirg1 | 28.5 | 93.209 | 183.44 |
| P11661 | NADH-ubiquinone oxidoreductase chain 5 OS=Rattus norvegicus OX=10116 GN=Mtnd5 PE=3 SV=3 | Mtnd5 | 4.9 | 68.617 | 4.2144 |
| Q5FVL8 | ATP-binding cassette subfamily B member 10 OS=Rattus norvegicus OX=10116 GN=Abcb10 PE=1 SV=1 | Abcb10 | 7.4 | 77.393 | 9.8618 |
| D4A414 | COX15 cytochrome c oxidase assembly homolog OS=Rattus norvegicus OX=10116 GN=Cox15 PE=1 SV=1 | Cox15 | 20.8 | 45.914 | 9.6225 |
| D3ZG27 | UbiA prenyltransferase domain-containing protein 1 OS=Rattus norvegicus OX=10116 GN=Ubiad1 PE=3 SV=1 | Ubiad1 | 5.3 | 37.026 | 2.4831 |
| D3ZJ86 | Sodium/hydrogen exchanger OS=Rattus norvegicus OX=10116 GN=Slc9a6 PE=1 SV=2 | Slc9a6 | 6 | 79.837 | 38.032 |
| D4A1H7 | Mitochondrial E3 ubiquitin protein ligase 1 OS=Rattus norvegicus OX=10116 GN=Mul1 PE=1 SV=1 | Mul1 | 6.2 | 39.78 | 1.9854 |
| O35092 | Mitochondrial import inner membrane translocase subunit Tim17-A OS=Rattus norvegicus OX=10116 GN=Timm17a PE=2 SV=1 | Timm17a | 12.9 | 18.037 | 2.468 |
| P03889 | NADH-ubiquinone oxidoreductase chain 1 OS=Rattus norvegicus OX=10116 GN=Mtnd1 PE=1 SV=3 | Mtnd1 | 6 | 36.145 | 1.5858 |
| P05503 | Cytochrome c oxidase subunit 1 OS=Rattus norvegicus OX=10116 GN=Mtco1 PE=2 SV=3 | Mtco1 | 6.2 | 56.844 | 1.4964 |
| Q5RKH7 | Solute carrier family 35 member F6 OS=Rattus norvegicus OX=10116 GN=Slc35f6 PE=2 SV=1 | Slc35f6 | 9.1 | 41.092 | 3.2223 |
| Q6AY49 | Tumor suppressor candidate 3 OS=Rattus norvegicus OX=10116 GN=Tusc3 PE=1 SV=1 | Tusc3 | 6.9 | 39.538 | 2.4655 |
